# Novel triterpenoids from *Ganoderma resinaceum* attenuate UV-induced photoaging via modulating Nrf2 and MAPK signaling pathways

**DOI:** 10.1007/s13659-025-00558-z

**Published:** 2026-01-09

**Authors:** Yi Luo, Xiao-Cui Liu, Yu-Jie Li, Ming-Hua Qiu, Xing-Rong Peng

**Affiliations:** 1 State Key Laboratory of Phytochemistry and Natural Medicines, Kunming Institute of Botany, Chinese Academy of Sciences, Kunming, 650201 China; 2 Kunming College of Life Science, University of Chinese Academy of Sciences, Kunming, 650204 Yunnan China; 3 University of Chinese Academy of Sciences, Beijing, 100049 China

**Keywords:** *Ganoderma resinaceum*, Lanostane-type triterpenoids, Nrf2, MAPK signaling pathway, Skin photoaging

## Abstract

As a dual-purpose medicinal and edible mushroom, *Ganoderma* species have garnered significant interest in both the food, cosmetics and pharmaceutical industries. To further substantiate its traditional and functional uses, we conducted a systematic phytochemical study of *Ganoderma resinaceum* fruiting bodies, isolating 43 lanostane-type triterpenoids. Among these, 16 were identified as new compounds (**1**–**11**, **15**, **31**, **35**, **37**, and **42**). Compound **1** represents the first reported C₂₉ lanostane triterpenoid featuring a 21,24-cyclo five membered carbon ring fraction. The spectroscopic (1D/2D NMR, ESIMS) and X-ray crystallographic analyses confirmed their structures. Among these, compounds **2**–**4**, **13**, **17**, **35**, **36**, and **42** exhibited potent antioxidant activity by suppressing UV-induced ROS in skin keratinocytes. The most active compound, **42**, reduced ROS and malondialdehyde (MDA) levels, enhanced antioxidant defenses (superoxide dismutase, SOD; hydroxyproline), and suppressed matrix metalloproteinases (MMPs) through activating Nrf2 pathway and suppressing MAPK signaling. These results position *G. resinaceum* triterpenoids, particularly compound **42**, as multifunctional natural antioxidants with applications in functional foods for oxidative stress management or skin-protective formulations.

**Graphical Abstract:**

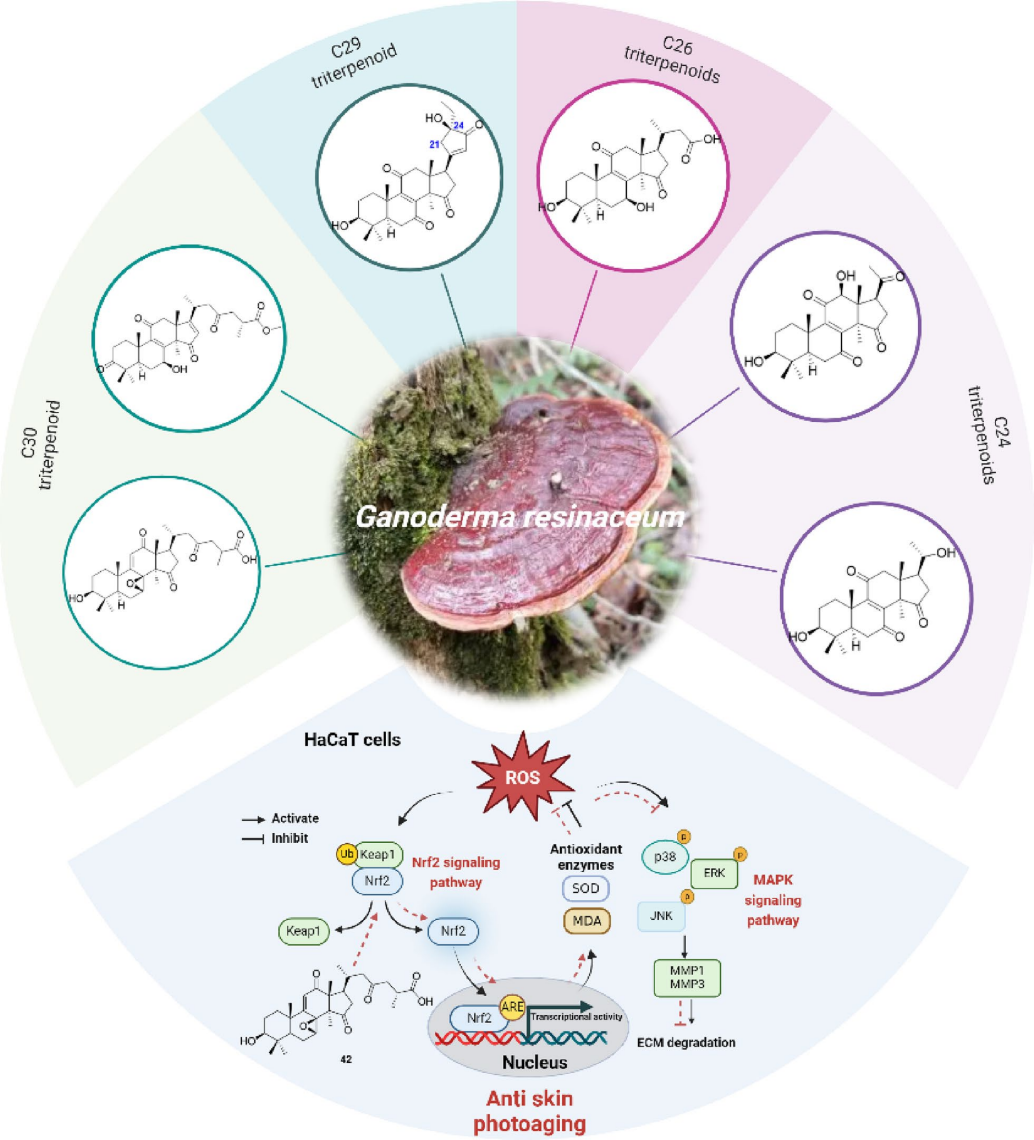

**Supplementary Information:**

The online version contains supplementary material available at 10.1007/s13659-025-00558-z.

## Introduction

*Ganoderma* species, especial *Ganoderma lucidum*, *G. sinense*, and *G. resinaceum* have gained significant attentions for its bioactive compounds, including polysaccharides, triterpenoids, and phenolic acids [1–3]. These compounds exhibit diverse bioactivities, such as immunomodulation [4], anti-inflammatory effects [5], and ROS scavenging [6], making *Ganoderma* a candidate for functional food and medicine development. Besides, *Ganoderma* species has been used in cosmetics industry due to its safety and health benefits. Evidence from several studies demonstrates that *Ganoderma* extracts (GE) and polysaccharides (GP) confer antioxidant protection via the activation of antioxidant enzymes and the reduction of UVB-induced reactive oxygen species (ROS) [7]. Additionally, GP acts through the IL-6/STAT3 pathway to diminish melanogenesis in melanocytes, counteracting paracrine influences exerted by keratinocytes and fibroblasts [8]. Ganoderol A protects against UVA damage and exhibits anti-inflammatory activity [9]. The potential for *Ganoderma* triterpenoids (GTs), major active compounds in *Ganoderma* species, to confer anti-skin-aging benefits and the underlying mechanisms remain to be established.

The skin provides a visible model of the systemic aging process, reflecting contributions from both innate biological programs (intrinsic aging) and environmental exposures (extrinsic aging) [10–12]. A central mechanism of extrinsic aging, particularly photoaging, involves oxidative stress. Ultraviolet (UV) radiation triggers a damaging cycle by inducing overproduction of reactive oxygen species (ROS) in skin cells [11, 13]. This state of oxidative stress not only directly harms cellular components but also activates specific signaling pathways. It promotes the overexpression of matrix metalloproteinases (MMPs)—enzymes responsible for the degradation of the extracellular matrix (e.g., collagen, elastin)—largely through the activation of the MAPK pathways (ERK, JNK, p38) [14–16]. The downstream outcome of this signaling is increased activity of the transcription factor AP-1, leading to collagen breakdown and inflammation. Given this mechanism, targeting oxidative stress with antioxidants is a rational and crucial strategy for mitigating skin photoaging [17, 18].

A promising target for the prevention and treatment of oxidative stress-related chronic diseases is the Keap1-Nrf2 signaling pathway [19–21]. This pathway maintains cellular homeostasis by upregulating key protective proteins [22]. The transcription factor Nrf2 activates antioxidant response elements (ARE) in promoters, driving expression of detoxification and antioxidant genes. Keap1 directly regulates Nrf2 by sensing oxidative/chemical stress [22, 23]. As a result, enhancing Nrf2 activity is considered a potential strategy for preventing and treating various chronic diseases [20, 24, 25], including diabetes, cancer, and neurodegenerative disorders, which are linked to oxidative stress.

**Fig. 1:**
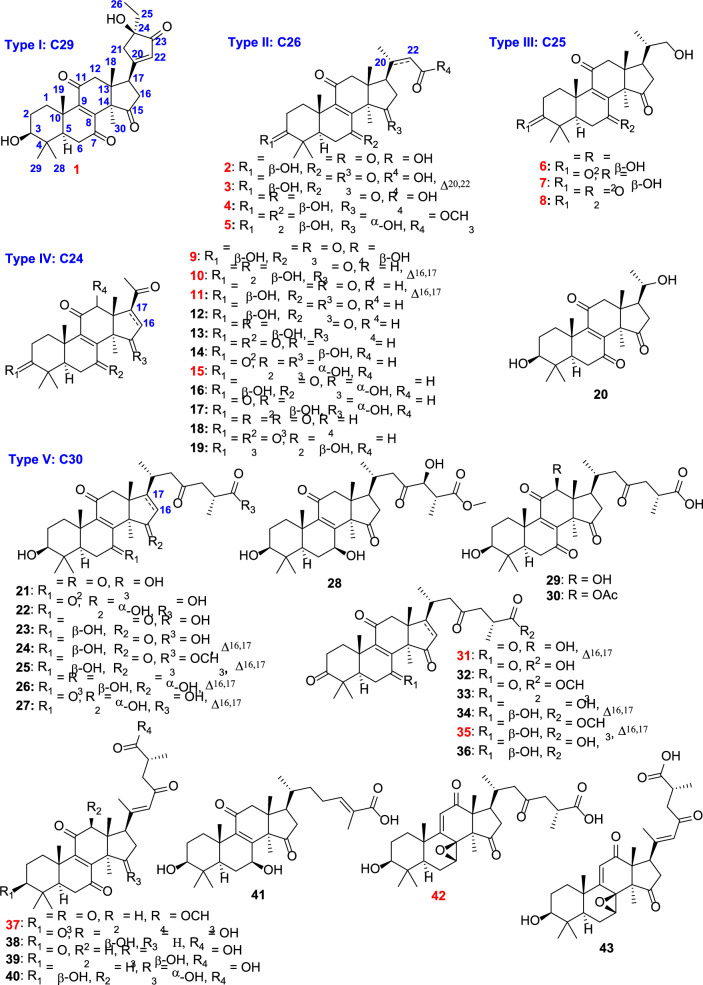
Structures of isolates from *G. resinaceum*. (red number: new compounds)

To investigate the anti-photoaging properties of GTs, we conducted a systematic study on the GTs from the fruiting bodies of *Ganoderma resinaceum*, a medicinal and edible mushroom [26, 27]. A total of 43 lanostane-type triterpenoids (**1**‒**43**) were isolated (Fig. 1), exhibiting various degrees of degradation, among which sixteen compounds (**1**‒**11**, **15**, **31**, **35**, **37**, and **42**) were novel. We assessed their anti-photoaging effects and mechanisms of action using a UV-induced HaCaT cell model. Our findings suggest that ganoresinic acid B (**42**) may serve as a promising candidate for anti-photoaging treatment, as it demonstrated significant antioxidative capabilities by activating Nrf2 nuclear translocation, and protection against collagen degradation through the suppression of the MAPK signaling pathway.

## Results and discussion

### Structural elucidation

The phytochemical investigation of the fruiting bodies of *G. resinaceum* was performed, leading to the isolation of 43 triterpenoids including 16 new compounds (**1**‒**11**, **15**, **31**, **35**, **37**, and **42**).

**Fig. 2:**
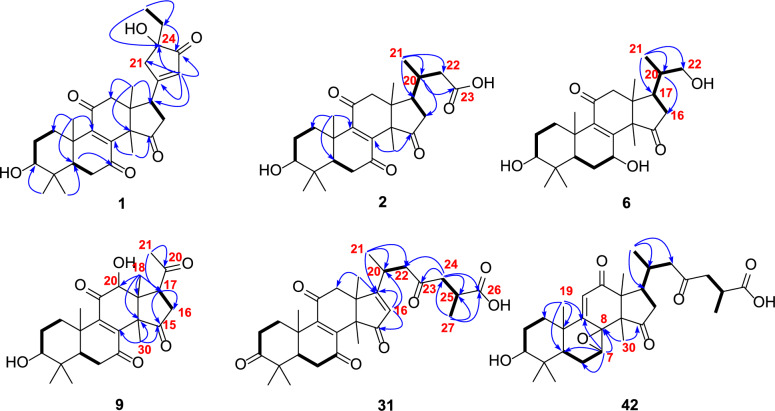
Selected HMBC (H → C) and ^1^H-^1^H COSY (─) correlations of compounds **1**, **2**, **6**, **9**, **31**, and **42**

Ganoresinol A (**1**) exhibited a molecular ion cluster at *m/z* 516.2364 [M + Cl]⁻ in negative HRESIMS, consistent with the molecular formula C₂₉H₃₈O₆ (calcd. for C₂₉H₃₈O₆Cl, 516.2362; Δ = 0.2 ppm). This formula implies 11 degrees of unsaturation. Key ^1^H NMR features included five methyl singlets (*δ*_H_ 0.76–1.62), a triplet methyl (*δ*_H_ 0.89, *J* = 2.3 Hz), a dd oxymethine proton (*δ*_H_ 3.20, *J* = 11.9, 5.3 Hz), and a singlet olefinic/aromatic methine (*δ*_H_ 6.06). Analysis of the ^13^C-DEPT NMR data for **1** confirmed 29 distinct carbons: six methyl groups, seven methylene groups, four methines (including an oxymethine and an olefinic/aromatic methine), and twelve quaternary carbons—comprising four carbonyls, three *sp*^2^ quaternary, one oxygenated, and four aliphatic quaternary carbons. Collectively, the NMR data support a 3-hydroxy-7,11,15-trioxo-lanosta-8-en skeleton, mirroring the structure of **21** [28]. The assignment was confirmed by HMBC correlations, including H₃-19 to C-9 (*δ*_C_ 153.6) and H₃-30 to C-15 (*δ*_C_ 208.8), while COSY spin systems (H₂-1/H₂-2/H-3) established proton-proton linkages (Fig. 2).

The core framework explained eight degrees of unsaturation, with the ketone and double bond contributing two more, necessitating an additional ring in **1**. This ring was identified as cyclopentenone through HMBC correlations (Fig. 2): from H-17 (*δ*_H_ 3.51, t, *J* = 8.9 Hz) to C-20 (*δ*_C_ 179.3), C-21 (*δ*_C_ 47.3), and C-22 (*δ*_C_ 129.3), and from H_2_-21 (*δ*_H_ 2.58, m; 2.80, m) and H-22 (*δ*_H_ 6.06, s) to C-17 (*δ*_C_ 45.0), C-20 (*δ*_C_ 179.3), C-23 (*δ*_C_ 211.5), and C-24 (*δ*_C_ 79.2). Additional support for the ethyl linkage at C-24 came from HMBC interactions: H₃-26 (*δ*_H_ 0.89, t, *J* = 2.5 Hz) with C-24 and H₂-25 with C-21/C-22. These observations finalized the planar structure assignment.

**Fig. 3:**
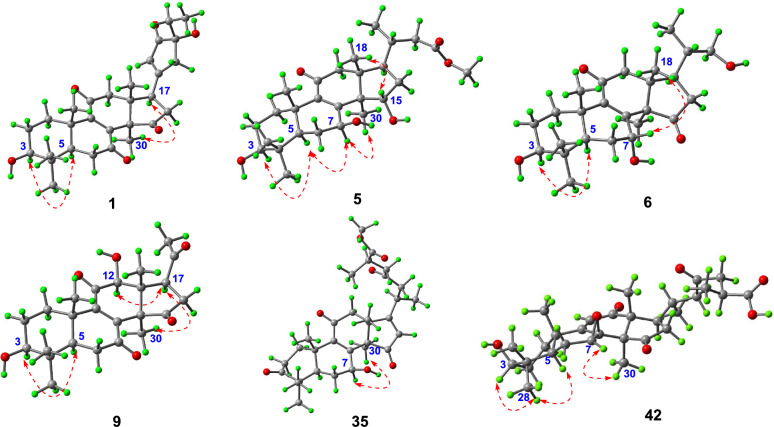
Key ROESY correlations of compounds **1**, **5**, **6**, **9**, **35**, and **42**

**Fig. 4:**
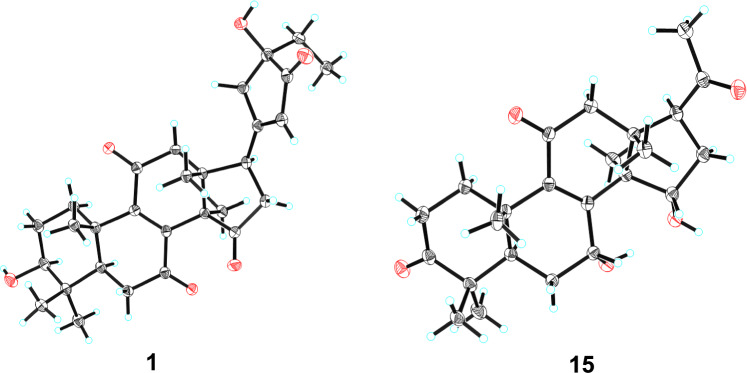
X-ray diffraction crystal structures of compounds **1** and **15**

Stereochemical features were resolved through complementary techniques: the *β*-orientation of 3-OH followed from the H-3/H-5 ROESY correlation (Fig. 3), while X-ray diffraction (*P*1211, Flack parameter 0.02; CCDC: 2,259,021, Fig. 4) defined C-24 as *R*-configuration. Ganoresinol A (1) thus emerges as 3*β*,24*R*-dihydroxy-27-nor-21,24-cyclolanosta-8,20(22)-dien-7,11,15,23-tetraone.

Ganoresic acid A (**2**) exhibited an [M + Cl]⁻ peak at *m/z* 481.2364 (calcd. 481.2362) by HRESIMS, corresponding to C₂₆H₃₆O₆. Key ^1^H NMR signals (Table 1) included five methyl singlets (*δ*_H_ 0.85, 0.85, 1.01, 1.29, 1.55), one methyl doublet (1.05, d, *J* = 6.3 Hz), and an oxymethine (3.20, dd, *J* = 11.6, 4.8 Hz). ^13^C/DEPT data confirmed 26 carbons: 6 × CH₃, 6 × CH₂, 4 × CH (including *δ*_C_ 78.1 oxymethine), and 10 × C (3 × ketone, 1 × carboxyl, 2 × *sp*^2^, 4 × aliphatic quaternary). These features characterize **2** as a tetranorlanostane triterpenoid.

**Table 1:** ^1^H (600 MHz) and ^13^C (150 MHz) NMR spectroscopic data of compounds **1**‒**5**. (*δ* in *ppm*, CD_3_OD)

Positions	1^*a*^		2^*a*^		3^*a*^		4^*a*^		5^*a*^	
	*δ*_H_	*δ*_C_	*δ*_H_	*δ*_C_	*δ*_H_	*δ*_C_	*δ*_H_	*δ*_C_	*δ*_H_	*δ*_C_
1	2.81, m	34.7, CH_2_	2.78, m; 2.01, m	34.5, CH_2_	2.81, m	34.8, CH_2_	1.01, m; 2.78, m	35.9 CH_2_	2.12, m	28.9, CH_2_
2	1.57, m; 1.62, m	31.4, CH_2_	1.68, m	28.0, CH_2_	1.12, m; 1.67, m	28.0, CH_2_	1.58, m	28.2 CH_2_	1.60, m	28.3, CH_2_
3	3.20, dd (11.9, 5.3)	78.2, CH	3.20, dd (11.6, 4.8)	78.1, CH	3.22, dd (11.6, 4.8)	78.2, CH	3.14, dd (11.8, 4.6)	78.9 CH	3.14, dd (11.8, 4.6)	78.8, CH
4		40.2, C		40.2, C		40.1, C		39.9 C		39.7, C
5	1.62, m	52.1, CH	1.59, m	52.4, CH	1.63, m	51.9, CH	0.92, m	50.3 CH	0.93, m	50.3, CH
6	2.52, m; 2.67, m	37.1, CH_2_	2.49, dd (14.8, 4.9); 2.66, dd (14.8, 3.0)	37.3, CH_2_	2.52, m; 2.64, m	37.1, CH_2_	2.17, m	28.6 CH_2_	0.93, m; 2.68, m	35.8, CH_2_
7		201.4, C		201.8, C		201.3, C	4.83, t (7.5)	67.8 CH	4.51, dt (10.2, 7.4)	70.1, CH
8		147.5, C		147.8, C		148.3, C		158.9 C		161.2, C
9		153.6, C		153.4, C		153.3, C		144.0 C		143.1, C
10		41.8, C		41.7, C		41.7, C		39.6 C		39.7, C
11		200.2, C		201.3, C		200.7, C		200.3 C		202.3, C
12	2.58, m; 3.19, m	48.8, CH_2_	2.66, d (15.7); 3.04, d (15.7)	50.4, CH_2_	2.53, d (15.7); 3.12, d (15.7)	49.4, CH_2_	2.92, d (16.7); 2.62, d (16.7)	51.4 CH_2_	2.38, d (15.2); 2.86, d (15.2)	53.1, CH_2_
13		46.4, C		45.5, C		46.7, C		46.6 C		48.3, C
14		57.9, C		58.5, C		57.7, C		60.3 C		55.3, C
15		208.8, C		210.6, C		209.8, C		218.2 C	4.77, d (8.1)	73.1, CH
16	2.59, m; 2.87, m	38.0, CH_2_	1.93, dd (18.2, 8.7); 2.83, overlapped	41.1, CH_2_	2.53 overlapped; 2.66, overlapped	37.8, CH_2_	2.17, m; 2.76, m	41.7 CH_2_	1.71, m	36.9, CH_2_
17	3.51, t (8.9)	45.0, CH	2.29, m	46.0, CH	3.34, s	49.0, CH	2.17 overlapped	46.8 CH	1.85, m	49.1, CH
18	0.76, s	18.2, CH_3_	0.85, s	16.1, CH_3_	0.72, s	18.2, CH_3_	0.84, s	16.1 CH_3_	0.95, s	17.3, CH_3_
19	1.28, s	18.0, CH_3_	1.29, s	18.1, CH_3_	1.25, s	17.9, CH_3_	1.20, s	18.7 CH_3_	1.25, s	19.9, CH_3_
20		179.3, C	2.02, m	34.5, CH		156.7, C	2.05, m	34.4 CH	1.90, m	35.2, CH
21	2.58, m; 2.80, m	47.3, CH_2_	1.05, d (6.3)	19.7, CH_3_	2.18, s	20.7, CH_3_	1.05, s	19.8 CH_3_	0.92, d (6.0)	19.8, CH_3_
22	6.06, s	129.3, CH	2.09, dd (15.3, 9.3); 2.37, dd (15.3, 3.4)	41.8, CH_2_	5.75, s	119.2, CH	2.35, m; 2.06, m	42.0 CH_2_	2.42, dd (14.9, 3.2); 2.07, m	42.0, CH_2_
23		211.5, C		176.4, C		169.6, C		176.7 C		175.3, C
24		79.2, C								
25	1.56, m; 1.68, m	28.0, CH_2_								
26	0.89, t, (2.3)	8.07, CH_3_								
27										
28	1.01, s	28.3, CH_3_	1.01, s	28.3, CH_3_	1.01, s	28.2, CH_3_	1.02, s	28.6, CH_3_	1.01, s	28.6, CH_3_
29	0.87, s	16.0, CH_3_	0.85, s	16.5, CH_3_	0.87, s	16.0, CH_3_	1.00, s	17.8, CH_3_	0.83, s	16.3, CH_3_
30	1.62, s	22.1, CH_3_	1.55, s	21.8, CH_3_	1.57, s	22.6, CH_3_	1.37, s	24.8, CH_3_	1.22, s	19.8, CH_3_
OCH_3_									3.65, s	51.9, CH_3_

HMBC data confirmed the tetracyclic 3-hydroxy-7,11,15-trioxo scaffold in **2** (Fig. 2). Key observations included correlations from multiple protons to the carbonyl and oxygen-bearing carbons. The degraded side chain (C-24–C-27) was established by HMBC (H₃-21 → C-17/C-20/C-22) and the COSY correlations H₂-16/H-17/H-20/H₂-22. The ROESY correlation (H-3/H₃-28/H-5) defined the 3*β*-OH orientation. The structure of **2** is therefore 3*β*-hydroxy-24,25,26,27-tetranorlanosta-8-en-7,11,15-trioxo-23-oic acid.

HRESIMS analysis of ganoresinol B (**6**) showed a deprotonated molecular ion [M–H]⁻ at *m/z* 417.2646 (calculated 417.2649), corresponding to the molecular formula C₂₅H₃₈O₅. Key 1D NMR data (Table 2) of **6** displayed 6 × CH_3_, 6 × CH_2_ (one oxygenated), 5 × CH (two oxygenated), and 8 × C (two ketones and two *sp*^2^ C). This pentanorlanostane triterpenoid framework resembles ganosineniol A [29], differing solely through carbonyl substitution at C-15 (replacing the oxygenated methine). The further HMBC correlations (Fig. 2) from H-17 and H_3_-30 to C-15 supported this modification. Additionally, ROESY cross-peaks (H-3/H-5/H-7/H₃-30) (Fig. 3) confirmed the *β*-orientation of both the 3-OH and 7-OH groups. Compound **6** is therefore assigned as 3*β*,7*β*,22-trihydroxy-23,24,25,26,27-pentanorlanosta-8,9-en-11,15-dione.

**Table 2:** ^1^H (600 MHz) and ^13^C (150 MHz) NMR spectroscopic data of compounds **6**‒**10**. (*δ* in *ppm*)

Positions	6^*a*^		7^*a*^		8^*a*^		9^*b*^		10^*b*^	
	*δ*_H_	*δ*_C_	*δ*_H_	*δ*_C_	*δ*_H_	*δ*_C_	*δ*_H_	*δ*_C_	*δ*_H_	*δ*_C_
1	1.01, m; 2.78, m	35.7, CH_2_	2.78, m; 2.01, m	36.7, CH_2_	1.81, m; 2.78, m	35.5, CH_2_	1.12, m; 2.73, m	33.4, CH_2_	1.07, m; 2.95, m	34.5, CH_2_
2	2.17, m; 1.56, m	27.9, CH_2_	2.48, m	34.7, CH_2_	2.37, m	34.7, CH_2_	1.72, overlapped	27.3, CH_2_	1.67, m	27.3, CH_2_
3	3.14, dd (11.8, 4.4)	78.8, CH		219.7, C	3.22, dd (11.6, 4.8)	218.3, C	3.28, dd (11.5, 4.9)	77.5, CH	3.25, dd (11.6, 4.8)	78.3, CH
4		39.7, C		47.7, C		48.1, C		39.0, C		38.6, C
5	0.94, m	50.3, CH	1.74, m	49.0, CH	2.40, m	51.7, CH	1.54, dd (14.7, 2.2)	51.3, CH	0.94, m	49.2, CH
6	1.59, m	28.2, CH_2_	1.60, m; 2.14, m	28.9, CH_2_	2.40, m; 2.75, m	37.9, CH_2_	2.57, m; 2.67, m	36.5, CH_2_	0.93, m; 2.68, m	26.3, CH_2_
7	4.83, m	67.8, CH	4.90, dd (9.4, 7.6)	67.2, CH		201.6, C		198.4, C	4.85, dd (10.0, 7.6)	67.1, CH
8		158.9, C		159.9, C		147.7, C		146.2, C		156.1, C
9		143.9, C		142.3, C		151.4, C		151.0, C		142.7, C
10		39.6, C		39.2, C		40.4, C		40.2, C		39.7, C
11		200.2, C		200.1, C		201.6, C		200.3, C		197.7, C
12	2.58, m; 3.19, m	48.8, CH_2_	2.65, d (17.5); 2.90, d (17.5)	51.2, CH_2_	2.70, d (16.2); 3.05, d (16.2)	49.7, CH_2_	4.69, s	76.8, CH	2.94, d (17.4); 3.07, d (17.4)	44.7, CH_2_
13		46.4, C		46.1, C		45.0, C		49.7, C		49.2, C
14		60.0, C		60.0, C		58.3, C		57.5, C		59.0, C
15		218.8, C		218.7, C		210.5, C		204.1, C		211.0, C
16	2.17, m; 1.56, m	41.5, CH_2_	2.24, m; 2.76, m	41.6, CH_2_	1.90, m; 2.85, overlapped	40.5, CH_2_	2.56, m; 2.73, m	35.5, CH_2_	6.47, s	133.8, CH
17	2.23, m	43.6, CH	2.26, m	43.6, CH	2.36, s	42.4, CH	3.72, m	50.3, CH		169.1, C
18	0.98, s	17.9, CH_3_	1.03, s	17.2, CH_3_	0.86, s	16.3, CH_3_	0.57, s	11.0, CH_3_	1.28, s	31.2, CH_3_
19	1.20, s	18.7, CH_3_	1.25, s	18.0, CH_3_	1.28, s	19.0, CH_3_	1.36, s	17.8, CH_3_	1.18, s	18.2, CH_3_
20	1.64, m	39.4, CH	1.69, m	39.5, CH	1.60, m	39.6, CH		207.8, C		196.4, C
21	1.06, d (6.6)	17.3, CH_2_	1.09, d (6.6)	18.0, CH_3_	1.05, d (6.0)	19.0, CH_3_	2.44, s	31.9, CH_3_	2.47, s	28.3, CH_3_
22	3.38, dd (11.0, 5.7); 3.52, dd (11.0, 3.3)	67.4, CH_2_	3.40, dd (11.0, 5.7); 3.53, dd (11.0, 3.3)	67.4, CH_2_	3.37, dd (11.0, 5.8); 3.51, dd (11.0, 3.3)	67.3, CH				
28	1.02, s	28.5, CH_3_	1.15, s	27.3, CH_3_	1.12, s	27.6, CH_3_	1.03, s	27.7, CH_3_	1.05, s	28.1, CH_3_
29	0.83, s	16.1, CH_3_	1.12, s	21.0, CH_3_	1.10, s	20.6, CH_3_	0.89, s	15.5, CH_3_	0.85, s	15.5, CH_3_
30	1.36, s	24.7, CH_3_	1.40, s	25.1, CH_3_	1.66, s	20.9, CH_3_	1.73, s	20.2, CH_3_	1.54, s	32.3, CH_3_

^*a*^: CD_3_OD; ^*b*^: CDCl_3_

Compound **9** shared a core structure with ganolucidoid B (**32**) [5] but lacked its C-12 acetyl group. This conclusion was underpinned by HMBC data (Fig. 2): the correlation from H-12 to C-9, among others, confirmed the substitution at C-12, while the H₃-18 to C-12 interaction was also consistent. The *β*-orientations of the 3-OH and 12-OH groups were concurrently verified by ROESY correlations (H-3/H-5 and H-12/H₃-30) (Fig. 3). Accordingly, the structure of **9** was defined as 3β,12β-dihydroxy-22,23,24,25,26,27-hexanorlanosta-8(9)-en-7,11,15,20-tetraone, for which we propose the name ganoresinone A.

HRESIMS analysis of **15** showed an [M + Na]⁺ ion at *m/z* 421.1988 (calcd. for C₂₄H₃₀O₅Na: 421.1985), confirming the molecular formula C₂₄H₃₀O₅, identical to compound **17** [30]. Comparative ^1^H/^13^C NMR analysis revealed distinct chemical shifts at C-7 (*δ*_C_ 69.0 for **17**; *δ*_C_ 67.8 for **15**), suggesting differential stereochemistry. The ROESY spectrum of **15** showed that correlations of H-3/H-5, and of H-7/H-15/H_3_-18 illustrated that 3-OH was *β*-oriented, whereas both 7-OH and 15-OH were *α*. Complementary X-ray crystallography (*P*1211, Flack parameter = 0.02, CCDC: 2,259,022, Fig. 4) unequivocally confirmed both planar structure and stereochemical assignments. Thus, **15** is characterized as 3*β*,7*α*,15*α*-trihydroxy-22,23,24,25,26,27-hexanorlanosta-8(9)-en-11,20-dione and assigned the trivial name ganoresinone D.

RESIMS data for ganoresinic acid A (**31**) displayed a sodium adduct peak [M + Na]⁺ at *m/z* 533.2517 (calculated 533.2510 for C₃₀H₃₈O₇Na), confirming its molecular formula. The ^1^H and ^13^C NMR data of **31** were consistent with a C₃₀ lanostane-type triterpenoid closely related to ganoderic acid E [31] but featuring a Δ^1^⁶⁽^1^⁷⁾ double bond instead. Key HMBC correlations from H-16 and H₃-18 established the position of this double bond. The R configuration at C-25 was deduced from the identical chemical shifts of C-24–C-27 to those in resinacein T [26]. These data collectively determine the structure of 31 to be 3,7,11,15,23-pentaoxo-lanosta-8(9),16(17)-dien-26-oic acid, designated ganoresinic acid A.

Compound **37** was identified as methyl 3,7,11,15,23-pentaoxo-lanosta-8(9),20(22)-dien-26-oate, a methyl ester derivative of methyl ganoderate E (**33**) [5]. This assignment was based on NMR data that indicated the sole difference was an added methoxy group, which was shown by HMBC to be ester-linked to C-26. The *E*-configuration of the Δ^2^⁰^,22^ double bond was confirmed by ROESY data, leading to the name methyl ganoderenate F.

HRESIMS analysis established the molecular formula of ganoresinic acid B (**42**) as C₃₀H₄₂O₇ ([M + Na]⁺ at *m/z* 539.2972, calcd. 539.2979). Its 1D NMR data (Table 3) closely matched those of **41** and **36** [32], sharing the 7,8-epoxy-9(11)-en-12-one core but differing by a C-20/C-22 double bond. The side-chain structure was defined by key HMBC and COSY correlations (Fig. 2). ROESY data (Fig. 3) established the *β*-orientations of the 3-OH and 7,8-epoxy groups, while the R configuration at C-25 was confirmed by comparison with resinacein T [26]. The structure was thus determined to be 3*β*-hydroxy-7β,8β-epoxy-12,15,23-trioxo-lanosta-9(11)-en-27-oic acid.

**Table 3:** ^1^H (600 MHz) and ^13^C (150 MHz) NMR spectroscopic data of compounds **11**, **15**, **31**, **35**, **37**, and **42**. (*δ* in *ppm*)

Positions	11^*b*^		15^*a*^		31^*a*^		35^*b*^		37^*b*^		42^*a*^	
	*δ*_H_	*δ*_C_	*δ*_H_	*δ*_C_	*δ*_H_	*δ*_C_	*δ*_H_	*δ*_C_	*δ*_H_	*δ*_C_	*δ*_H_	*δ*_C_
1	1.49, m; 2.92, m	33.8, CH_2_	1.69, m; 2.90, m	35.9, CH_2_	1.88, m; 2.99, m	35.9, CH_2_	2.78, m; 2.01, m	36.7, CH_2_	2.78, m; 2.01, m	36.7, CH_2_	1.48, m; 1.89, m	37.9, CH_2_
2	1.70, m; 1.78, m	27.2, CH_2_	2.39, m	35.0, CH_2_	1.57, m; 1.62, m	31.4, CH_2_	2.48, m	34.7, CH_2_	2.48, m	34.7, CH_2_	1.69, m	27.7, CH_2_
3	3.32, dd (11.7, 4.4)	77.6, CH		220.7, C		218.1, C		216.7, C		215.0, C	3.14, dd (10.1, 5.8)	78.7, CH
4		38.7, C		47.6, C		40.2, C		47.2, C		46.9, C		39.1, C
5	1.74, m	48.8, CH	1.74, m	48.8, CH	2.32, dd (15.0, 2.8)	50.7, CH	1.69, m	48.6, CH	1.69, m	48.6, CH	1.15 overlapped	49.1, CH
6	2.52, d (4.2); 2.70, dd (18.5, 4.2)	34.7, CH_2_	1.78, m	28.5, CH_2_	2.51, m; 2.63, m	34.8, CH_2_	1.65, m; 2.10, m	27.1, CH_2_	2.50, m; 2.69, m	36.9, CH_2_	1.95, m; 2.17, m	22.0, CH_2_
7		200.2, C	4.61 overlapped	67.1, CH		200.3, C	4.90, dd (9.4, 7.6)	66.4, CH		198.8, C	4.30, d (6.4)	58.7, CH
8		153.0, C		161.6, C		150.5, C		158.2, C		146.4, C		61.1, C
9		149.0, C		140.7, C		152.1, C		140.7, C		150.1, C		164.7, C
10		40.6, C		39.1, C		40.8, C		38.4, C		39.4, C		40.3, C
11		197.7, C		200.3, C		200.0, C		197.1, C		198.5, C	5.92, s	126.3, CH
12	3.00, m	45.3, CH_2_	3.11, d (17.4); 2.49, d (17.4)	51.4, CH_2_	2.68, (16.5); 3.17, d (16.5)	44.9, CH_2_	2.61, d (16.4); 2.95, d (16.4)	43.9, CH_2_	2.64, d (16.4); 2.95, d (16.4)	47.6, CH_2_		204.3, C
13		49.0, C		48.3, C		52.2, C		51.1, C		44.9, C		40.3, C
14		55.9, C		54.5, C		57.5, C		58.2, C		56.4, C		56.5, C
15		203.4, C	4.61 overlapped	72.7, CH		205.6, C		210.7, C		206.0, C		212.3, C
16	6.50, s	134.9, CH	2.80, m	31.5, CH_2_	5.70, s	123.7, CH	5.66, s	122.4, CH	2.68, m; 2.49, m	36.1, CH_2_	1.96, m; 2.64, m	41.6, CH_2_
17		166.1, C	3.37, dd (10.5, 7.3)	57.8, C		185.1, C		187.6, C	3.20, t (9.1)	48.3, CH	2.37, m	40.4, CH
18	1.12, s	29.9, CH_3_	0.75, s	19.2, CH_3_	1.13, s	19.4, CH_3_	1.25, s	30.9, CH_3_	0.74, s	17.6, CH_3_	1.31, s	16.9, CH_3_
19	1.12, s	17.4, CH_3_	1.03, s	20.7, CH_3_	1.26, s	17.5, CH_3_	1.22, s	18.4, CH_3_	1.27, s	18.4, CH_3_	1.18, s	22.4, CH_3_
20		196.8, C		210.2, C	1.12, m	30.7, CH	3.04, m	28.6, CH		153.3, C	2.04, m	33.3, CH
21	2.46, s	28.0, CH_3_	2.10, s	31.0, CH_3_	1.12 overlapped	19.9, CH_3_	1.13, d (6.6)	19.5, CH_3_	2.14, s	21.1, CH_3_	0.98, d (6.6)	20.6, CH_3_
22					2.75, m; 2.90, m	48.2, CH_2_	3.40, dd (11.0, 5.7); 3.53, dd (11.0, 3.3)	67.4, CH_2_	6.05, s	124.7, CH	2.51, m; 2.93, m	47.6, CH_2_
23						208.6, C		210.7, C		197.8, C		211.2, C
24					2.54, m; 2.87, m	44.9, CH_2_	2.40, m; 2.91, m	46.3, CH_2_	2.52, m; 2.63, m	47.7, CH_2_	2.36, m; 2.49, m	50.1, CH_2_
25					2.87, m	35.9, CH	2.96, m	34.6, CH	2.96, m	34.7, CH	2.83, m	36.3, CH
26					1.14, d (6.8)	17.4, CH_3_	1.18, d (7.2)	17.0, CH_3_	1.20, d (6.5)	17.2, CH_3_	1.15, d (6.6)	17.6, CH_3_
27						179.6, C		176.0, C		176.4, C		180.2, C
28	1.04, s	27.4, CH_3_	1.14, s	27.7, CH_3_	1.14, overlapped	27.0, CH_3_	1.14, s	27.4, CH_3_	1.14, s	27.3, CH_3_	1.00, s	28.3, CH_3_
29	0.90, s	14.9, CH_3_	1.06, s	20.7, CH_3_	1.14, overlapped	19.7, CH_3_	1.11, s	20.5, CH_3_	1.12, s	20.3, CH_3_	0.84, s	15.6, CH_3_
30	1.45, s	31.9, CH_3_	1.36, s	21.6, CH_3_	1.59, s	32.0, CH_3_	1.53, s	33.2, CH_3_	1.69, s	21.2, CH_3_	1.09, s	19.7, CH_3_
OCH_3_							3.66, s	51.9, CH_3_	3.69, s	51.9, CH_3_		

^*a*^: CD_3_OD; ^*b*^: CDCl_3_

Details of the structural characterization for the other new compounds can be found in the supplementary file. According to the number of carbons, these isolates can be divided into five subtypes, including C29 (Type I: compound **1**), C26 (Type II: compounds **2**‒**5**), C25 (Type III: compounds **6**‒**8**), C24 (Type IV: compounds **9**‒**20**), and C30 (Type VI: compounds **21**‒**43**). Compound **1** was the first example of the C29 lanostane-type nortriterpenoid with a rare 21,24-cyclo-five-membered carbon fraction. *Ganoderma* triterpenoids are belonging to lanostane-type triterpenoids, which were biosynthesized by isoprenoid pathways, starting from acetyl-coenzyme A [33]. Initially, the C30 skeleton was constructed, and subsequently, various nortriterpenoids can be generated through oxidative cleavage of the side chain (Scheme S1) [33].

### Pharmacology

#### Selection of irradiation dose in HaCaT cells

**Fig. 5:**
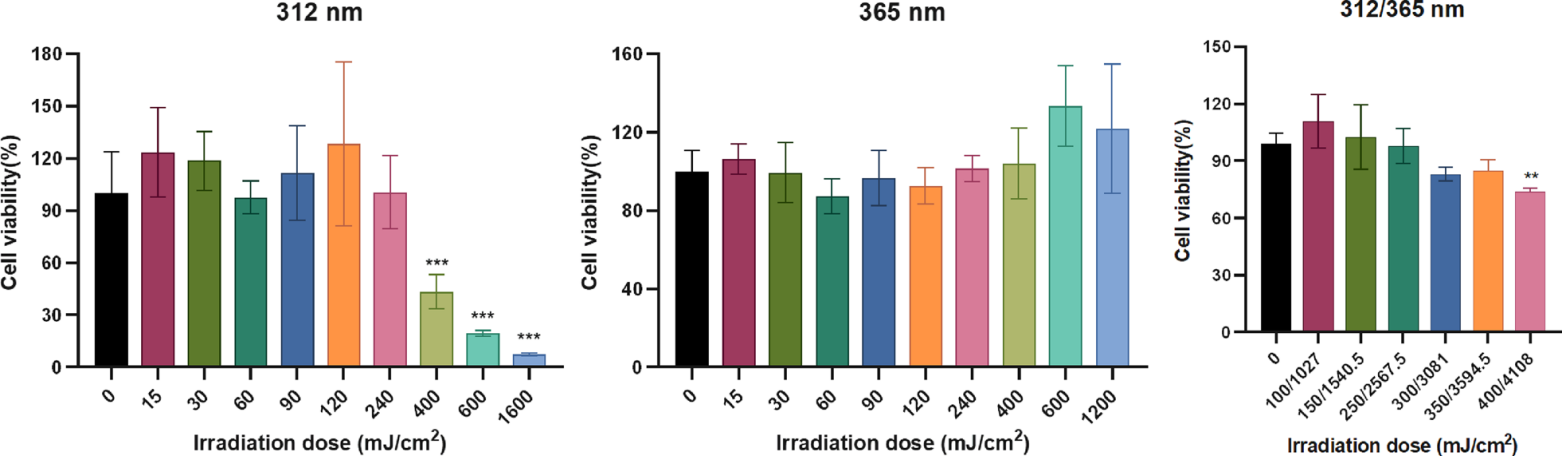
Effects of different doses of UV on cell viability for 24 h in HaCaT cells. One-way analysis of variance (ANOVA) with *p* < 0.05 versus 0 mJ/cm^2^ being considered statistical significance. Data are presented as the mean ± SD (n = 3)

Prior to assessing anti-photoaging compounds from *G. resinaceum* in HaCaT cells, we determined the appropriate UVA (365 nm) and UVB (312 nm) irradiation doses for model development. Viability assessment (Fig. 5) indicated that doses below 350/3594.0 mJ/cm^2^ maintained viability > 80%. Therefore, a dose of 250/2567.5 mJ/cm^2^ was utilized to create the photoaging model.

#### *Ganoderma* triterpenoids prevent UV-induced oxidative stress in HaCaT cells

Given the limited content of certain compounds, the anti-photoaging potential of compounds **2**–**14**, **16**–**25**, **27**–**31**, **34**–**37**, and **41**–**43** was investigated. First, the cytotoxicity of these compounds on HaCaT cells with or without UV irradiation was tested, and none exhibited toxicity at concentrations below 25 μM (Fig. S117). As a result, a concentration of 25 μM was chosen for the follow-up experiments.

**Fig. 6:**
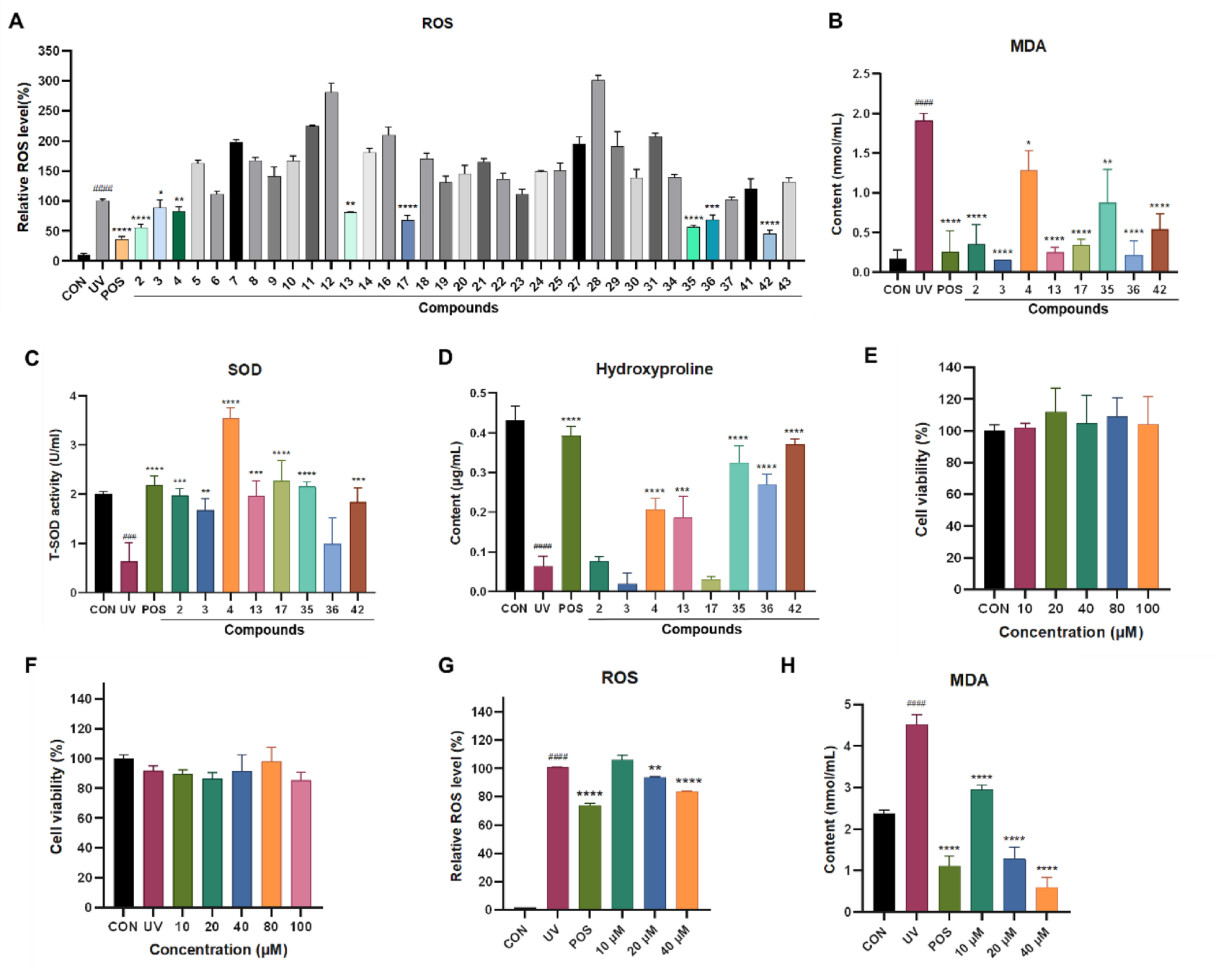
Evaluation of the antioxidant capacity of *Ganoderma* triterpenoids in UV-radiated HaCaT cells. **A** ROS levels in HaCaT cells treated with positive and compounds (25 μM). **B** MDA content in the culture medium of HaCaT cells treated with positive and compounds (25 μM). **C** SOD activity in the culture medium of HaCaT cells treated with positive and compounds (25 μM). **D** The hydroxyproline content in the culture medium of HaCaT cells treated with positive and compounds (25 μM). **E**, **F** The effect of **42** on HaCaT cell viability for 24 h without or with UV exposure. **G** Relative intracellular ROS level in HaCaT cells treated with or without different concentrations of **42** or positive, with or without UV. **H** The content of MDA in HaCaT cells treated with or without different concentrations of **42** or positive control, with or without UV. Data are presented as mean ± SD (n = 3). One-way analysis of variance (ANOVA) with ^###^*p* < 0.005, ^####^*p* < 0.001 versus CON, *p < 0.05, **p < 0.01, ***p < 0.005, ****p < 0.001 versus UV. CON: control, UV: model group, POS: positive control (resveratrol, 10 μM)

In skin cells, exposure to UV radiation triggers the generation of reactive oxygen species (ROS), resulting in DNA damage and the formation of senescent cells due to oxidative stress [10]. Malondialdehyde (MDA), a key byproduct of lipid peroxidation, is primarily formed through the degradation of polyunsaturated fatty acids in cell membranes when attacked by ROS and free radicals. The measurement of MDA levels is widely utilized as an indicator of oxidative stress intensity. As a critical component of the primary antioxidant defense mechanism, superoxide dismutase (SOD) was important in preserving the cellular redox balance [10]. Consequently, in our research, we assessed the impact of isolates on ROS levels, MDA production, and SOD activity. Our results (Fig. 6) showed the elevation of the intracellular ROS content in UV-irradiated HaCaT cells, whereas the significant decrease after pretreatment with compounds **2**‒**4**, **13**, **17**, **35**, **36**, and **42** at the concentration of 25 μM. Meanwhile, these active compounds also significantly suppressed the production of MDA. The activity of SOD was dramatically reduced by UV irradiation, but compounds **2**‒**4**, **13**, **17**, **35**, and **42** significantly reversed this tendency.

As we know, UV irradiation causes a reduction in collagen levels within the skin. Hydroxyproline (HYP), the most abundant amino acid in collagen, serves as an indicator of total collagen content [34]. Therefore, we also assessed the effects of various compounds on HYP levels. Our results showed that the HYP content within HaCaT cells declined following UV exposure. However, the HYP levels increased in the groups treated with compounds **4**, **13**, **35**, **36**, and **42** (Fig. 6). Based on the comprehensive analysis of the above indicators, Compound **42** is considered the most significantly active compound. Therefore, further activity studies were conducted on compound **42**.

#### Protective effect of compound 42 against UV-induced oxidative stress in HaCaT cells

The cytotoxic effects of compound **42** on both normal and UV-induced HaCaT cells were examined. The results revealed that compound **42** exhibited no cytotoxic effect on HaCaT cells with or without UV irradiation, even at a high concentration of 100 μM. Compound **42** dose-dependently reduced ROS and MDA levels. At 40 μM, its inhibition matched the positive control resveratrol (10 μM) (Fig. 6).

#### Compound 42 protects against UV-induced photoaging by inhibiting MAPK signaling

**Fig. 7:**
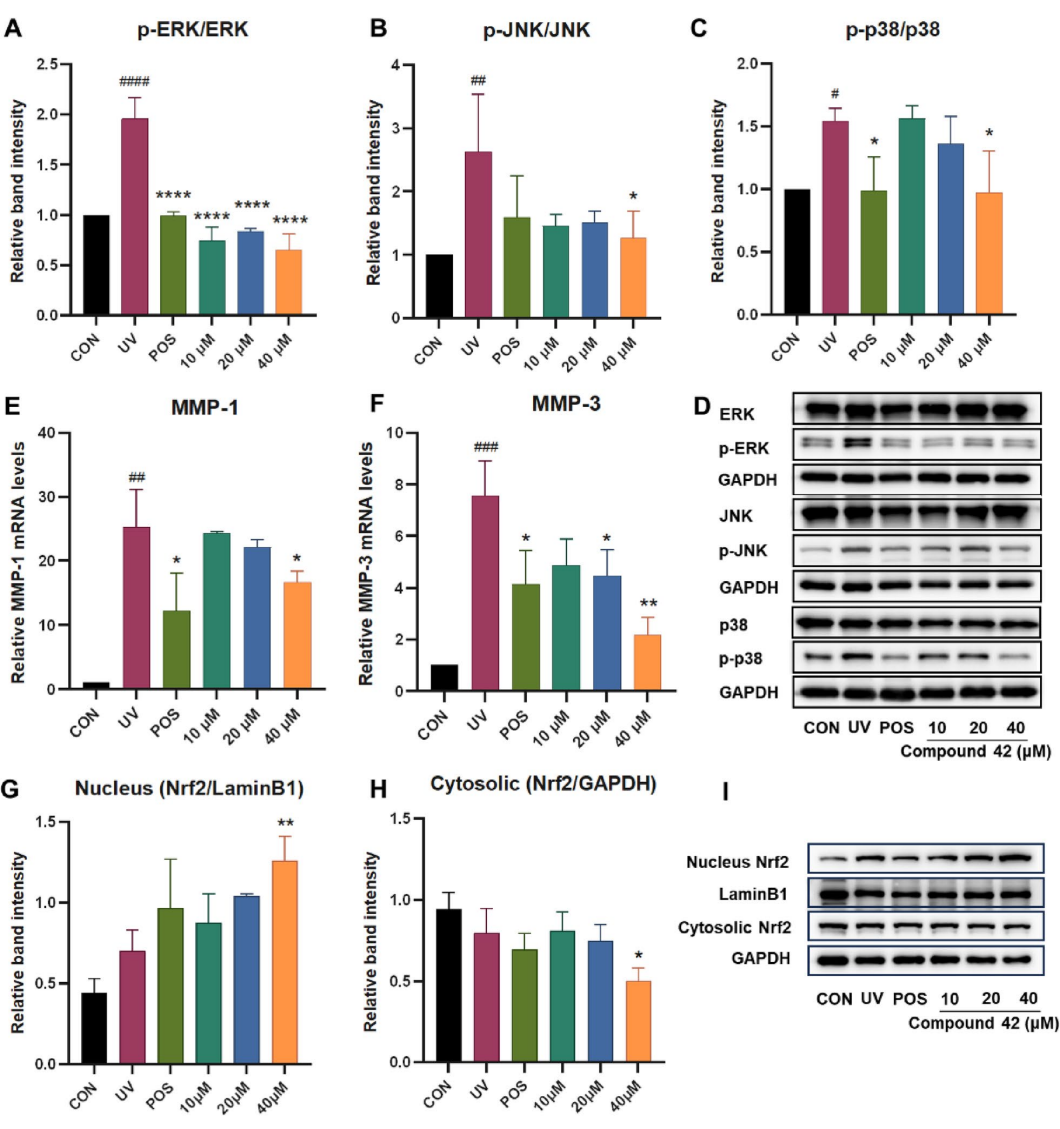
Effects of different concentrations of **42** on the MMPs, MAPK signaling pathway and Nrf2 expression in HaCaT cells under UV. **A**, **B**, **C**) Quantitative analysis of p-ERK/ERK, p-JNK/JNK, and p-p38/p38 in HaCaT cells. **D** Western blot image of p-ERK/ERK, p-JNK/JNK, and p-p38/p38 in HaCaT cells. **E**, **F** Quantitative results of relative MMPs mRNA levels in HaCaT cells. **G** Quantitative analysis of Nrf2 in HaCaT cell nucleus. **H** Quantitative analysis of Nrf2 in HaCaT cell cytoplasm. **I** Western blot image of Nrf2 in HaCaT cell nucleus and cytoplasm. One-way analysis of variance (ANOVA) with ^####^*p* < 0.001 versus CON, *p < 0.05, **p < 0.01, ***p < 0.005, ****p < 0.001 versus UV. CON: blank control, UV: model group, POS: positive control (resveratrol). Data are presented as the mean ± SD (n = 3)

Under UV exposure, reactive oxygen species (ROS) accumulate, prompting the activation of key MAPK signaling proteins—ERK, p38, and JNK. This cascade further stimulates AP-1, leading to the upregulation of matrix metalloproteinases (MMPs). The increased MMP activity, in turn, accelerates the degradation of collagen and other extracellular matrix (ECM) proteins—a key process in skin aging [16]. As shown in Fig. 7, UV-treated cells exhibited markedly higher levels of phosphorylated ERK, JNK, and p38 compared to untreated controls. Notably, compound **42** at 40 μM demonstrated the strongest inhibitory effect, substantially reducing the phosphorylation of all three MAPK proteins. Furthermore, compound **42** suppressed the mRNA expression of MMP-1 and MMP-3 in a concentration-dependent manner. By attenuating MAPK activation, compound **42** led to reduced MMP expression and consequently inhibited ECM degradation, suggesting a potential mechanism for its mitigation of UV-induced photoaging.

#### Compound 42 protects against UV-induced oxidative stress via activating NRF2

Nrf2 functions as a central regulator within the KEAP1/NRF2 signaling pathway. Under basal conditions, KEAP1 constitutively suppresses NRF2 activity by facilitating its ubiquitination and subsequent proteasomal degradation. Electrophilic stimuli, however, interfere with KEAP1’s inhibitory control over NRF2, enabling its stabilization and translocation to the nucleus. In the nucleus, NRF2 functions as a transcription factor to activate cytoprotective antioxidant genes such as superoxide dismutase (SOD) [23]. Thus, NRF2 has been considered as an important pharmacological target. To investigate the effect of compound **42** on NRF2 activation, we assessed its subcellular localization in HaCaT cells by Western blotting. Treatment with 40 μM compound 42 increased NRF2 levels in the nucleus while decreasing them in the cytoplasm compared to the model group (Fig. 7), indicating its translocation. Together with the observed suppression of MDA and activation of SOD, these results suggest that compound **42** mitigates oxidative stress by promoting NRF2 nuclear translocation, thereby activating antioxidant defense systems to neutralize ROS.

## Conclusion

In summary, this study reports the isolation of forty-three lanostane-type triterpenoids with varying degrees of degradation, which included C30, C29, C26, C25, and C24 triterpenoids from the fruiting bodies of *G. resinaceum*. Notably, sixteen of these triterpenoids were identified as new compounds, significantly expanding the structural diversity of *Ganoderma* triterpenoids. Additionally, in line with the known anti-aging effects of *Ganoderma* species, we assessed the anti-photoaging activities of compounds **2**‒**14**, **16**‒**25**, **27**‒**31**, **34**‒**37**, and **41**‒**43**. Among them, compound **42** significantly alleviated UV-induced oxidative stress in HaCaT cells by reducing ROS and MDA levels while enhancing SOD activity and hydroxyproline content. Mechanistic studies revealed that these protective effects are mediated through the promotion of Nrf2 nuclear translocation and the concurrent suppression of the MAPK signaling pathway, as evidenced by reduced phosphorylation of ERK, JNK, and p38. Consequently, the downstream mRNA expression of collagen-degrading enzymes MMP-1 and MMP-3 was downregulated. Collectively, our findings indicate that compound **42** exerts anti-photoaging effects by activating the Nrf2-antioxidant axis and inhibiting the MAPK-MMPs pathway. This work not only expands the structural diversity of *Ganoderma* triterpenoids (GTs) but also provides scientific evidence for the anti-aging potential of *Ganoderma* species.

## Materials and methods

### General information

Refer to the Supplementary materials for details regarding the instruments and reagents employed in isolation and structural characterization.

### Fungi material

The fruiting bodies of *Ganoderma resinaceum* (10 kg) were collected from Kunming’s Luoshiwan Traditional Chinese Medicine Market (June 2016). Authentication was conducted by Prof. Yang Zhuliang (Kunming Institute of Botany, CAS), with a voucher specimen (KGR-201606) archived for reference.

### Extraction and isolation

10 kg of powdered, air-dried *G. resinaceum* fruiting bodies were exhaustively extracted via reflux with methanol (3 × 50 L). The resultant extract was aqueous-suspended and subjected to sequential solvent extraction with petroleum ether followed by ethyl acetate. The ethyl acetate-soluble material (300 g) was purified by D101 macroporous resin chromatography employing a stepped methanol/water gradient (0% → 20% → 50% → 70% → 90% MeOH). The 50% MeOH fraction (64.2 g) was further separated by silica gel column chromatography using dichloromethane/methanol step gradients (50:1 to 5:1, v/v) to afford three subfractions (Fr.1–Fr.3). Details are shown in Supplementary materials.

### X-ray crystallography data

X-ray data of compounds **1** and **15** are shown in Supplementary materials.

### Cell culture and treatment

HaCaT keratinocytes (Kunming Zoo Institute, Chinese Academy of Sciences) were maintained in DMEM (Servicebio) containing 10% FBS (Gibco), penicillin (100 U/mL), and streptomycin (100 μg/mL) at 37 °C under 5% CO₂. For UV exposure, cells at ~ 80% confluency were pretreated with compounds for 24 h, followed by irradiation using a solar simulator (UVA: 250 mJ/cm^2^; UVB: 25.67 J/cm^2^). After the radiation, the cells were subsequently replaced with a fresh DMEM and incubated for 24 h before the cell viability analysis.

### Cytotoxic assay

We evaluated cytotoxicity using the MTT assay (Beyotime Institute of Biotechnology, C0009M) with modifications based on earlier descriptions [35]. Detailed experimental protocol is attached in the Supplementary materials.

### Quantitative reverse transcription-PCR (qRT-PCR)

HaCaT cells (1 × 10⁶ cells/mL) were plated in 6-well plates and pretreated with compound **42** (10, 20, or 40 μM) for 24 h. Cells were then UV-irradiated and incubated for an additional 24 h. Total RNA isolation was performed with Biosharp RNA Extraction Reagent (BLI665A), followed by cDNA synthesis, and then quantitative PCR was carried out. Gene-specific primers of MMP-1, MMP-3, and *β*-actin (sequences in Table S3) were amplificated and detected. *β*-actin was normalized for gene expression.

### Western blots

Protein samples for Western blotting were prepared following established protocols from prior work [36].

### Determination of ROS

ROS generation was assessed with a standardized assay (Catalog E004-1-1, NanJing JianCheng Bioengineering Institute, China). The procedure adhered to the manufacturer’s protocol but included minor adaptations from prior methodologies [37]. Details are shown in the Supplementary materials.

#### MDA, T-SOD, hydroxyproline generation assay

Following treatment, cell supernatants were collected and transferred to fresh microcentrifuge tubes for analysis. Total Superoxide Dismutase (T-SOD) activity, Malondialdehyde (MDA) levels, and hydroxyproline content were quantified using a commercial assay kit and quantified strictly referring to the manufacturer's instructions and supplemented by established methodology [15, 37, 38].

#### Statistical analysis

GraphPad Prism (v.10.1.2) was used for statistical analyses. Data are shown as mean ± SD (n = 3). Significance was tested with one-way ANOVA and Tukey’s post hoc test (*P* < 0.05).

## Supplementary Information

Additional file 1.

## Data Availability

All data is confidential.
