# Supplementary material for: Novel triterpenoids from Ganoderma resinaceum attenuate UV-induced photoaging via modulating Nrf2 and MAPK signaling pathways
Source: Nat Prod Bioprospect. 2026 Jan 9;16(1):5. doi: 10.1007/s13659-025-00558-z (PMC12783492; doi:10.1007/s13659-025-00558-z)
Supplement: Supplementary file 1 — Additional file 1. [file 13659_2025_558_MOESM1_ESM.docx]

**Supplementary material**

**Novel triterpenoids from *Ganoderma resinaceum*attenuate UV-induced photoaging via modulating Nrf2 and MAPK signaling pathways**

Yi Luo,^1,2,3#^ Xiao-Cui Liu,^1,2,3#^ Yu-Jie Li,^1,2,3^ Ming-Hua Qiu,^1,2,3*^ Xing-Rong Peng^1,2,3*^

^1^ State Key Laboratory of Phytochemistry and Natural Medicines, Kunming Institute of Botany, Chinese Academy of Sciences, Kunming 650201, China

^2^ Kunming College of Life Science, University of Chinese Academy of Sciences, Kunming, Yunnan 650204, China

^3^ University of Chinese Academy of Sciences, Beijing 100049, China

^*^Corresponding authors: Telephone: +86-871-65223327, Fax: +86-871-65223325, E-mail: [mhchiu@mail.kib.ac.cn](mailto:mhchiu@mail.kib.ac.cn); E-mail: [pengxingrong@mail.kib.ac.cn](mailto:pengxingrong@mail.kib.ac.cn)

1. **Materials and methods**

**1.1 General information**

Silica gel (200-300) mesh, Qingdao Marine Chemical, Inc.), Lichroprep RP-18 (40-63 μm, Fuji), and Sephadex LH-20 (20-150 μm, Pharmacia) were used for column chromatography. Methanol, chloroform, ethyl acetate, acetone, petroleum ether, n-hexane, and 2-propanol were purchased from Tianjing Chemical Reagents Co. (Tianjin, China). Shimadzu UV-2401PC spectrometer was for Ultraviolet (UV) spectra. Horiba SEPA-300 polarimeter was for optical rotations. Chirascan instrument was used for the determination of CD spectra; Bruker AV-600 MHz (Bruker, Zurich, Switzerland) was used to measure Nuclear magnetic resonance (NMR) spectra with tetramethyl chlorosilane (TMS) as an internal standard for chemical shifts. Electrospray Ionization Mass Spectrometry (ESIMS) and HRTOF-ESIMS were recorded by API QSTAR Pulsar spectrometer. Bruker Tensor-27 instrument by using KBr pellets was used for IR. An Agilent 1100 series instrument equipped with an Agilent ZORBAX SB-C18 column (5 μm, 9.6 mm × 250 mm) was used for high-performance liquid chromatography (HPLC) separation.

**1.2 Fungi material**

*Ganoderma resinaceum* (10 kg) was purchased in June 2016 from the Traditional Chinese Medicine Market in Kunming, Yunnan, China, which was identified by Prof. Yang Zhuliang, Kunming Institute of Botany, Chinese Academy of Science (voucher number: KGR-201606).

**1.3 Extraction and isolation**

The air-dried powder of the fruiting bodies of *G. resinaceum* (10 kg) was extracted with MeOH (50 L) under reflux three times. The extract was suspended in water and extracted successively with petroleum ether and ethyl acetate. The ethyl acetate extract (300 g) was chromatographed over a D101 macroporous resin, eluted successively with a gradient of H_2_O, 20% MeOH/H_2_O, 50% MeOH/H_2_O, 70% MeOH/H_2_O, and 90% MeOH/H_2_O. The 50% MeOH/H_2_O eluate (64.2 g) was subjected to silica gel column chromatography (CC) and eluted with CH_2_Cl_2_/MeOH (50:1, 30:1, 20:1, 10:1, 5:1) to afford three fractions (Fr. 1–Fr. 3).

Fr. 1 (34.6 g) was chromatographed on a silica gel CC to yield three fractions (Fr. 1a– Fr. 1c). Fr. 1b (23.4 g) was treated using reverse silica gel C18 CC and eluted with 40%→50% MeOH/H_2_O to obtain twenty subfractions (Fr. 1b-1→Fr. 1b-20), which were further purified by semi-preparative HPLC (CH_3_CN/H_2_O containing 0.1% formic acid = 30%‒45%, v/v, 3 mL/min) to give compounds **1** (1.2 mg, t*_R_* = 17.2 min), **2** (5.3 mg, t*_R_* = 25.5 min), **3** (4.2 mg, t*_R_* = 16.6 min), **4** (6.1 mg, t*_R_* = 23.6 min), **5** (3.8 mg, t*_R_* = 13.1 min), **6** (5.2 mg, t*_R_* = 17.2 min), **9** (4.3 mg, t*_R_* = 16.5 min), **15** (1.0 mg, t*_R_* = 27.0 min), **31** (10.6 mg, t*_R_* = 27.7 min), **42** (5.6 mg, t*_R_* = 14.5 min), **12** (2.2 mg, t*_R_* = 22.0 min), **13** (3.1 mg, t*_R_* = 18.8 min), **17** (6.2 mg, t*_R_* = 27.0 min), **21** (2.5 mg, t*_R_* = 18.2 min), **24** (2.6 mg, t*_R_* = 27.7 min), **29** (3.1 mg, t*_R_* = 25.1 min), **33** (1.2 mg, t*_R_* = 22.3 min), **34** (3.5 mg, t*_R_* = 14.1 min), and **36** (1.5 mg, t*_R_* = 19.6 min).

Fr. 2 (25 g) was subjected to C18 reverse silica gel CC, eluting with MeOH/H_2_O (30%→70%, v/v) to obtain 15 subfractions (Fr. 2-1→Fr. 2-15). Subsequently, these fractions were treated using semi-preparative HPLC (CH_3_CN/H_2_O containing 0.1% formic acid = 30%‒55%, v/v, 3 mL/min) to yield compounds **7** (12.3 mg, t*_R_* = 20.3 min), **8** (11.0 mg, t*_R_* = 25.5 min), **10** (11.0 mg, t*_R_* = 25.5 min), **11** (4.2 mg, t*_R_* = 31.8 min), **35** (6.4 mg, t*_R_* = 27.5 min), **37** (8.9 mg, t*_R_* = 14.4 min), **14** (3.7 mg, t*_R_* = 20.6 min), **16** (3.1 mg, t*_R_* = 19.1 min), **18** (15.3 mg, t*_R_* = 28.3 min), **19** (17.1 mg, t*_R_* = 29.6 min), **20** (3.0 mg, t*_R_* = 19.9 min), **22** (1.5 mg, t*_R_* = 16.1 min), **23** (2.8 mg, t*_R_* = 16.9 min), **25** (2.7 mg, t*_R_* = 13.9 min), **26** (1.1 mg, t*_R_* = 19.0 min), **27** (2.8 mg, t*_R_* = 17.2 min), **28** (9.5 mg, t*_R_* = 22.6 min), **30** (3.3 mg, t*_R_* = 22.6 min), **32** (1.0 mg, t*_R_* = 27.0 min), **38** (1.0 mg, t*_R_* = 18.9 min), **39** (1.1 mg, t*_R_* = 22.1 min), **40** (1.0 mg, t*_R_* = 25.3 min), **41** (3.2 mg, t*_R_* = 14.3 min), and **43** (2.3 mg, t*_R_* = 23.5 min).

Ganoresinol A (**1**): colorless crystal (MeOH); [α]^20.4^_D_ +120.2 (*c* 0.1, MeOH); UV (MeOH); λ_max_ (log ε): 237 (4.14), and 196 (3.86); ^1^H NMR and ^13^C NMR data: see Table 1; HRMS (ESI-TOF) *m/z*: 517.2364 [M + Cl]^‒^ (calcd for C_29_H_38_O_6_Cl, 517.2362).

Crystal data for **1**: C_29_H_38_O_6_•H_2_O, *M* = 500.61, *a* = 12.2256(15) Å, *b* = 7.6023(11) Å, *c* = 14.738(2) Å, *α* = 90°, *β* = 111.663(6)°, *γ* = 90°, *V* = 1273.0(3) Å^3^, *T* = 100.0(2) K, space group *P*1211, *Z* = 2, *μ*(Cu Kα) = 0.748 mm^-1^, 18906 reflections measured, 4939 independent reflections (*R_int_* = 0.0900). The final *R_1_* values were 0.0576 (*I* > 2*σ*(*I*)). The final *wR*(*F*^2^) values were 0.1501 (*I* > 2*σ*(*I*)). The final *R_1_* values were 0.0701 (all data). The final *wR*(*F*^2^) values were 0.1639 (all data). The goodness of fit on *F*^2^ was 1.092. Flack parameter = 0.02(13).

Ganoresic acid A (**2**): white powder (MeOH); [α]^20.4^_D_ 161.8 (*c* 0.10, MeOH); UV (MeOH); λ_max_ (log ε): 261 (3.79), and 202 (3.78); ^1^H NMR and ^13^C NMR data: see Table 1; HRMS (ESI-TOF) *m/z*: 481.2364 [M + Cl]^‒^ (calcd for C_26_H_36_O_6_Cl, 481.2362).

Ganoresic acid B (**3**): white powder (MeOH); [α]^20^_D_ 102.46 (*c* 0.13, MeOH); UV (MeOH); λ_max_ (log ε): 254 (3.73), 216 (3.97) and 204 (3.99); ^1^H NMR and ^13^C NMR data: see Table 1; HRMS (ESI-TOF) *m/z*: 465.2240 [M + Na]^+^ (calcd for C_26_H_34_O_6_Na, 465.2248).

Ganoresic acid C (**4**): white powder (MeOH); [α]^20.3^_D_ 21.33 (*c* 0.09, MeOH); UV (MeOH); λ_max_ (log ε): 239 (2.23), and 202 (2.47); ^1^H NMR and ^13^C NMR data: see Table 1; HRMS (ESI-TOF) *m/z*: 481.2364 [M + Cl]^‒^ (calcd for C_26_H_38_O_6_Cl, 481.2362).

Ganoresiate D (**5**): white powder (MeOH); [α]^20.2^_D_ 124.6 (*c* 0.10, MeOH); UV (MeOH); λ_max_ (log ε): 251 (3.59), and 196 (3.93); ^1^H NMR and ^13^C NMR data: see Table 1; HRMS (ESI-TOF) *m/z*: 507.2963 [M ‒ COOH]^‒^ (calcd for C_27_H_42_O_6_, 507.2963).

Ganoresinol B (**6**): white powder (MeOH); [α]^20.4^_D_ 127.7 (*c* 0.08, MeOH); UV (MeOH); λ_max_ (log ε): 254 (3.70), and 200 (3.49); ^1^H NMR and ^13^C NMR data: see Table 2; HRMS (ESI-TOF) *m/z*: 417.2646 [M ‒ H]^‒^ (calcd for C_25_H_38_O_5_, 417.2649).

Ganoresinol C (**7**): white powder (MeOH); [α]^19.9^_D_ 205.8 (*c* 0.10, MeOH); UV (MeOH); λ_max_ (log ε): 253 (3.80), and 201 (3.70); ^1^H NMR and ^13^C NMR data: see Table 2; HRMS (ESI-TOF) *m/z*: 415.0000 [M ‒ H]^‒^ (calcd for C_25_H_36_O_5_, 415.0000).

Ganoresinol D (**8**): white powder (MeOH); [α]^19.9^_D_ 206.2 (*c* 0.20, MeOH); UV (MeOH); λ_max_ (log ε): 253 (2.84), and 202 (2.79); ^1^H NMR and ^13^C NMR data: see Table 2; HRMS (ESI-TOF) *m/z*: 437.2300 [M + Na]^+^ (calcd for C_25_H_34_O_5_, 437.2298).

Ganoresinone A (**9**)*:* white powder (MeOH); [α]^20.5^_D_ 92.0 (*c* 0.20, MeOH); UV (MeOH); λ_max_ (log ε): 250 (3.69), and 200 (3.71); ^1^H NMR and ^13^C NMR data: see Table 2; HRMS (ESI-TOF) *m/z*: 439.2090 [M + Na]^+^ (calcd for C_24_H_32_O_6_, 439.2091).

Ganoresinone B (**10**)*:* white powder (MeOH); [α]^20.5^_D_ 114.5 (*c* 0.36, MeOH); UV (MeOH); λ_max_ (log ε): 245 (3.94), and 202 (3.79); ^1^H NMR and ^13^C NMR data: see Table 2; HRMS (ESI-TOF) *m/z*: 399.2176 [M ‒ H]^‒^ (calcd for C_24_H_32_O_5_, 399.2177).

Ganoresinone C (**11**): white powder (MeOH); [α]^19.5^_D_ 257.5 (c 0.21, MeOH); UV (MeOH); λ_max_ (log ε): 244 (4.11), and 202 (3.98); ^1^H NMR and ^13^C NMR data: see Table 3; HRMS (ESI-TOF) *m/z*: 421.1988 [M + Na]^+^ (calcd for C_24_H_30_O_5_, 421.1985).

Ganoresinone D (**15**)*:* white powder (MeOH); [α]^19.5^_D_ 198.2 (*c* 0.10, MeOH); UV (MeOH); λ_max_ (log ε): 245 (3.67), and 202 (3.77); ^1^H NMR and ^13^C NMR data: see Table 3; HRMS (ESI-TOF) *m/z*: 387.2011 [M ‒ H]^‒^ (calcd for C_24_H_30_O_5_, 397.2020).

Crystal data for **15**: C_24_H_34_O_5_, *M* = 402.51, *a* = 10.7738(3) Å, *b* = 7.1097(2) Å, *c* = 13.9891(4) Å, *α* = 90°, *β* = 102.0780(10)°, *γ* = 90°, *V* = 1047.82(5) Å^3^, *T* = 150.(2) K, space group *P*1211, *Z* = 2, *μ*(Cu Kα) = 0.706 mm^-1^, 15116 reflections measured, 3777 independent reflections (*R_int_* = 0.0909). The final *R_1_* values were 0.0515 (*I* > 2*σ*(*I*)). The final *wR*(*F*^2^) values were 0.1235 (*I* > 2*σ*(*I*)). The final *R_1_* values were 0.0593 (all data). The final *wR*(*F*^2^) values were 0.1274 (all data). The goodness of fit on *F*^2^ was 1.101. Flack parameter = 0.02(12).

Ganoresinic acid A (**31**)*:* white powder (MeOH); [α]^19.9^_D_ 96.0 (*c* 0.11, MeOH); UV (MeOH); λ_max_ (log ε): 234 (3.93), and 201 (3.79); ^1^H NMR and ^13^C NMR data: see Table 3; HRMS (ESI-TOF) *m/z*: 533.2517 [M + Na]^+^ (calcd for C_30_H_38_O_7_, 533.2510).

Methyl ganoresinate (**35**)*:* white powder (MeOH); [α]^19.8^_D_ 191.8(*c* 0.10, MeOH); UV (MeOH); λ_max_ (log ε): 240 (4.05), and 198 (3.86); ^1^H NMR and ^13^C NMR data: see Table 3; HRMS (ESI-TOF) *m/z*: 549.2623 [M + Na]^+^ (calcd for C_31_H_42_O_7_, 549.2821).

Methyl ganoderenate F (**37**)*:* white powder (MeOH); [α]^19.8^_D_ 91.3 (*c* 0.37, MeOH); UV (MeOH); λ_max_ (log ε): 243 (4.12), and 196 (3.80); ^1^H NMR and ^13^C NMR data: see Table 3; HRMS (ESI-TOF) *m/z*: 525.2845 [M + H]^+^ (calcd for C_31_H_40_O_7_, 525.2847).

Ganoresinic acid B (**42**)*:* white powder (MeOH); [α]^19.8^_D_ 142.3 (*c* 0.11, MeOH); UV (MeOH); λ_max_ (log ε): 255 (3.75), and 200 (3.47); ^1^H NMR and ^13^C NMR data: see Table 3; HRMS (ESI-TOF) *m/z*: 539.2972 [M + Na]^+^ (calcd for C_30_H_40_O_7_, 539.2979).

Twenty-seven known compounds were identified as lucidone D (**12**) [1], lucidone A (**13**) [1], 15*β*-hydroxy-4,4,14*α*-trimethyl-3,7,11,20-tetraoxo-5*α*-pregn-8-ene (**14**) [2], lucidone E (**16**) [1], lucidone F (**17**) [1], lucidadone H (**18**) [1], lucidone B (**19**) [1], lucidone K (**20**) [3], ganoderic acid AM1 (**21**) [4], ganoderic acid K (**22**) [5], ganoderic acid B (**23**) [5], 3*β*,7*β*-dihydroxy-11,15,23- trioxolanost-8,16-dien-26-oic acid (**24**) [6], 3*β*,7*β*-dihydroxy-11,15,23- trioxolanost-8,16-dien-26-oic acid methyl ester (**25**) [7], ganoderenic acid C (**26**) [7], 3*β*,15*α*-dihydroxy-7,11,23-trioxo-5*α*-lanosta-8,16-dien-26-oic acid (**27**) [8], resinacein O (**28**) [9], ganoderic acid C 6 (**29**) [10], ganoderic acid H (**30**) [11], ganoderic acid E (**32**) [10], methyl ganoderate E (**33**) [12], 3,11,15,23-tetraoxo-27ξ-lanosta-8,16-dien-26-oic acid (**34**) [13], ganoderic acid D (**36**) [10], (20*E*)-15*β*-hydroxy-3,7,11,23-tetraoxolanost-8,20(22)-dien-26-oic acid (**38**) [14], 12*β*-hydroxyganoderenic F (**39**) [15], (20*E*)-3*β*,15*α*-dihydroxy-7,11,23-trioxo-5α-lanosta-8,20(22)-dien-26-oic acid (**40**) [14], ganoderic acid β (**41**) [16], applanoxidic acid B (**43**) [17] by comparing their 1D NMR spectroscopic data with literature reported previously.

**1.4 X-ray crystallography data**

Crystal structures of **1** and **15** were solved by direct methods using SHELXS-97 (Sheldrich, G.M. University of Gottingen; Gottingen, Germany, 1997) and the full-matrix least-squares deposited in the Cambridge Crystallographic Data Centre (deposition number: 2259021 for **1**, 2259022 for **15**).

**1.5 Cell culture and treatment**

HaCaT cells were purchased from Kunming Institute of Zoology, Chinese Academy of Science. HaCaT cells were cultured in Dulbecco′s Modified Eagle Medium (DMEM) (Servicebio, China, G4511) supplemented with 10 % fetal bovine serum (FBS) (Gibco, USA, 11875), 100 U/ml penicillin and 100 mg/ml streptomycin at 37 ℃ in a humidified atmosphere incubator with 5 % CO_2_ and 95 % humidity. Cells were seeded in prewarmed growth medium in 6-well plates or 12-well plates. When the cell density grew to about 80%, the cells were grouped into four groups: control group, UV-treated group, UV + resveratrol-treated group (10 μM), and UV + compounds-treated group. Resveratrol was used as a positive medicine. Compounds were applied directly to the culture medium for treatment. Then HaCaT cells were irradiated at a series of UVA, UVB, and UVA/UVB doses, respectively. After the radiation, the cells were subsequently replaced with a fresh DMEM and incubated for 24 h before the cell viability analysis.

**1.6 Cytotoxic assay**

A standard MTT assay was performed to determine cytotoxicity according to the manufacturer′s protocol (Beyotime Institute of Biotechnology, China, C0009M) and previously reported methods to improve [18]. HaCaT cells were inoculated in 96-well plates at a density of 8×10^3^ cells/well, and incubated at 37°C in a 5% CO_2_ incubator for 24 h before adding compounds. Chemicals were applied directly to the culture medium for 24 h treatment, the cells were treated with 3-(4,5-dimethyl-2-thiazolyl)-2,5-diphenyltetrazolium bromide thiazolium blue (MTT, 5 mg/ml) solution for 4 h. The formazan was dissolved in 100 μL of dimethylsulfoxide (DMSO). Absorbance was measured at 490 nm by a full-wavelength enzyme marker (Molecular Devices, USA, SpectraMaxiD3). The results were expressed as a percentage of the absorbance value of the untreated control cells relative to the absorbance value of the treated cells.

**1.7 Quantitative reverse transcription- PCR (qRT-PCR)**

HaCaT cells (1×10^6^ cells/mL) were cultured with indicated concentration of **42** (10, 20, and 40 μM) in 6-well plates for 24 h and then exposed to UV-irradiation, and further incubated for 24 h. Total RNA was isolated from HaCaT cells using Total RNA Isolation Reagent (Biosharp, China, BS258A ) and RNA Extraction Buffer (Biosharp, China, BLI665A) according to the manufacturer′s protocol. The cDNA was synthesized from 1 µg RNA by using RevertAid First Strand cDNA Synthesis Kit (Thermo Scientific, USA, K1621) to reverse transcriptional reaction. The qPCR amplification was performed with PowerUp™ SYBR™ Green Master Mix (2×) (Thermo Scientific, USA, 2961636). The PCR primers for MMP-1, MMP-3 and *β*-actin were obtained from Shenggong Biotechnology (Shanghai) Co., Ltd. Primer sequences are listed in **Table S3**. Finally, the real-time fluorescence quantitative PCR instrument (Thermo Scientific, USA, Applied Biosystems® QuantStudio™ 7 Flex) was used to manipulate and analyses the data obtained.

**1.8 Western blots**

For western blotting, the method described in our previous studies was used [19]. Total proteins were prepared using the protein lysis buffer (Beyotime Institute of Biotechnology, China, P0013). Nuclear and cytoplasmic proteins were separated and extracted using the Nuclear and Cytoplasmic Protein Extraction Kit (Beyotime Institute of Biotechnology, China, P0028) following the manufacturer′s instructions. Protein concentration was determined using the BCA protein assay kit (Beyotime Institute of Biotechnology, China, P0012). A total of 20 μg protein was separated by 10 % sodium dodecyl sulfate - polyacrylamide gel electrophoresis (SDS-PAGE), then was transferred to a polyvinylidene fluoride (PVDF) microporous membrane (Immobilon, Merck KGaA, Germany, IPVH00010). The membrane was blocked in 5% nonfat dry milk in TBST (25 mM Tris–HCl, 137 mM NaCl, 2.65 mM KCl, 0.05% Tween 20, pH 7.4) for 2 h at room temperature. The membrane was incubated with primary antibodies (GAPDH, glyceraldehyde-3-phosphate dehydrogenase [Proteintech, China, 60004-1-Ig]; ERK1/2 [ProMab, 30014], Phospho-ERK1/2 (Thr222/Tyr205) [ProMab, China, P44433], MAPK [ProMab, China, 20092], Phospho-MAPK (Thr180/Tyr182) [ProMab, China, P20351], JNK [ProMab, China, 30369], Phospho-JNK (Thr183) [ProMab, China, P20300], Nrf2 [Proteintech, China, 16396-1-AP], Keap1 [Proteintech, China, 10503-2-AP]) overnight at 4 ℃. The membranes were washed 3 times with TBST with Tween 20 [0.1 %; Sigma, P1379]), each time for 10 min, followed by incubation with the peroxidase-conjugated anti-mouse [Huabio, China, HA1006] or anti-rabbit [Huabio, China, HA1001] IgG (1:20000) for 1 h at room temperature. The epitope was visualized using an ECL Western blot detection kit (Bio-Rad, USA, 10026386 Rev B). ImageJ software (National Institutes of Health, Bethesda, Maryland, USA) was used to evaluate the grayscale values of bands. GAPDH was used as a loading control for quantifying the densitometry of target protein.

**1.9 Determination of ROS**

For the measurement of ROS, a standard ROS assay was performed to determine generation according to the manufacturer′s protocol (NanJing JianCheng Bioengineering Institute, China, E004-1-1) and a previously described method with a slight modification [20]. HaCaT cells (1×10^6^ cells/mL) were grown to 70–80% confluence then were cultured with compounds in 6-well plates for 24 h and then exposed to UV-irradiation, and further incubated for 24 h. Then, the cells were washed with phosphate-buffered saline (PBS) (Servicebio, China, G4202) for three times and treated with 20, 70-Dichlorofluorescein diacetate (DCFH-DA) (5 µM) at 37℃ for 30min. Next, aspirated the culture medium, used 0.25% Trypsin-EDTA (1×) (NCM Biotech, China, C100C1) to digest the cells, and then collected all the cell suspension into a 1.5mL EP tube. The cells were washed twice with PBS to fully remove the DCFH-DA that had not entered the cells, and centrifuged at 1000 rpm/min for 5 min, the supernatant was aspirated and the cells were resuspended with PBS for the assay. Finally, fluorescence intensity was measured at excitation and emission wavelengths of 485 and 528 nm, respectively, by a fluorescence microplate reader (Molecular Devices, USA, SpectraMaxiD3).

**1.10 MDA, T-SOD, Hydroxyproline generation assay**

Collected the supernatants of treated cells and transferred to a new tube and used for assay. MDA was measured by Malondialdehyde (MDA) assay kit (TBA method) (NanJing JianCheng Bioengineering Institute, China, A003-1-2) in accordance with the manufacturer′s protocol of the Malondialdehyde (MDA) test kit and a previously described method [20]. Finally, the full-wavelength enzyme marker was used to measure the absorbance value of each tube at 532 nm.

T-SOD was measured by Total Superoxide Dismutase (T-SOD) assay kit (Hydroxylamine method) (NanJing JianCheng Bioengineering Institute, China, A001-1-2) in accordance with the manufacturer′s protocol and a previously described method [21]. Finally, the full-wavelength enzyme marker was used to measure the absorbance value of each tube at 550 nm.

The hydroxyproline assay kit (NanJing JianCheng Bioengineering Institute, China, A030-1-1) was chosen for hydroxyproline generation assay. Collected the supernatants from treated cells, according to the manufacturer′s protocol for determination of hydroxyproline [22]. Finally, the full-wavelength enzyme marker was used to measure the absorbance value of each tube at 550 nm.

**1.11 Statistical analysis**

The results were analyzed by GraphPad Prism 10.1.2 software and the significant difference post hoc test was carried out by one-way analysis of variance (ANOVA) with *p* < 0.05 being considered statistical significance. All results are shown as the means ± SD.

**Structural elucidation**

HRESIMS analysis of ganoresic acid B (**3**) showed an [M + Na]⁺ ion at*m/z* 465.2240 (calcd. 465.2248), establishing the molecular formula C₂₆H₃₄O₆. Its ¹D NMR data (**Table 1**) closely resembled those of **2** except for key differences: one *sp*² CH and one *sp*² C replaced a CH_2_ and CH, respectively. The location of the double bond at C-20/C-22 was supported by the HMBC interactions (**Fig. 2**) from H₃-21 to C-17, C-20 (*δ*_C_ 156.7), and C-22 (*δ*_C_ 119.2), as well as from H-22 (*δ*_H_ 5.75, s) to C-17, C-20, and C-23 (*δ*_C_ 169.6). The *β*-orientation of the 3-OH group was confirmed by the key ROESY cross-peak between H-3 and H-5. Additionally, the ROESY interactions of H-22 with H-16 and H-17 established an *E*-configuration for the ∆^20(22)^. Thus, compound **3** is characterized as (20*E*)-3*β*-hydroxy-24,25,26,27-tetranorlanosta-8,20(22)-dien-7,11,15-trioxo-23-oic acid.

Ganoresic acid C (**4**) was assigned the molecular formula as C₂₆H₃₈O₆ based on HRESIMS analysis ([M + Cl]⁻ at *m/z* 481.2364, calcd. 481.2362). Comparative analysis of its 1D NMR spectrum (**Table 1**) with **2** indicated identical structural features, except for the substitution of a ketone carbonyl with an oxymethine group (*δ*_C_ 67.8). Key HMBC correlations from the oxymethine proton (*δ*_H_ 4.83, t, *J* = 7.5 Hz) to C-5, C-8, C-9, and C-14 positioned the hydroxyl group at C-7, further supported by COSY cross peaks (H-5/H₂-6/H-7). The *β*-orientation of both 3-OH and 7-OH was established through ROESY interactions, specifically H-3/H-5 and H-7/H₃-30. Compound **4** is therefore characterized as 3*β*,7*β*-dihydroxy-24,25,26,27-tetranorlanosta-8,20(22)-dien-11,15-dioxo-23-oic acid.

Compound **5** (C₂₇H₄₂O₆, HRESIMS *m/z* 507.2963 [M + COOH ‒ H]⁻) showed NMR features distinct from **4**: (1) a methoxyl group (HMBC: OMe→C-23) and (2) a C-15 oxymethine (δ 4.12) replacing the carbonyl, evidenced by HMBC (H₃-30/H-17→C-15; H-15→C-8/C-13–C-17) (**Fig. 2**). ROESY data assigned *β* orientations to 3-OH and 7-OH (H-3/H-5/H-7/H₃-30 correlations) and *α* to 15-OH (H-15/H₃-18) (**Fig. 3**). Thus, **5** is characterized as methyl 3*β*,7*β*,15*α*-trihydroxy-24,25,26,27-tetranorlanosta-8,20(22)-dien-11-oxo-23-oate, designated ganoresiate D.

Comparative analysis of 1D NMR data between **6** and **7** revealed identical pentanorlanostane skeletons, except for the replacement of an oxygenated methine in **6** by a ketone carbonyl in **7**. The observed HMBC correlations from H-5 to C-3, C-4, C-19, and the carbonyl carbon (*δ*_C_ 219.7) confirm the oxidation of the C-3 hydroxyl to a ketone. Additionally, the ROESY interaction between H-7 and H_3_-30 unambiguously assigned the *β*-orientation to the 7-hydroxy group. Compound **7** is therefore characterized as 7*β*,22-dihydroxy-23,24,25,26,27-pentanorlanosta-8,9-en-3,11,15-trione and assigned the trivial name ganoresinol C.

Ganoresinol D (**8**) was assigned the molecular formula C_25_H_34_O_5_ based on HRESIMS data, which showed a sodium adduct ion at *m/z* 437.2300 [M + Na]⁺ (calculated 437.2298). The 1D NMR spectra of **8** closely resembled those of compound **7**, but with a key difference: **8** featured an *α*,*β*-unsaturated carbonyl group in place of the oxygenated methine moiety present in **7**. Additionally, HMBC correlations revealed an interaction between H-5 and the carbonyl carbon (*δ*_C_ 201.6) (see **Fig. 4**). Concurrently, the ^13^C NMR spectrum of **8** displayed a high-field shift of C-8 (*δ*_C_ 159.9 → 147.7) and a low-field shift of C-9 (*δ*_C_ 142.3 → 151.4), confirming that the carbonyl group was positioned at C-7. Therefore, the structure of **8** was established to be 3,7,11,15-tetraoxo-23,24,25,26,27-pentanorlanosta-8,9-en-22-ol and named ganoresinol D.

HRESIMS analysis of compound **10** detected a [M – H]⁻ ion at *m/z* 399.2176 (calcd. 399.2177), consistent with the molecular formula C₂₄H₃₂O₅. ¹H and ¹³C NMR data confirmed a hexanorlanostane triterpenoid skeleton structurally similar to lucidone A (**33**) [30], but with notable differences: (1) a significant upfield shift of the C-15 carbonyl (*δ*_C_ 216.1 in lucidone A vs. *δ*_C_ 211.0 in **10**), and (2) the presence of a double bond (*δ*_H_ 6.47, s; *δ*_C_ 133.8, 169.1) in the ¹³C NMR spectrum. HMBC correlations from H-16 to C-13, C-14, C-15, C-17, and C-20, along with the interaction of H₃-21 with C-17, unequivocally positioned the double bond. The *β*-configuration of 3-OH and 7-OH was deduced from ROESY couplings (H-3/H-5/H-7/H₃-30). Compound **10** is therefore characterized as 3*β*,7*β*-dihydroxy-22,23,24,25,26,27-hexanorlanosta-8(9),16(17)-dien-11,15,20-trione and assigned the name ganoresinone B.

HRESIMS analysis of **11** revealed an [M + Na]⁺ ion at*m/z*421.1988 (calcd. for C₂₄H₃₀O₅Na: 421.1985). Comparison of its ¹H NMR data with **10** suggested an identical hexanorlanostane skeleton, differing only in the oxidation of the C-7 hydroxyl to a ketone. This transformation was supported by HMBC cross-peaks between H-5/H₂-6 and the carbonyl carbon. Additionally, ROESY interactions (H-3/H-5) confirmed the *β*-configuration of the 3-hydroxy group. Compound **11** is therefore assigned as 3*β*-hydroxy-22,23,24,25,26,27-hexanorlanosta-8(9),16(17)-dien-7,11,15,20-tetraone and assigned the name ganoresinone C.

HRESIMS analysis of **15** showed an [M + Na]⁺ ion at *m/z* 421.1988 (calcd. for C₂₄H₃₀O₅Na: 421.1985), confirming the molecular formula C₂₄H₃₀O₅, identical to compound **17** [30]. Comparative ¹H/¹³C NMR analysis revealed distinct chemical shifts at C-7 (*δ*_C_ 69.0 for **17**; *δ*_C_ 67.8 for **15**), suggesting differential stereochemistry. The ROESY spectrum of **15** showed that correlations of H-3/H-5, and of H-7/H-15/H_3_-18 illustrated that 3-OH was *β*-oriented, whereas both 7-OH and 15-OH were *α*. Complementary X-ray crystallography (*P*1211, Flack parameter = 0.02, CCDC: 2259022, **Fig. 4**) unequivocally confirmed both planar structure and stereochemical assignments. Thus, **15** is characterized as 3*β*,7*α*,15*α*-trihydroxy-22,23,24,25,26,27-hexanorlanosta-8(9)-en-11,20-dione and assigned the trivial name ganoresinone D.

The 1D NMR profiles of **35** and **34** [31] were nearly identical, with the only distinction being an extra methoxyl substituent in **35**. The combined evidence of the key HMBC interaction from OMe to C-26 and the high-filed shift of C-26 confirmed methylation of the C-26 carboxylate. Consequently, **35** is assigned as methyl 3,7,11,15,23-pentaoxo-lanosta-8(9),16(17)-dien-26-oate and assigned the trivial name methyl ganoresinate.


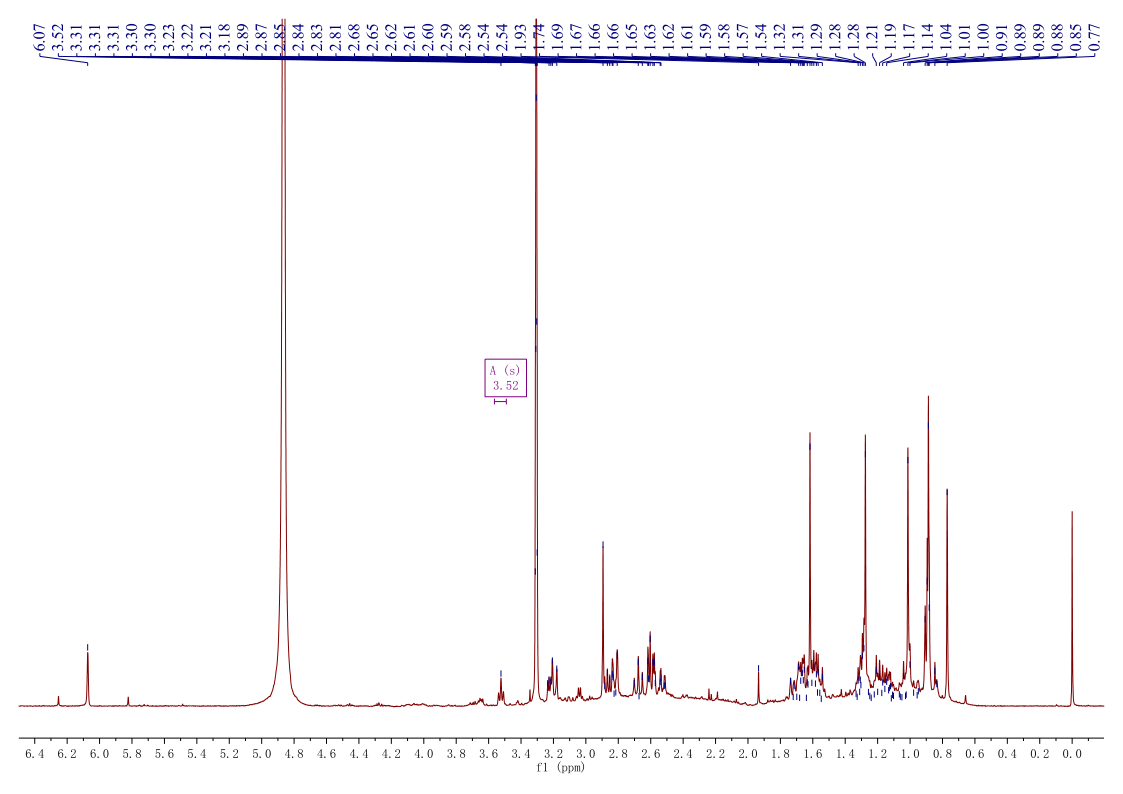


**Figure S1**. ^1^H NMR spectrum (600 MHz, CD_3_OD) of compound **1**.


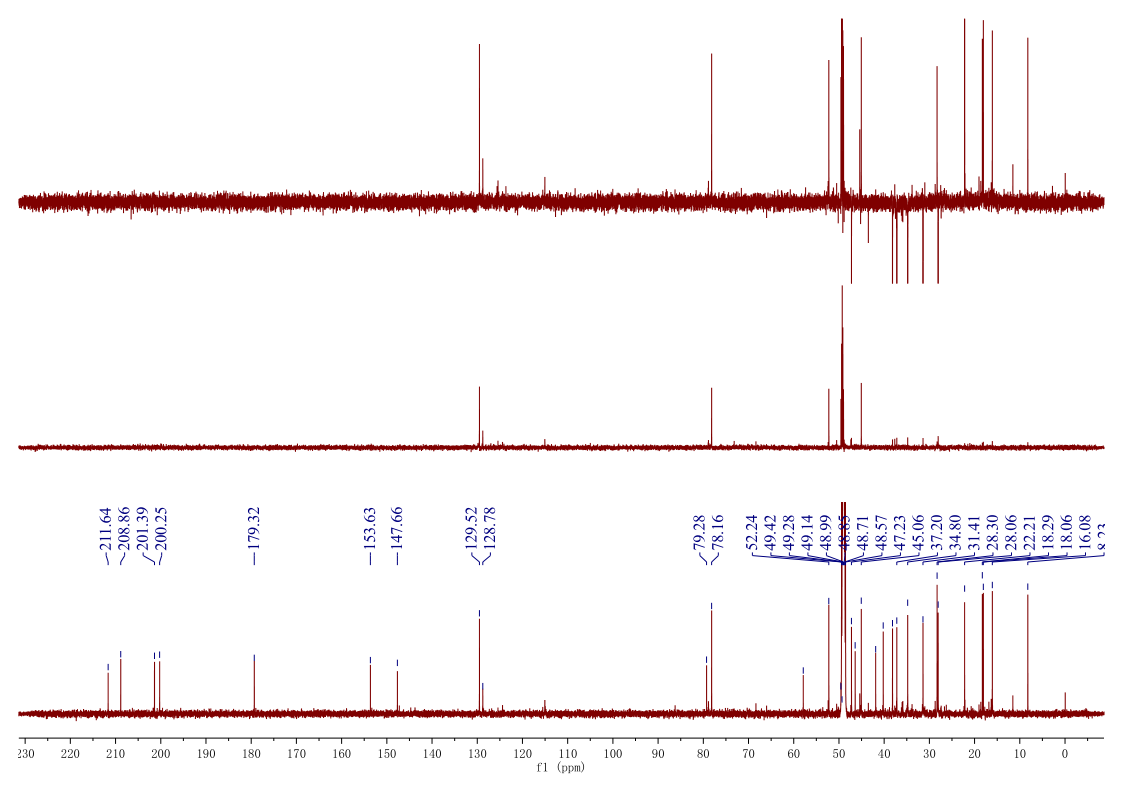


**Figure S2**. ^13^C NMR spectrum (150 MHz, CD_3_OD) of compound **1**.


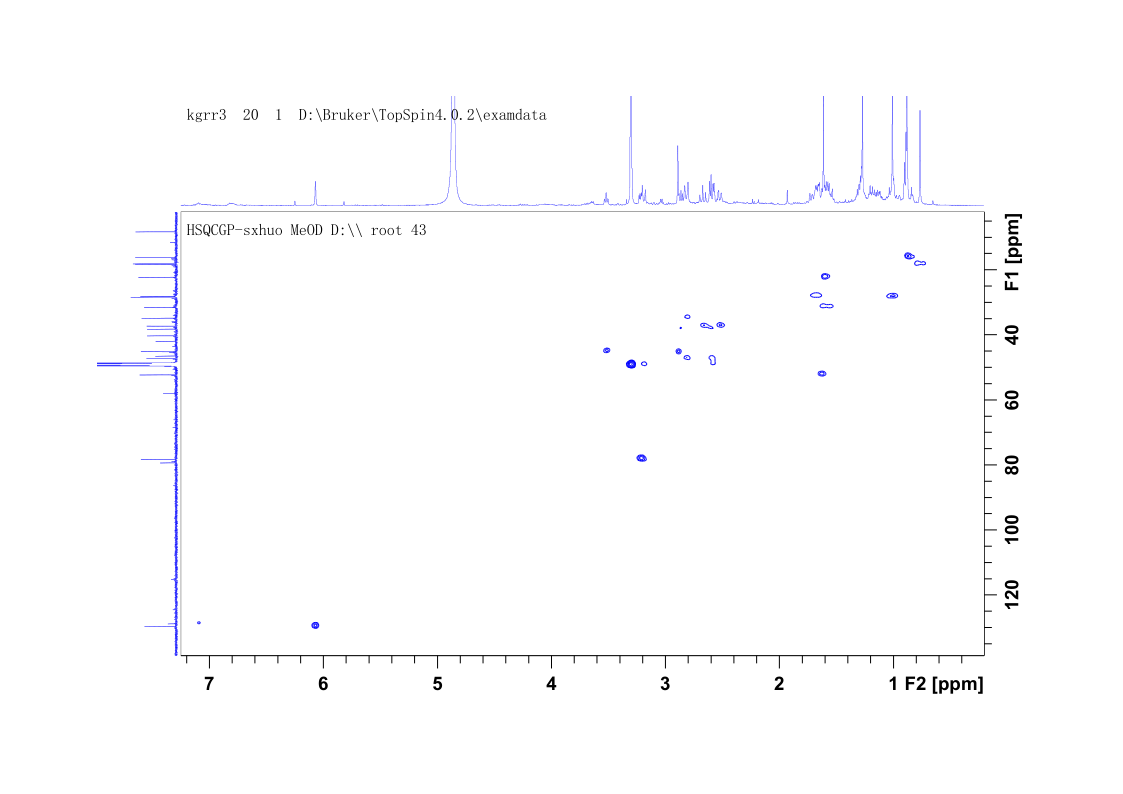


**Figure S3**. HSQC spectrum (600/150 MHz, CD_3_OD) of compound **1**.


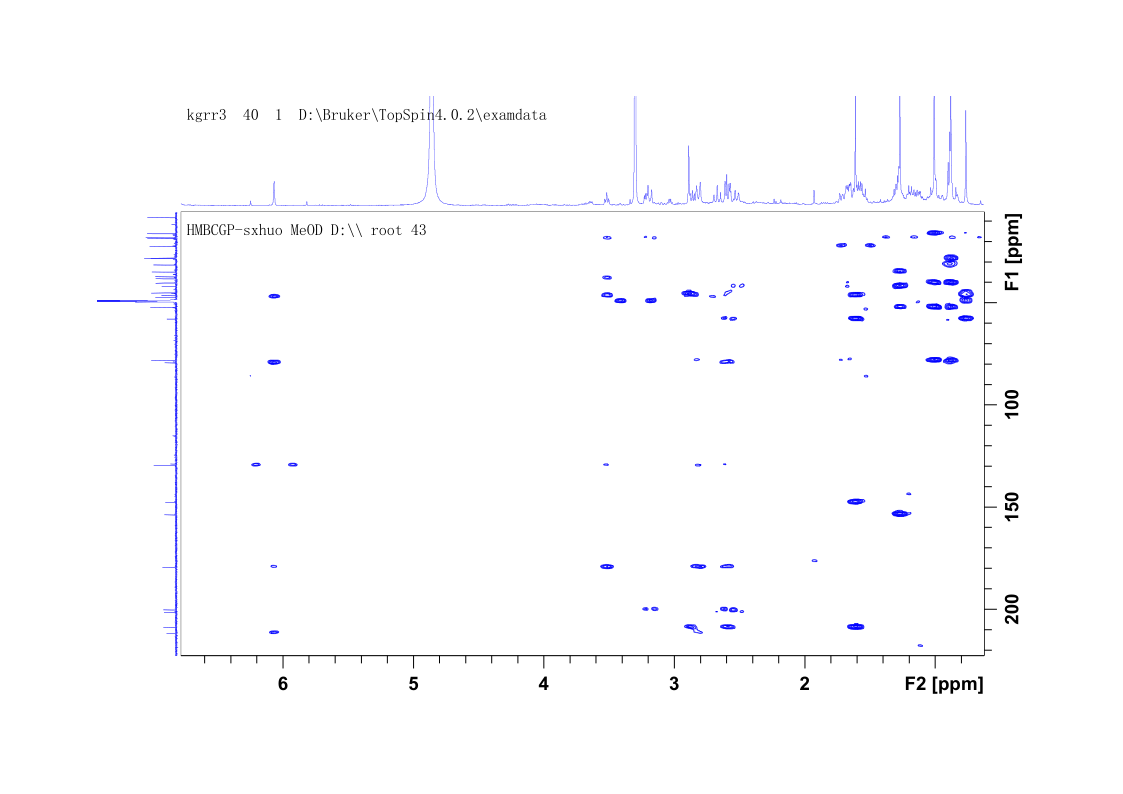


**Figure S4**. HMBC spectrum (600/150 MHz, CD_3_OD) of compound **1**.


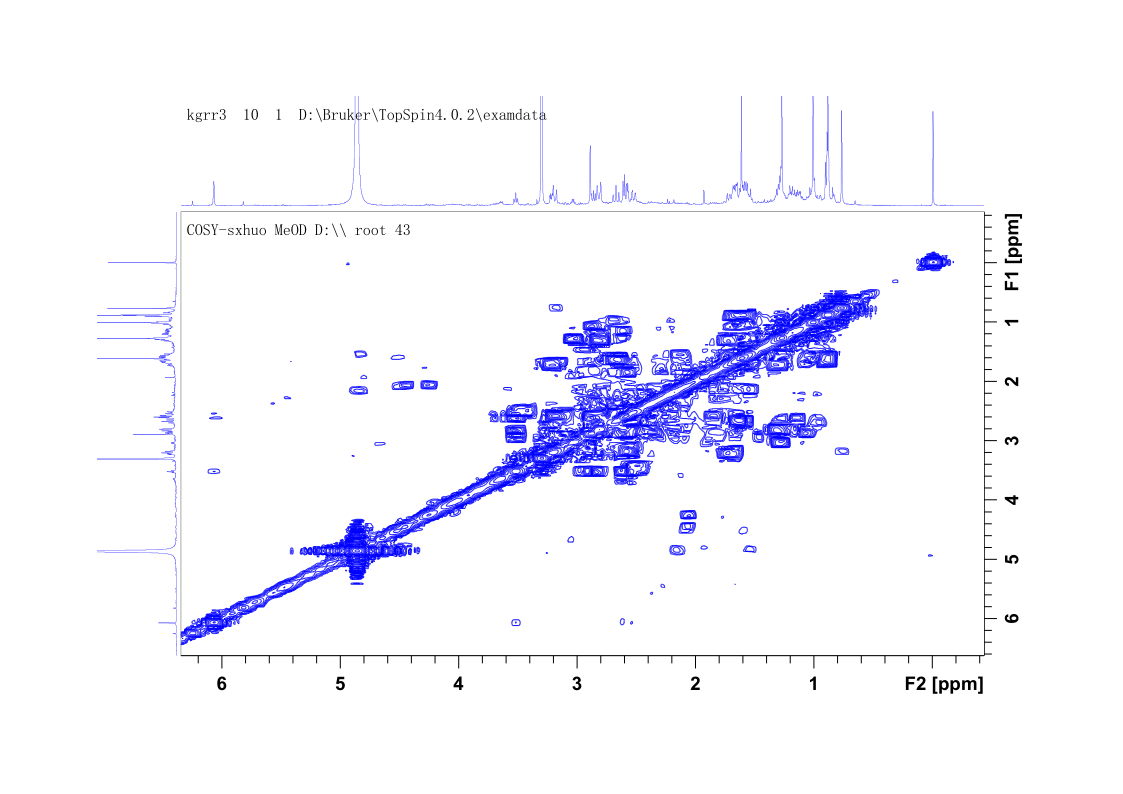


**Figure S5**. ^1^H-^1^H COSY spectrum (600 MHz, CD_3_OD) of compound **1**.


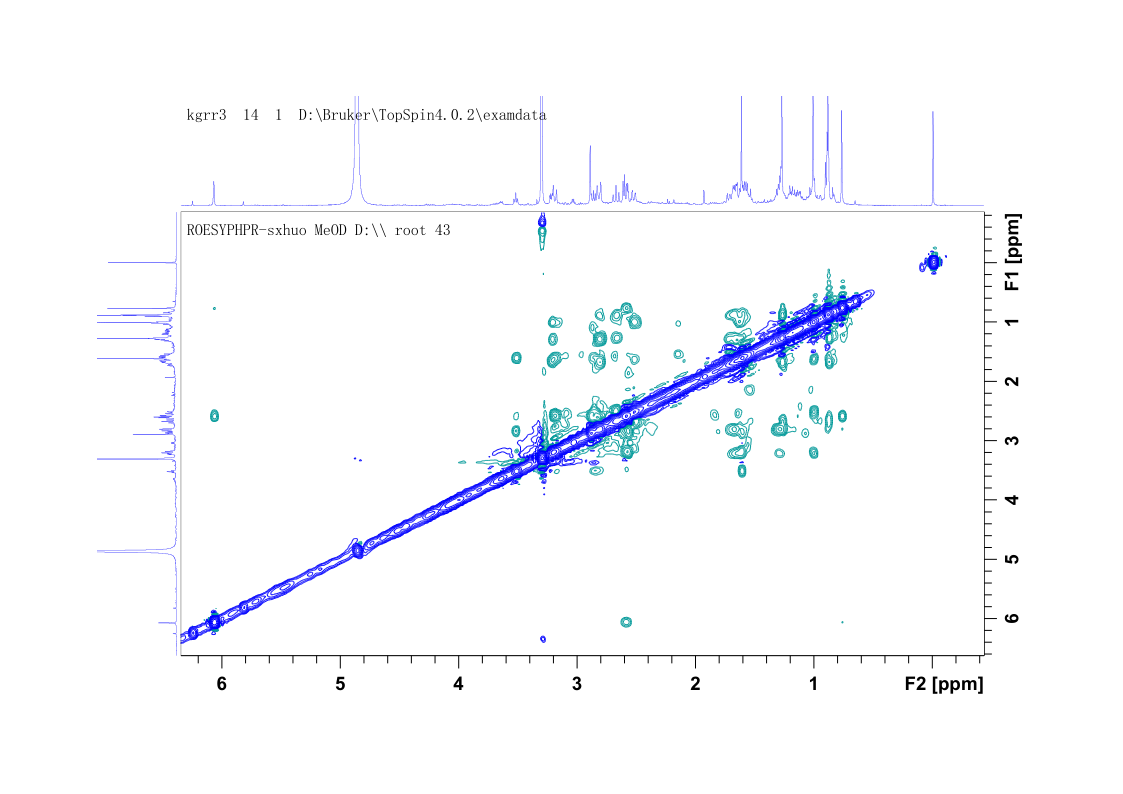


**Figure S6**. ROESY spectrum (600 MHz, CD_3_OD) of compound **1**.


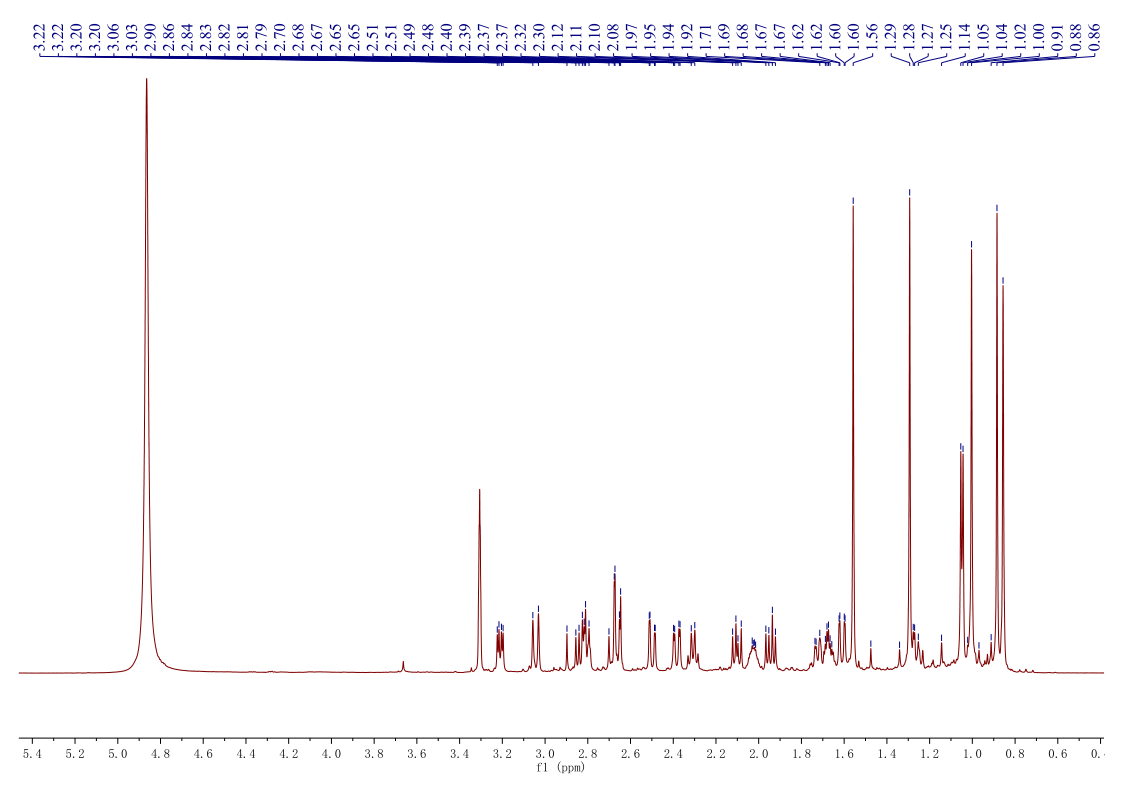


**Figure S7**. ^1^H NMR spectrum (600 MHz, CD_3_OD) of compound **2**.


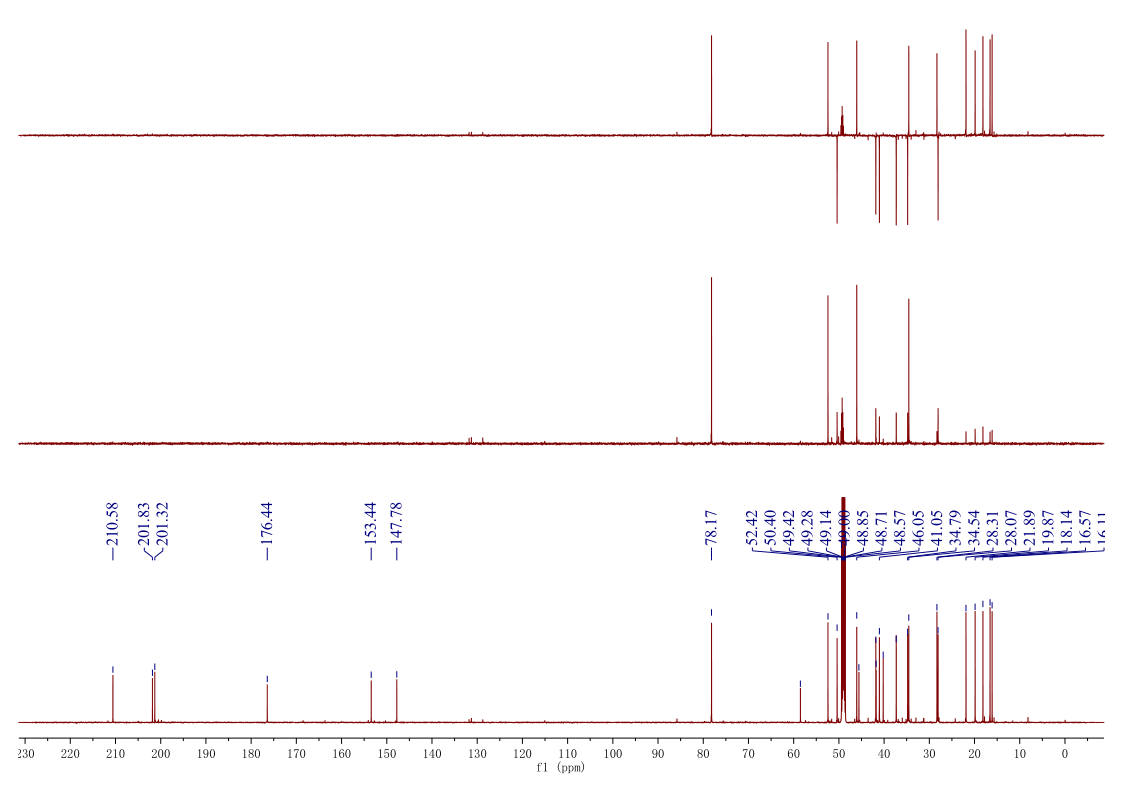


**Figure S8**. ^13^C NMR spectrum (150 MHz, CD_3_OD) of compound **2**.


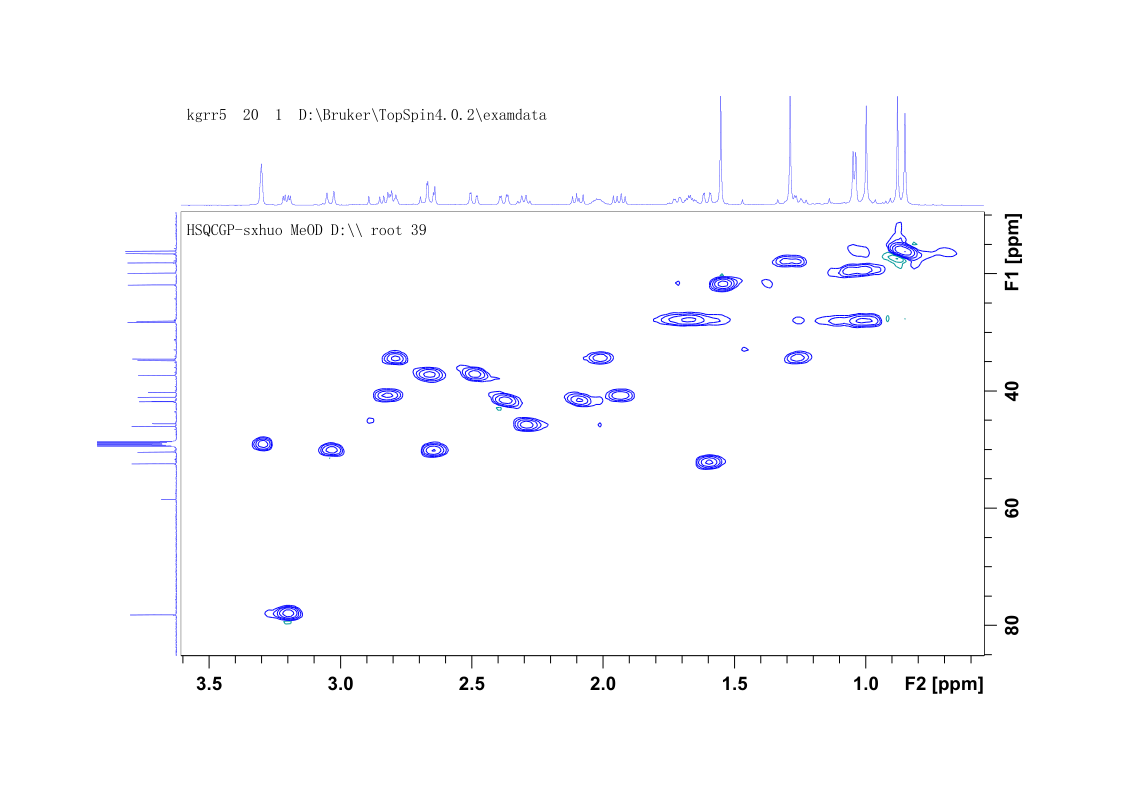


**Figure S9**. HSQC spectrum (600/150 MHz, CD_3_OD) of compound **2**.


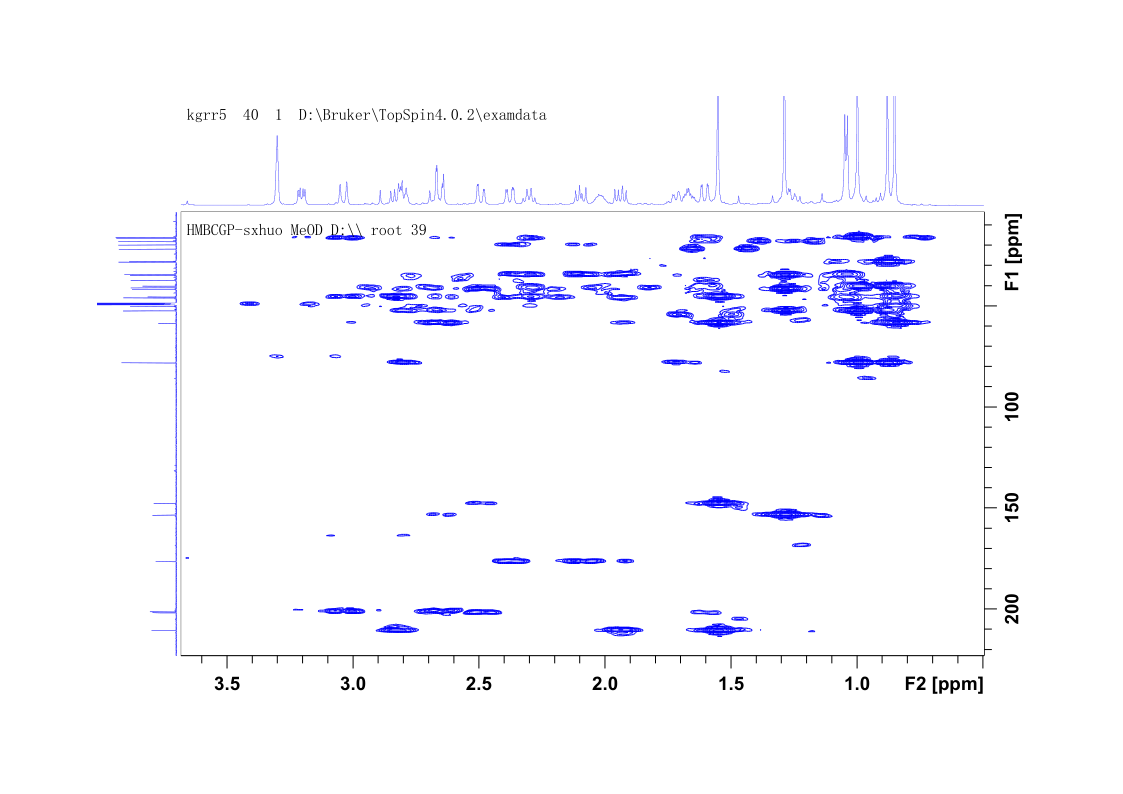


**Figure S10**. HMBC spectrum (600/150 MHz, CD_3_OD) of compound **2**.


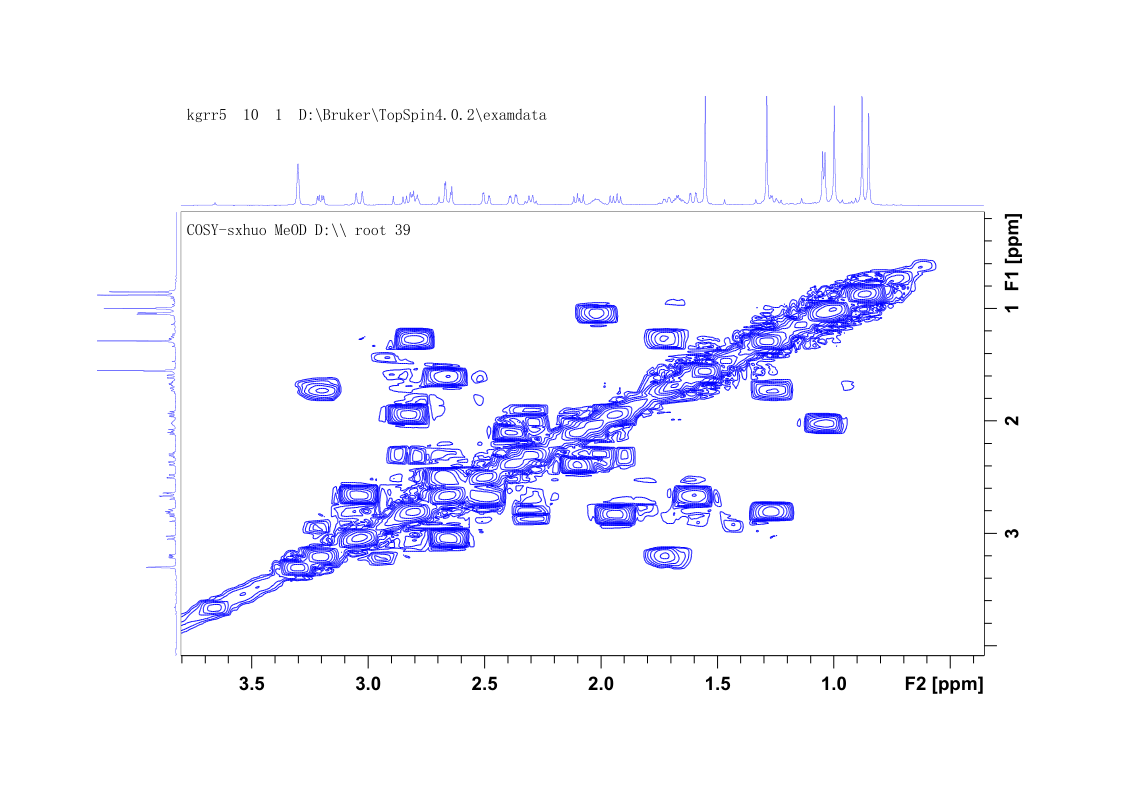


**Figure S11**. ^1^H-^1^H COSY spectrum (600 MHz, CD_3_OD) of compound **2**.


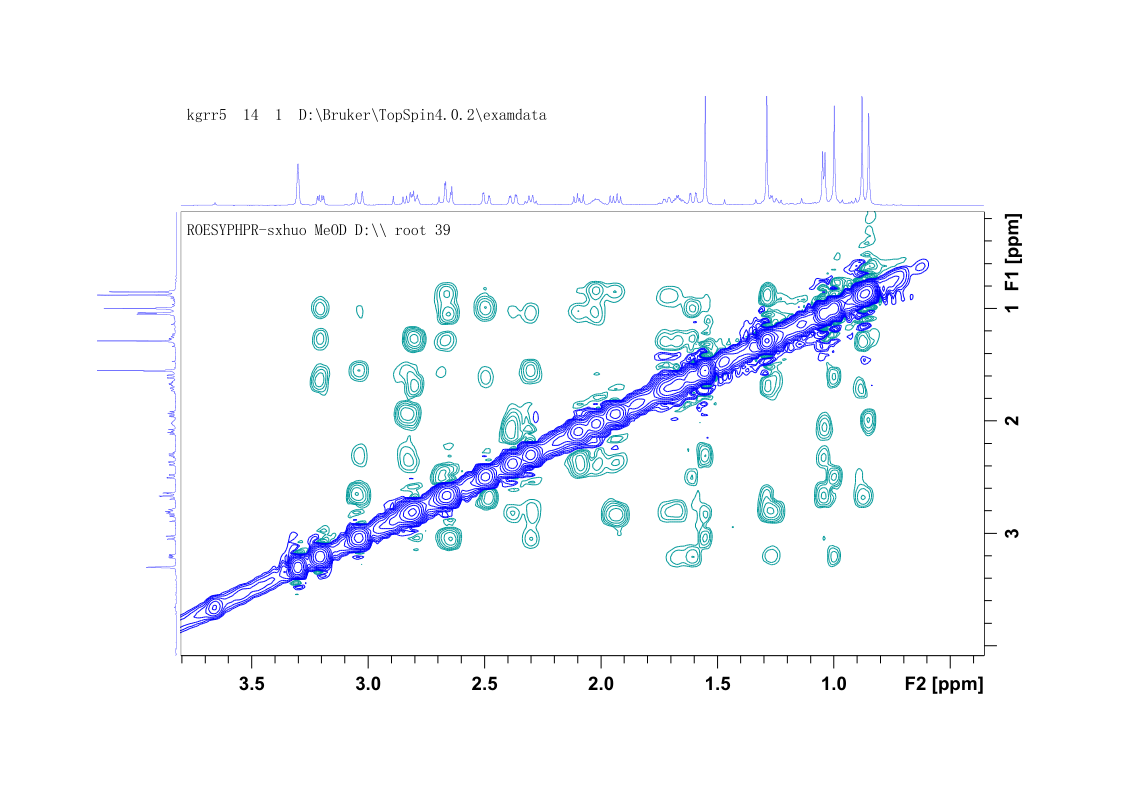


**Figure S12**. ROESY spectrum (600 MHz, CD_3_OD) of compound **2**.


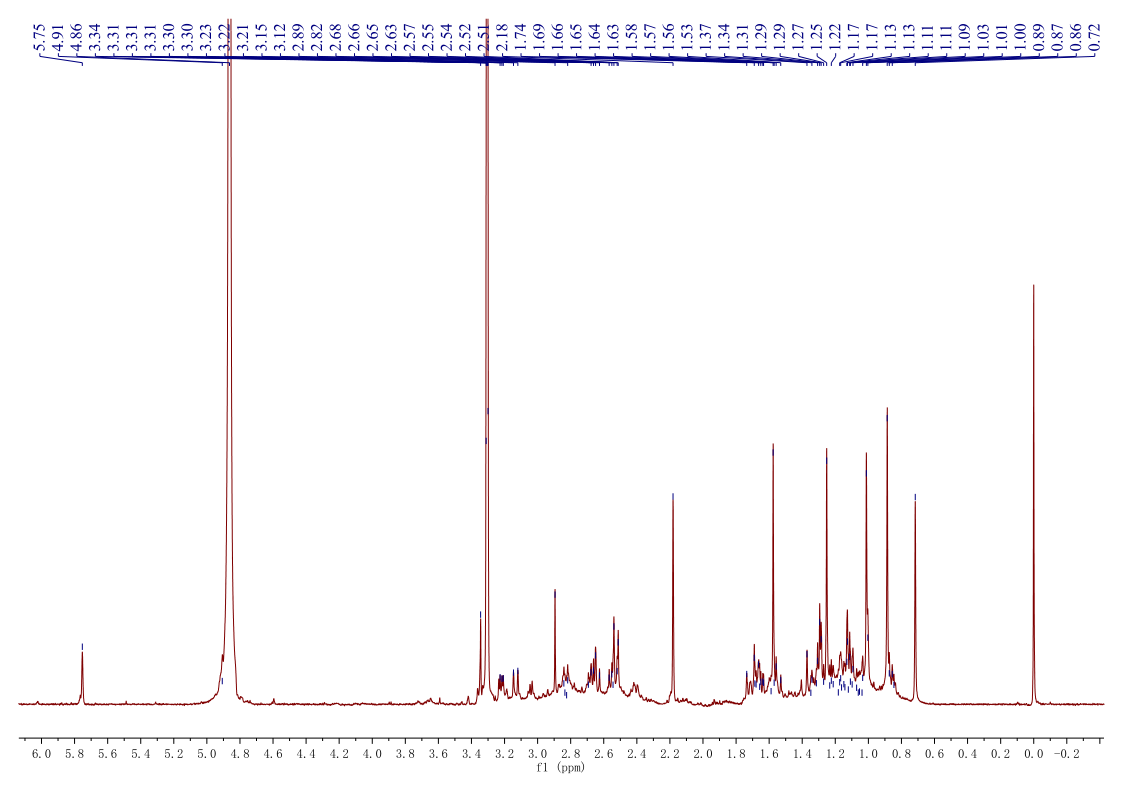


**Figure S13**. ^1^H NMR spectrum (600 MHz, CD_3_OD) of compound **3**.


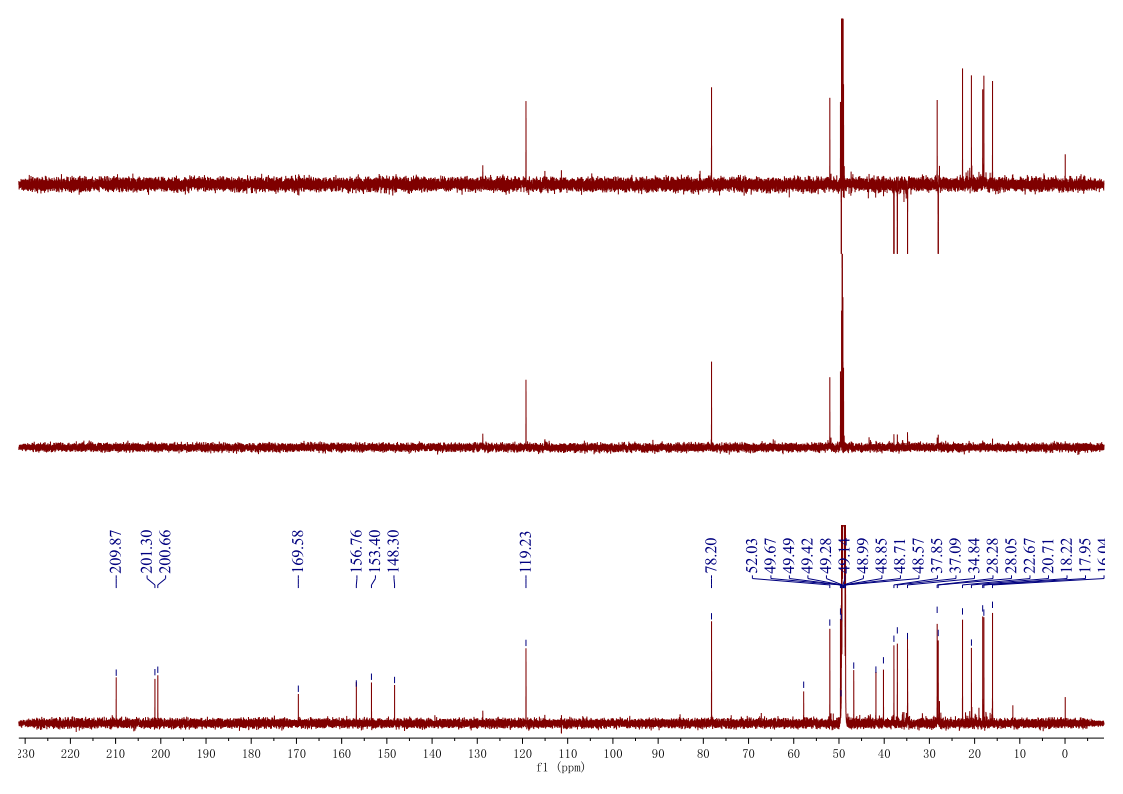


**Figure S14**. ^13^C NMR spectrum (150 MHz, CD_3_OD) of compound **3**.


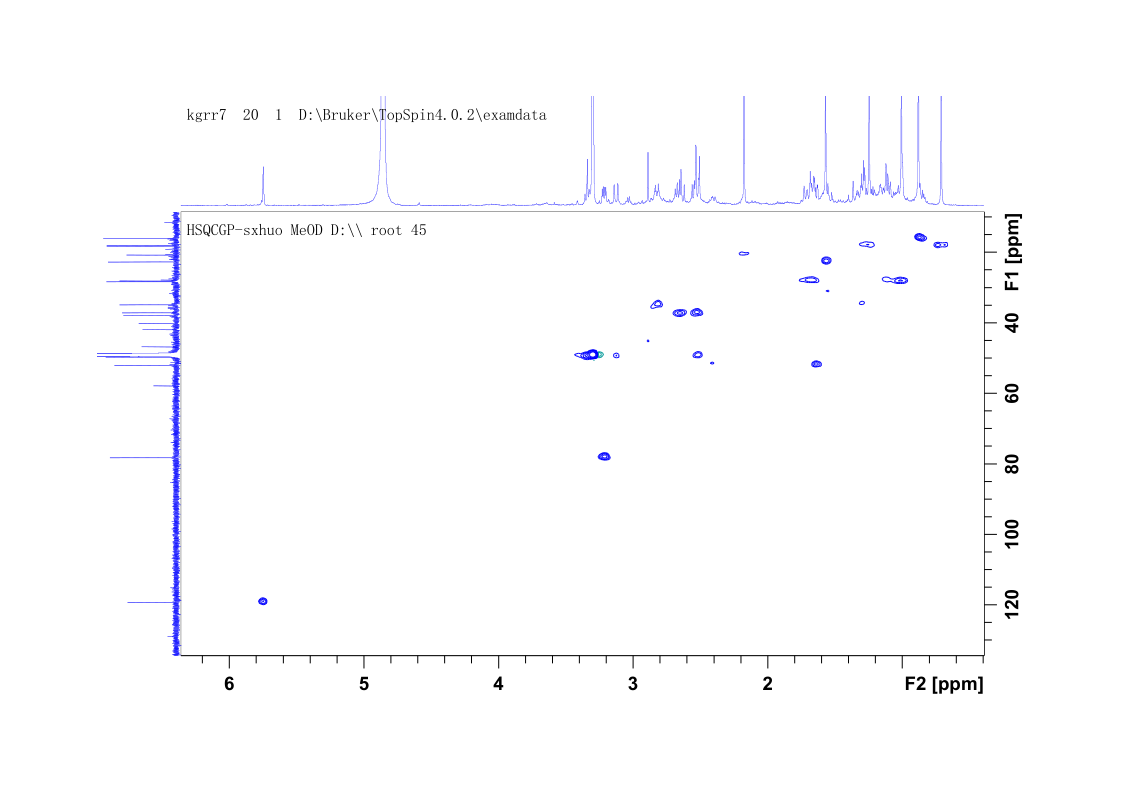


**Figure S15**. HSQC spectrum (600/150 MHz, CD_3_OD) of compound **3**.


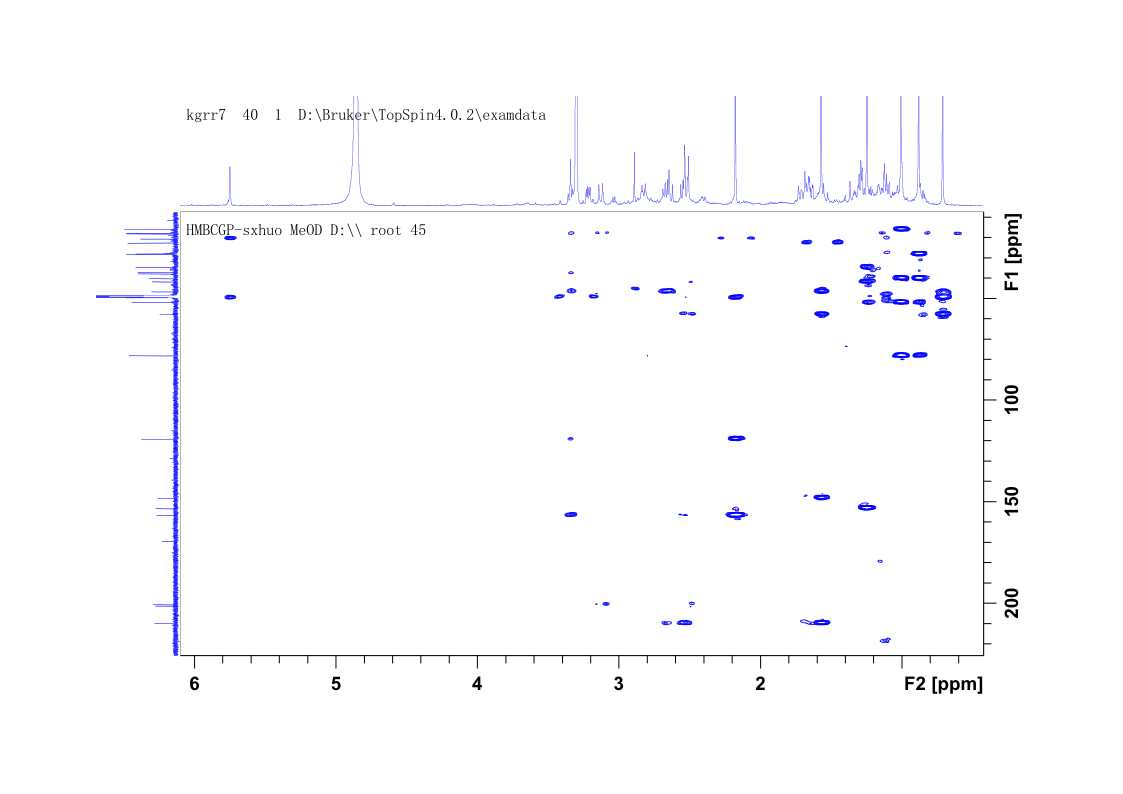


**Figure S16**. HSQC spectrum (600/150 MHz, CD_3_OD) of compound **3**.


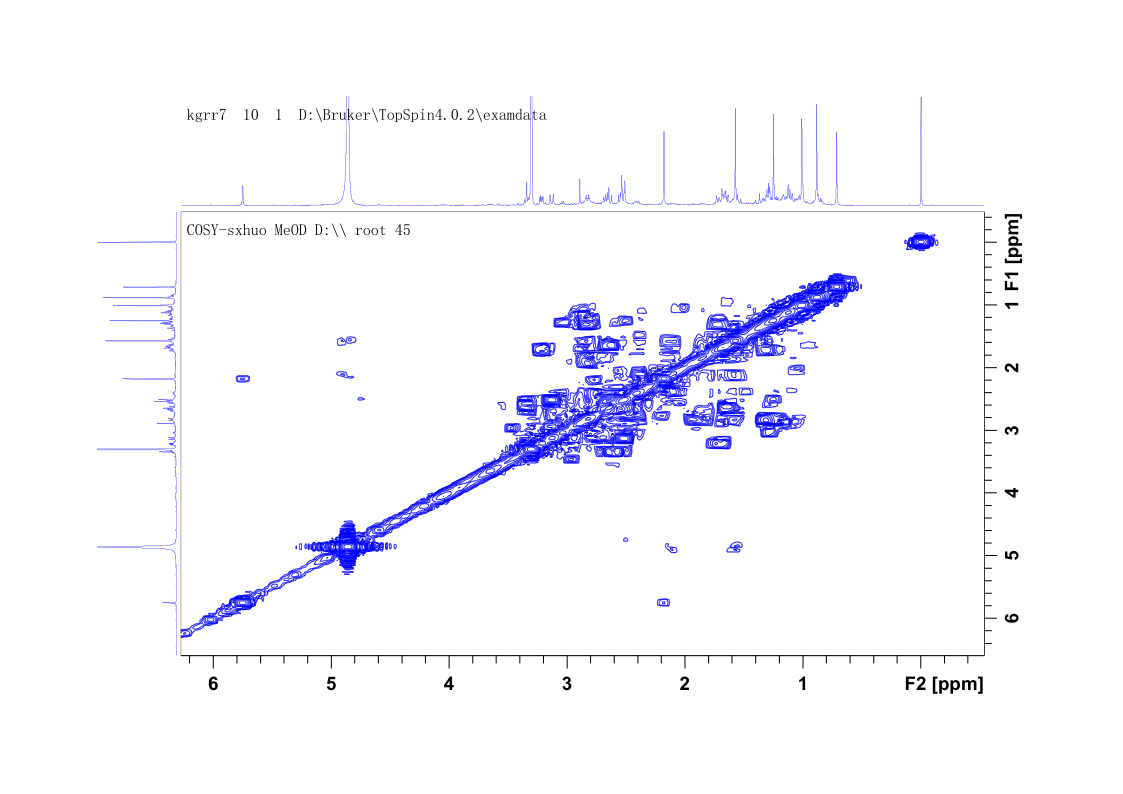


**Figure S17**. ^1^H-^1^H COSY spectrum (600 MHz, CD_3_OD) of compound **3**.


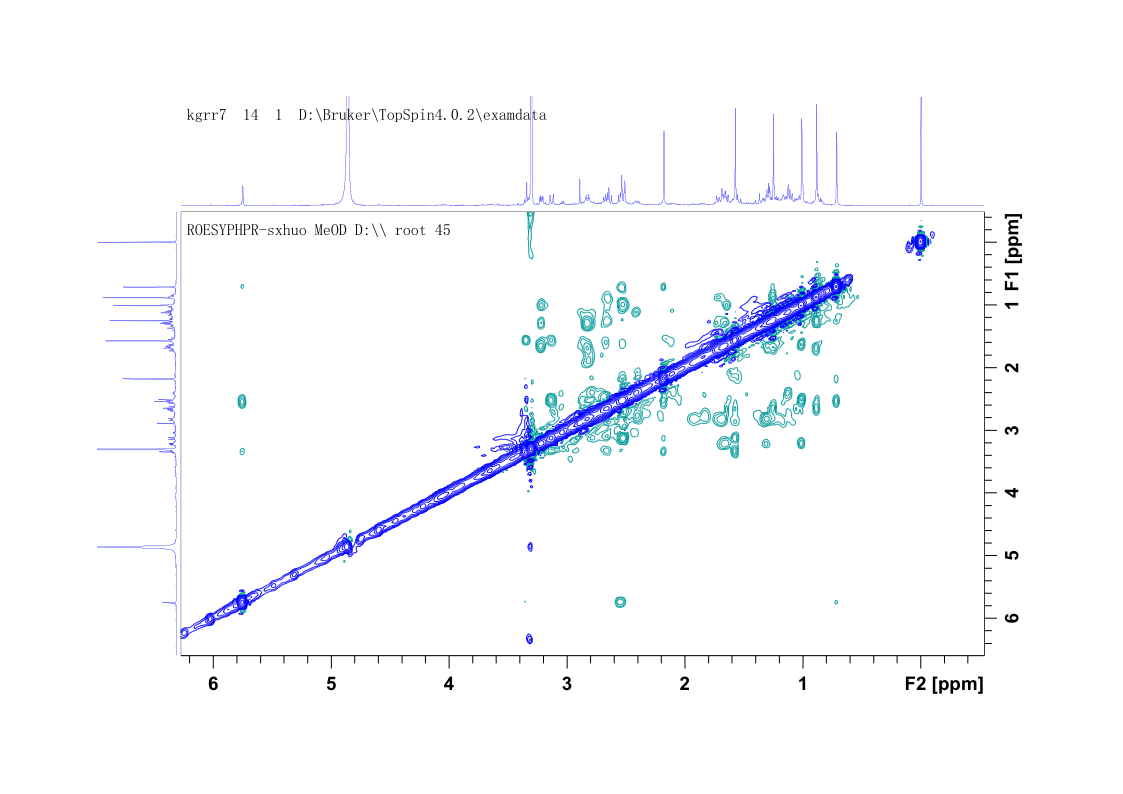


**Figure S18**. ROESY spectrum (600 MHz, CD_3_OD) of compound **3**.


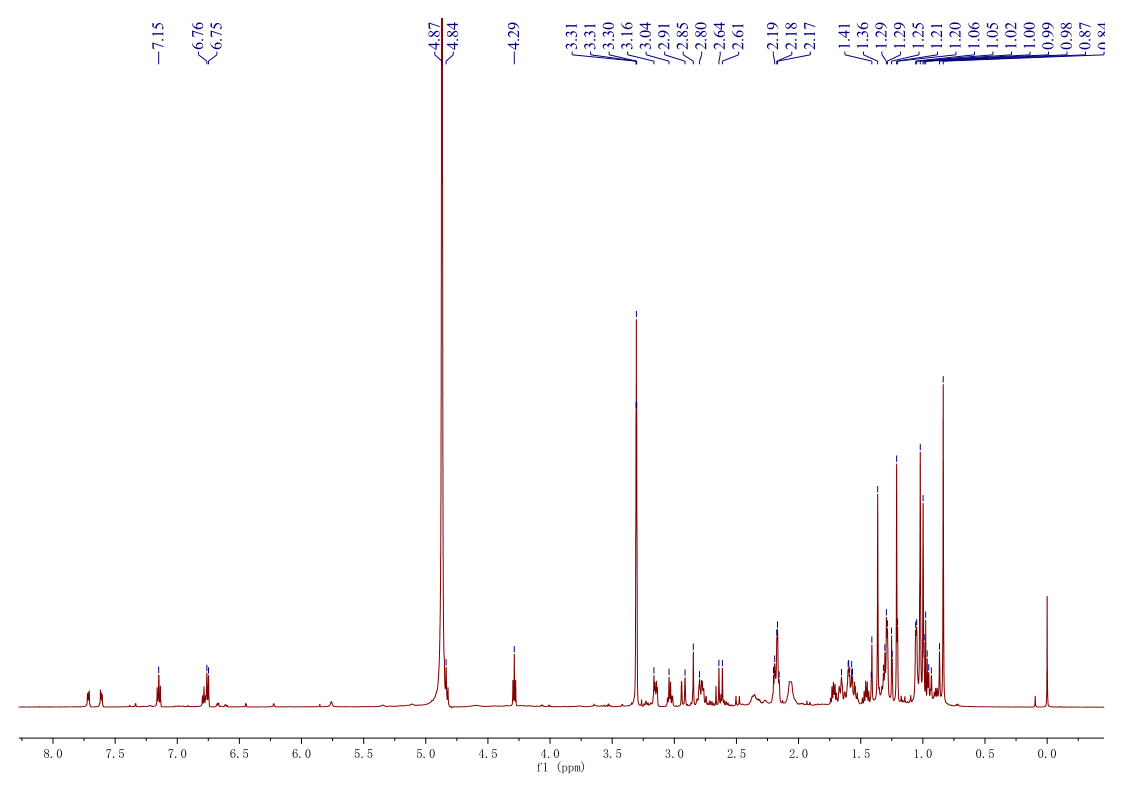


**Figure S19**. ^1^H NMR spectrum (600 MHz, CD_3_OD) of compound **4**.


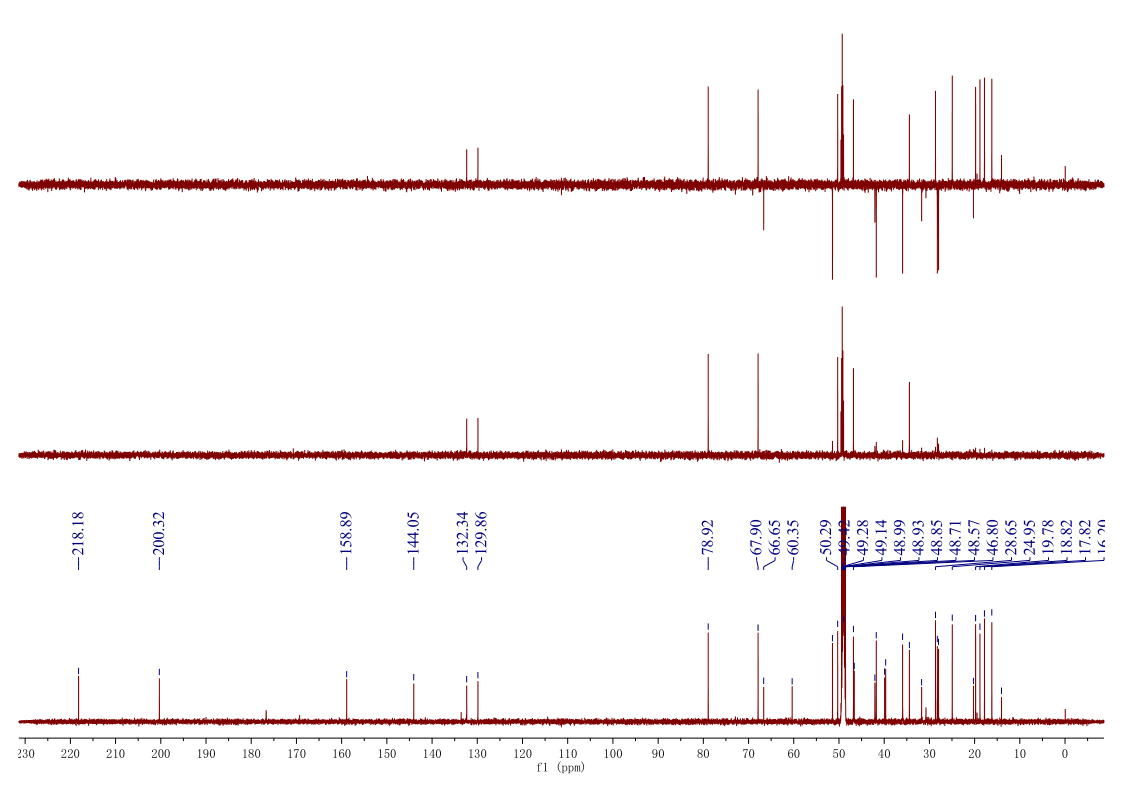


**Figure S20**. ^13^C NMR spectrum (150 MHz, CD_3_OD) of compound **4**.


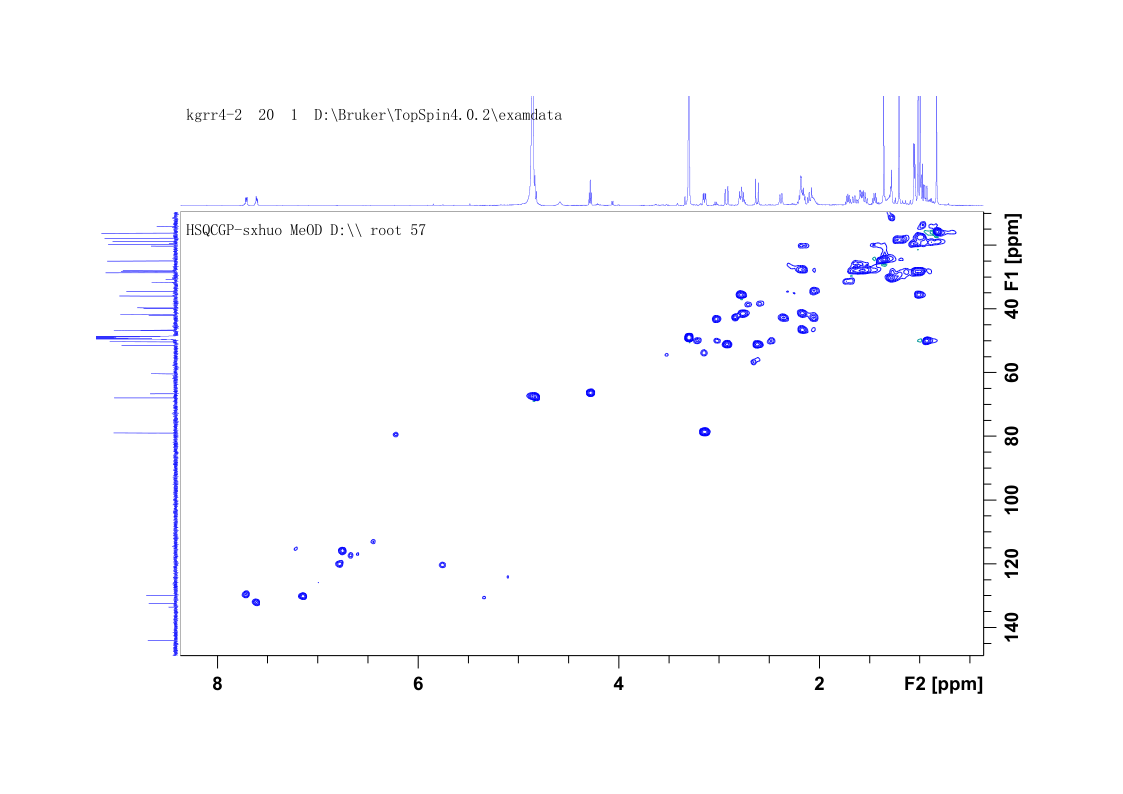


**Figure S21**. HSQC spectrum (600/150 MHz, CD_3_OD) of compound **4**.


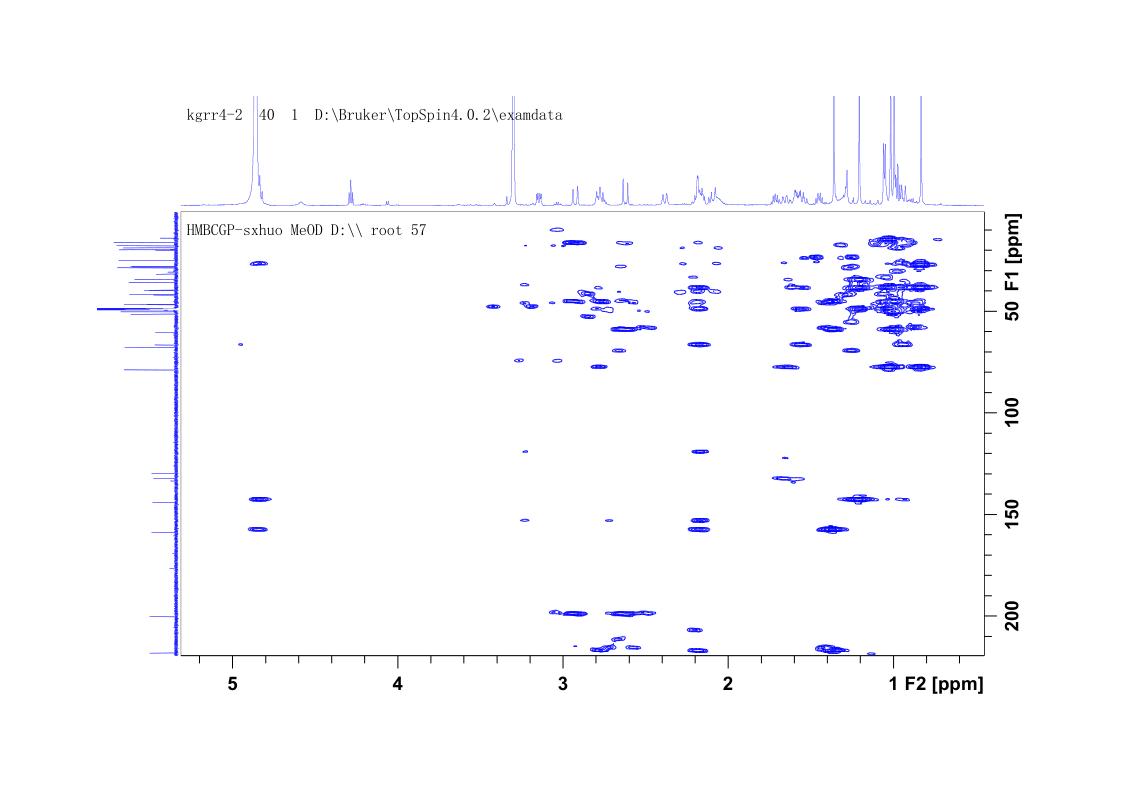


**Figure S22**. HMBC spectrum (600/150 MHz, CD_3_OD) of compound **4**.


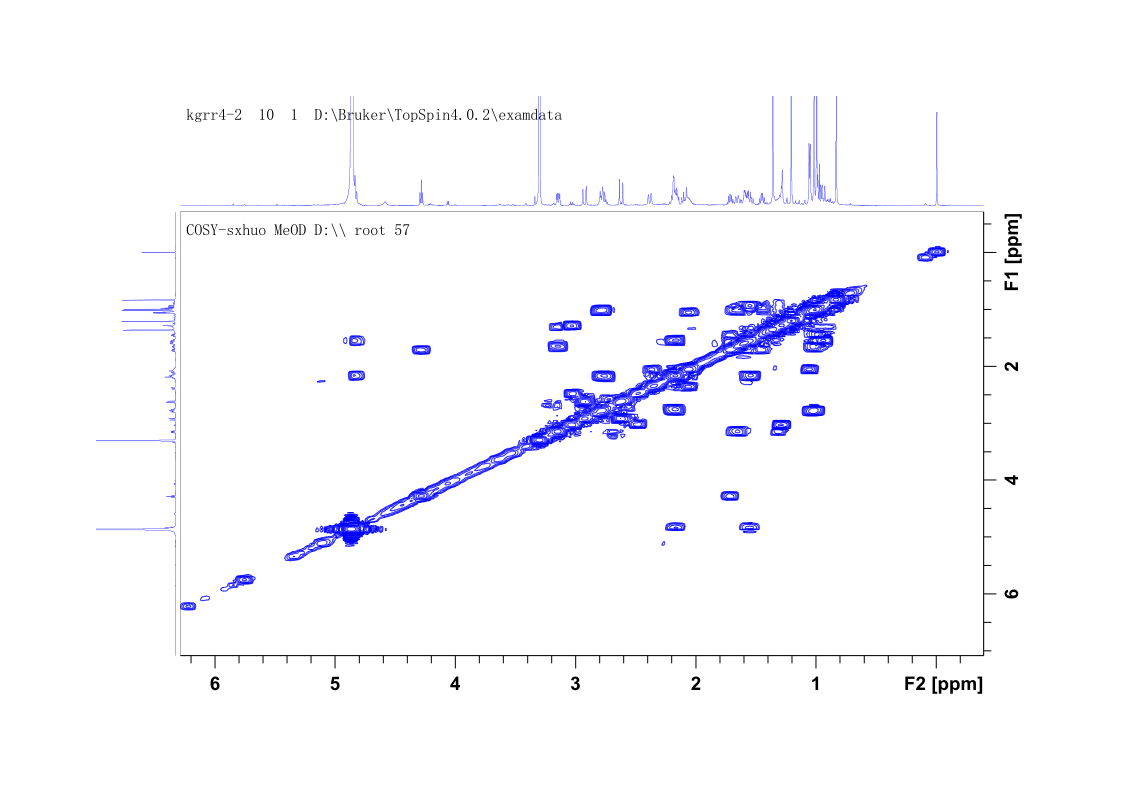


**Figure S23**. ^1^H-^1^H COSY spectrum (600 MHz, CD_3_OD) of compound **4**.


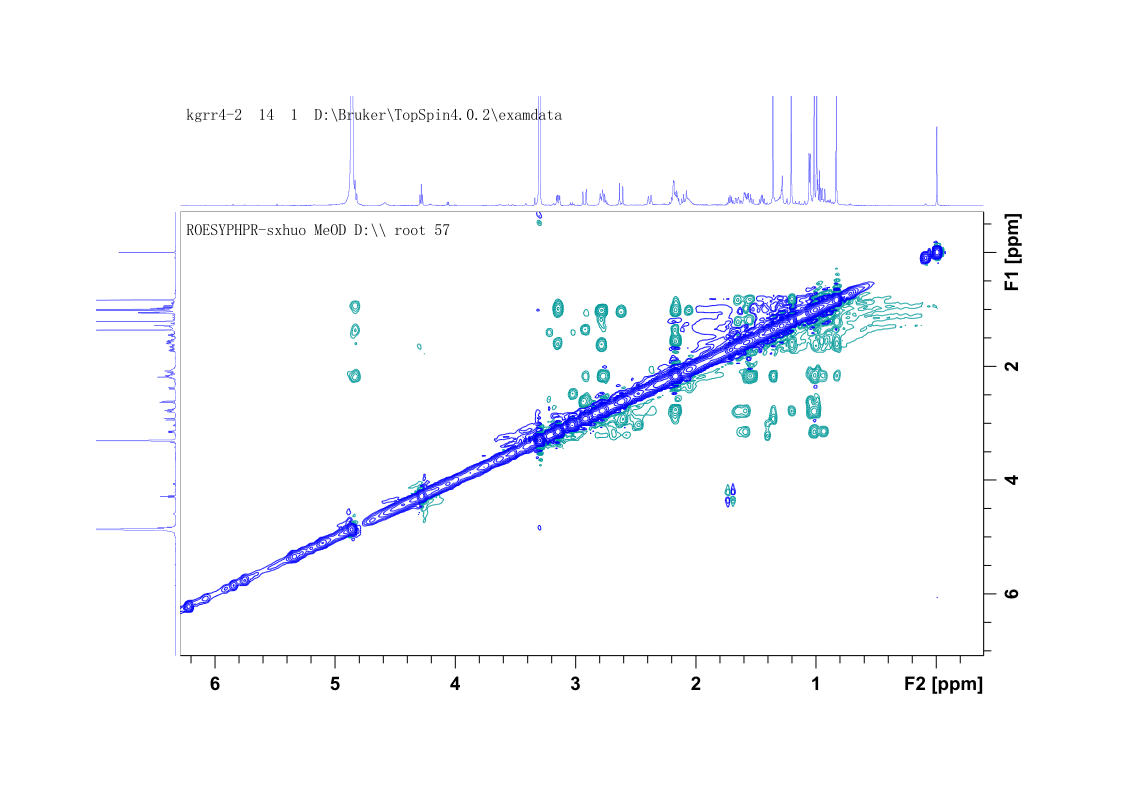


**Figure S24**. ROESY spectrum (600 MHz, CD_3_OD) of compound **4**.


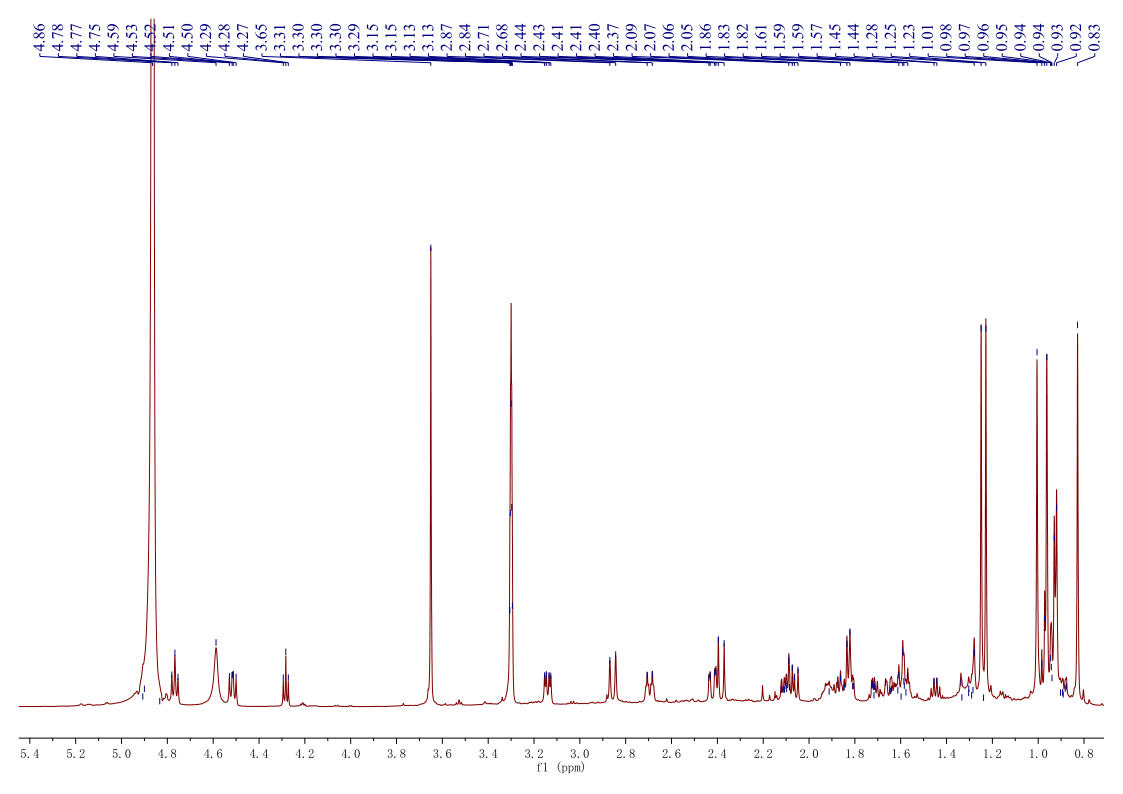


**Figure S25**. ^1^H NMR spectrum (600 MHz, CD_3_OD) of compound **5**.


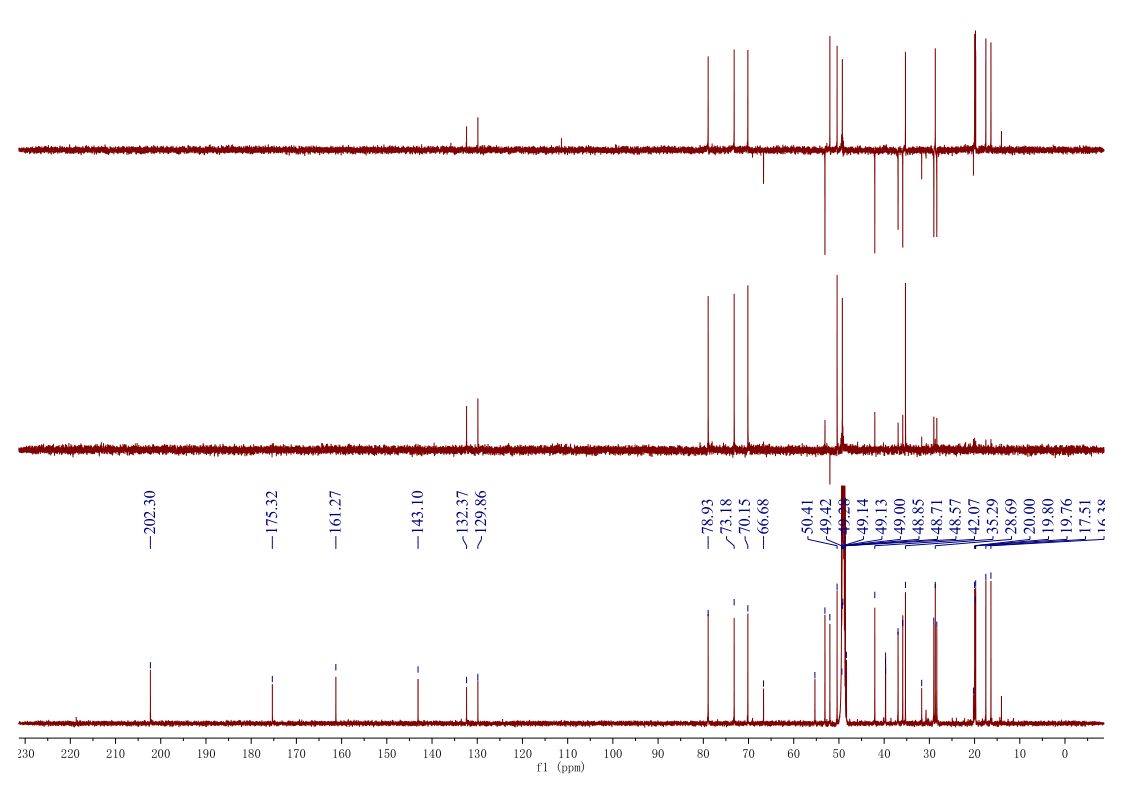


**Figure S26**. ^13^C NMR spectrum (150 MHz, CD_3_OD) of compound **5**.


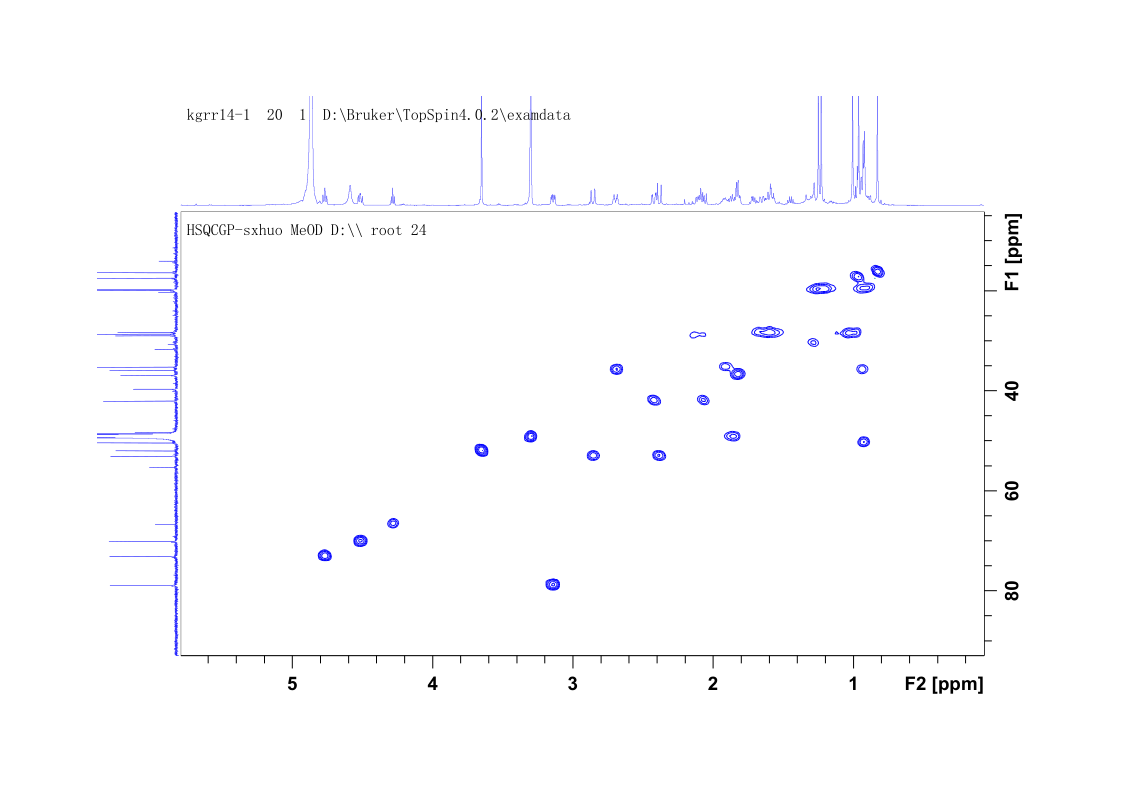


**Figure S27**. HSQC spectrum (600/150 MHz, CD_3_OD) of compound **5**.


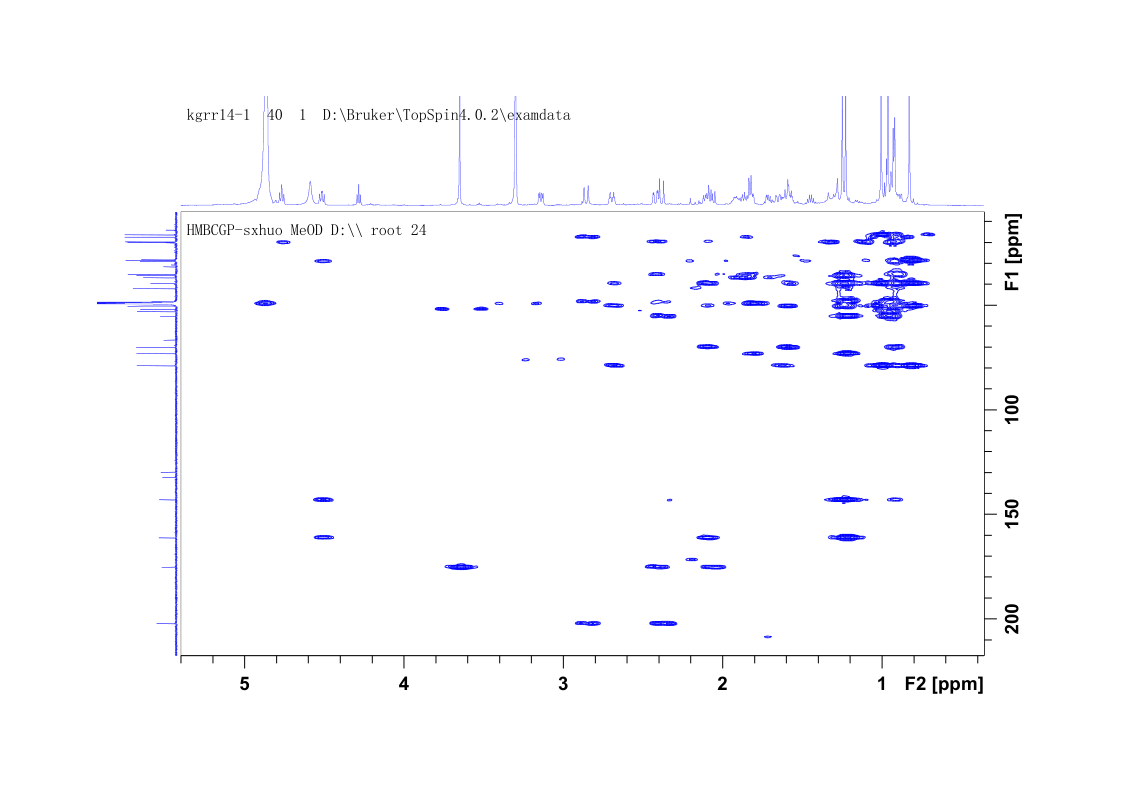


**Figure S28**. HMBC spectrum (600/150 MHz, CD_3_OD) of compound **5**.


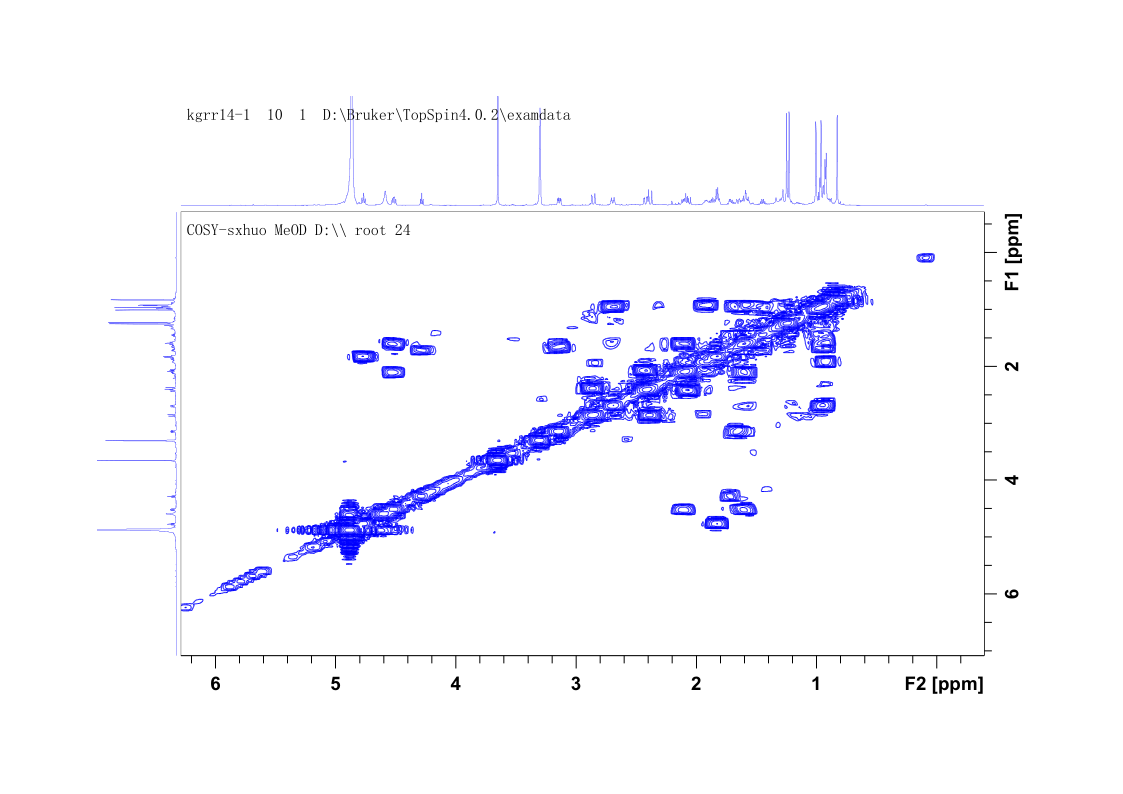


**Figure S29**. ^1^H-^1^H COSY spectrum (600 MHz, CD_3_OD) of compound **5**.


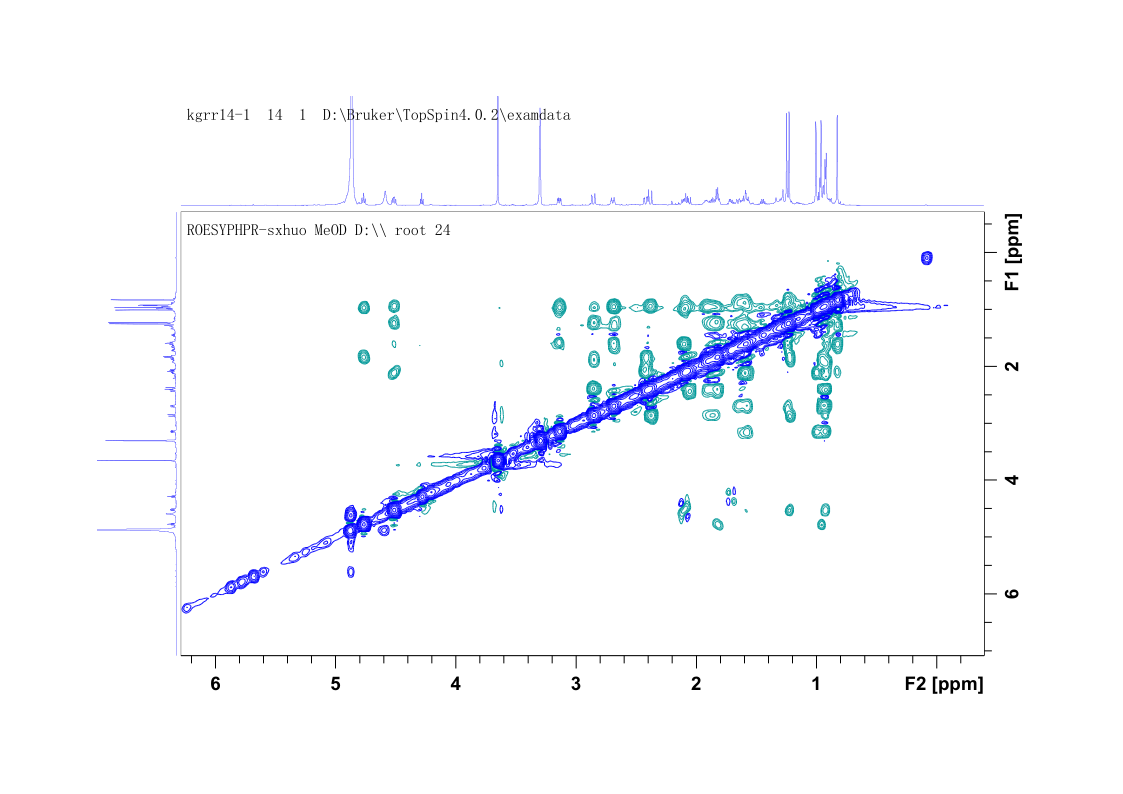


**Figure S30**. ROESY spectrum (600 MHz, CD_3_OD) of compound **5**.


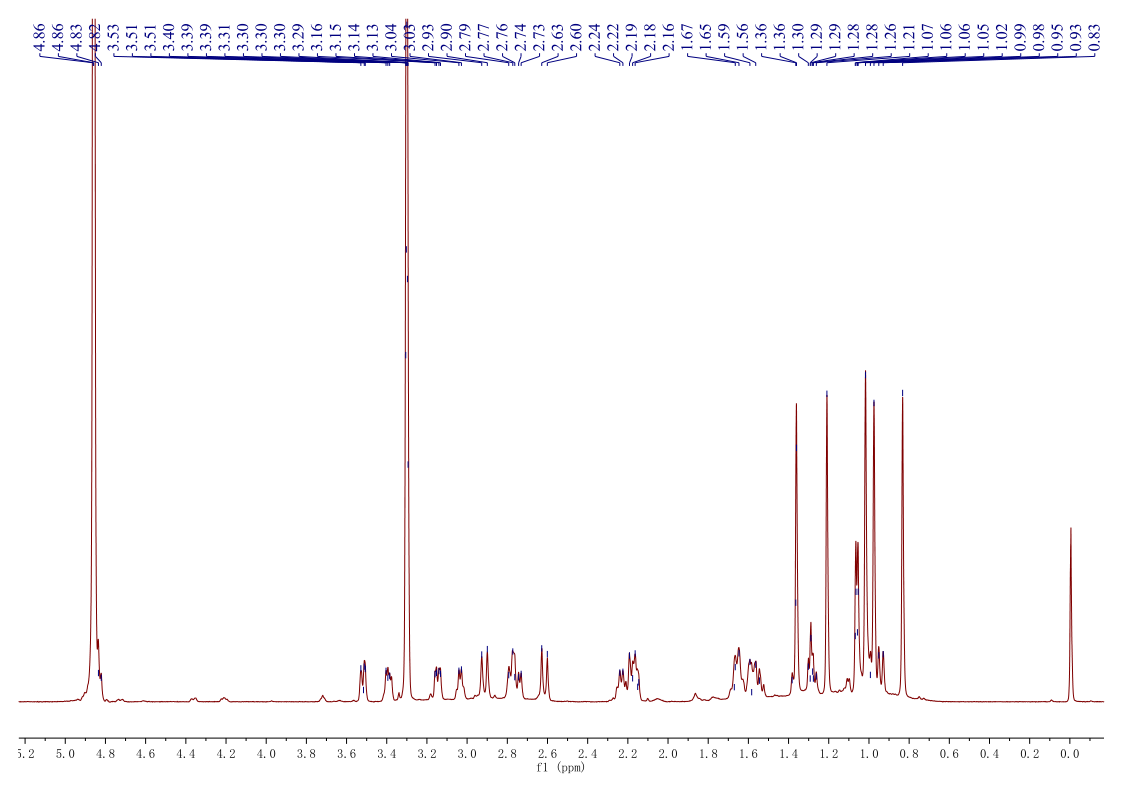


**Figure S31**. ^1^H NMR spectrum (600 MHz, CD_3_OD) of compound **6**.


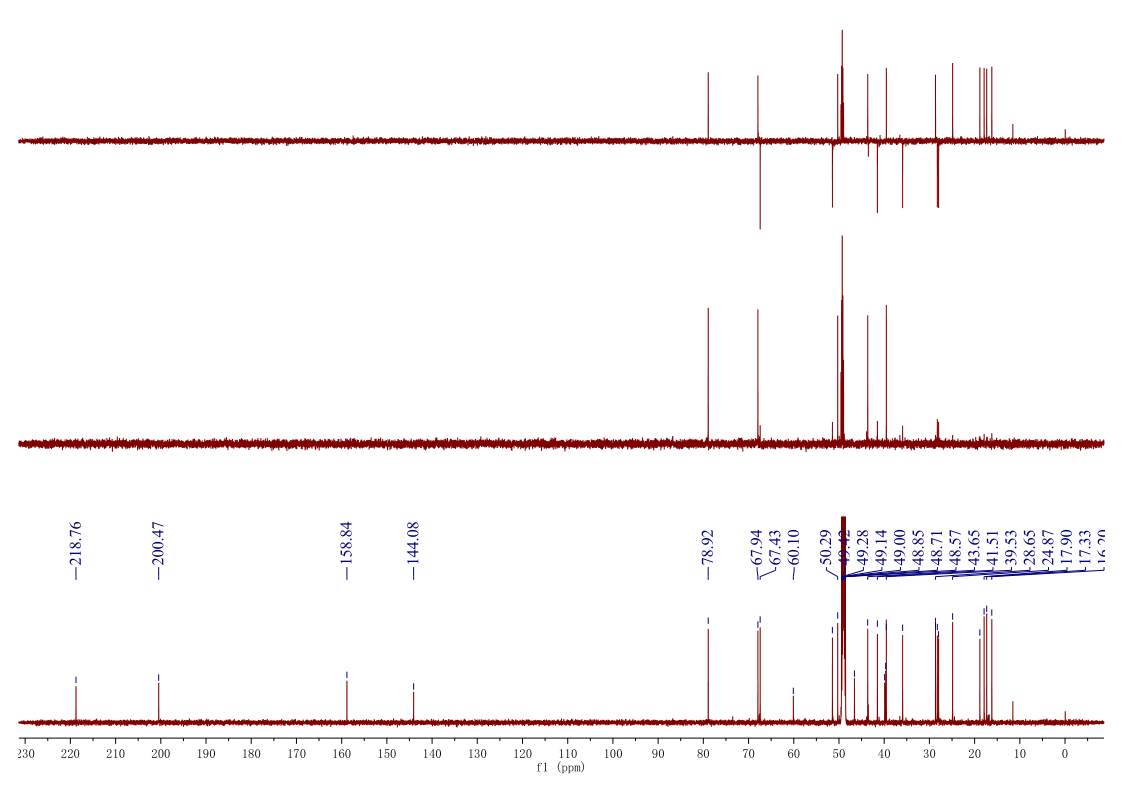


**Figure S32**. ^13^C NMR spectrum (150 MHz, CD_3_OD) of compound **6**.


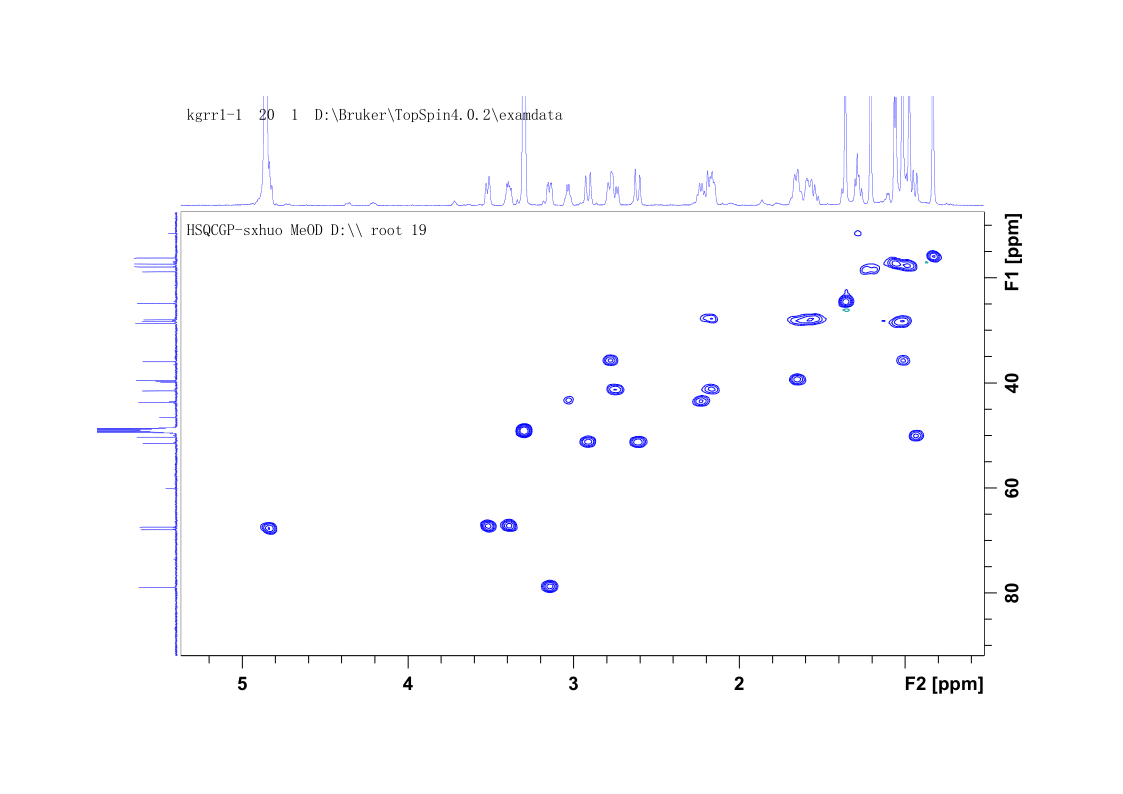


**Figure S33**. HSQC spectrum (600/150 MHz, CD_3_OD) of compound **6**.


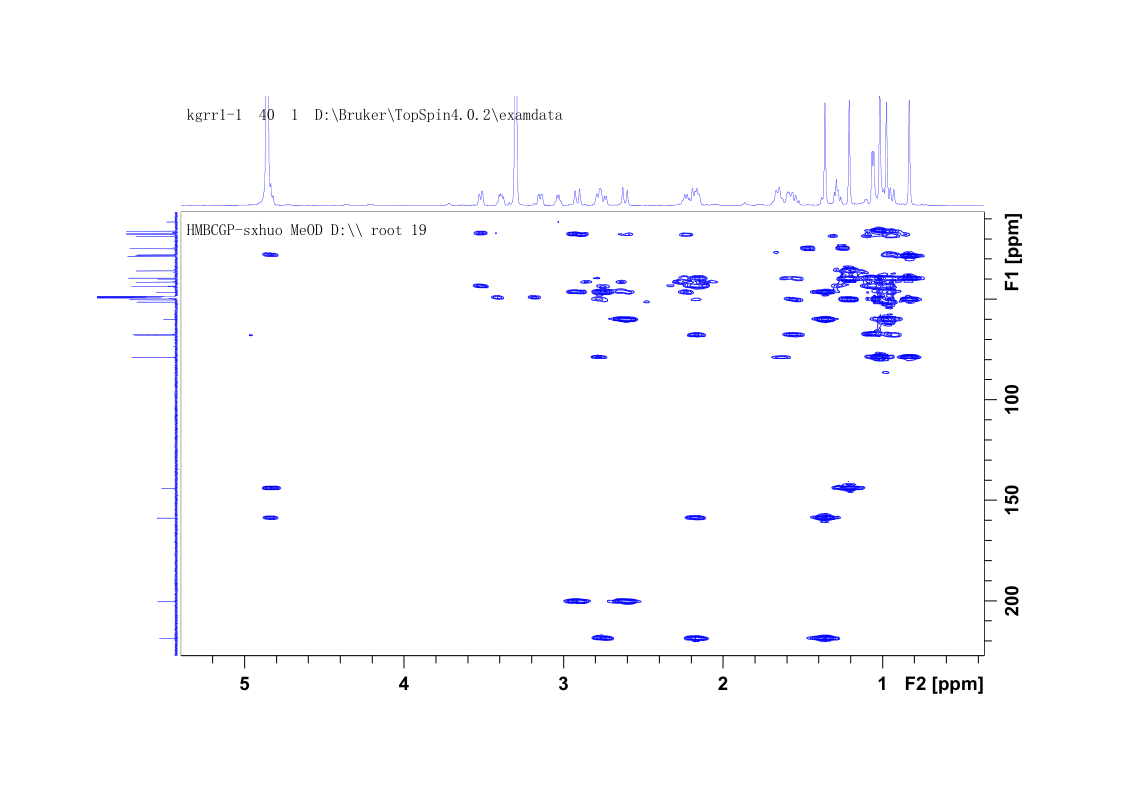


**Figure S34**. HMBC spectrum (600/150 MHz, CD_3_OD) of compound **6**.


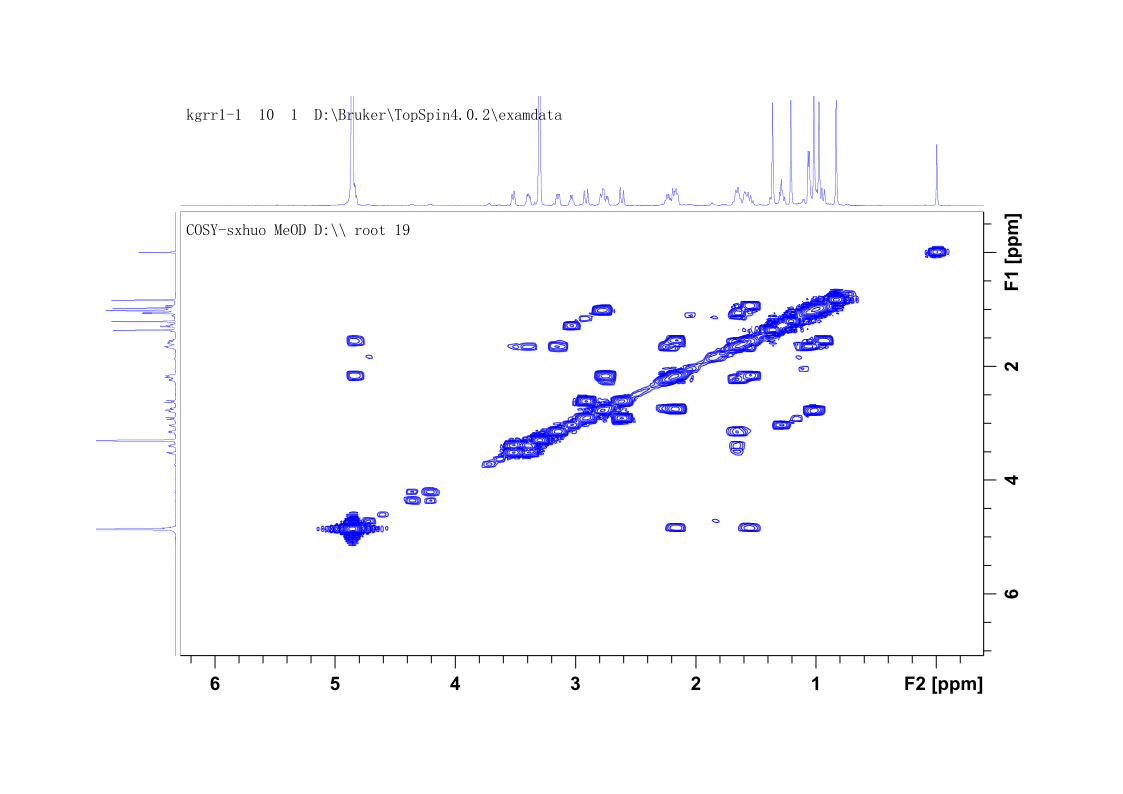


**Figure S35**. ^1^H-^1^H COSY spectrum (600 MHz, CD_3_OD) of compound **6**.


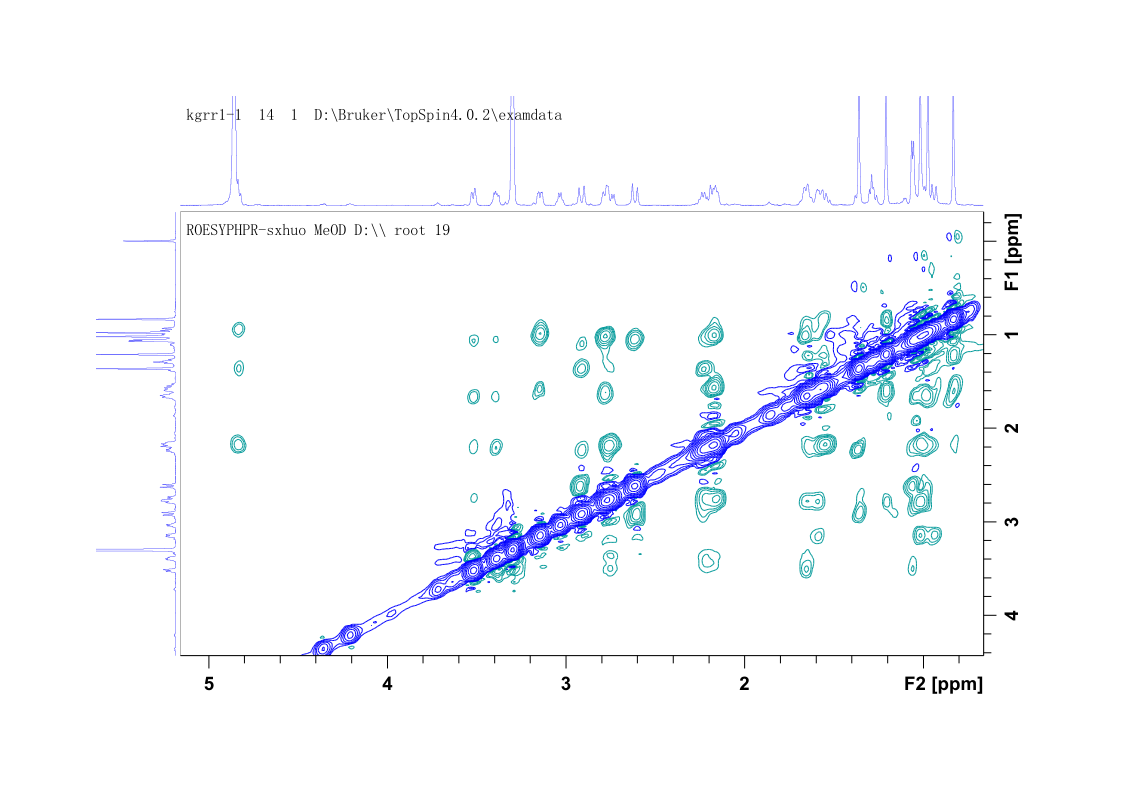


**Figure S36**. ROESY spectrum (600 MHz, CD_3_OD) of compound **6**.


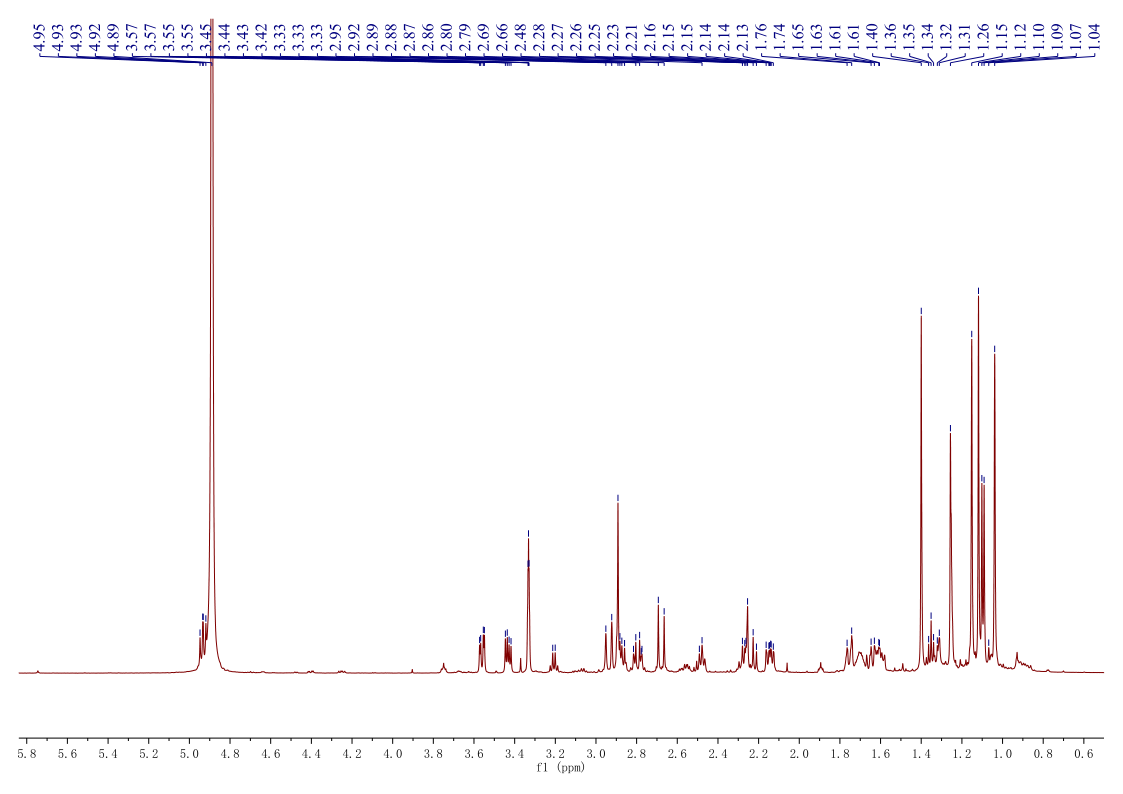


**Figure S37**. ^1^H NMR spectrum (600 MHz, CD_3_OD) of compound **7**.


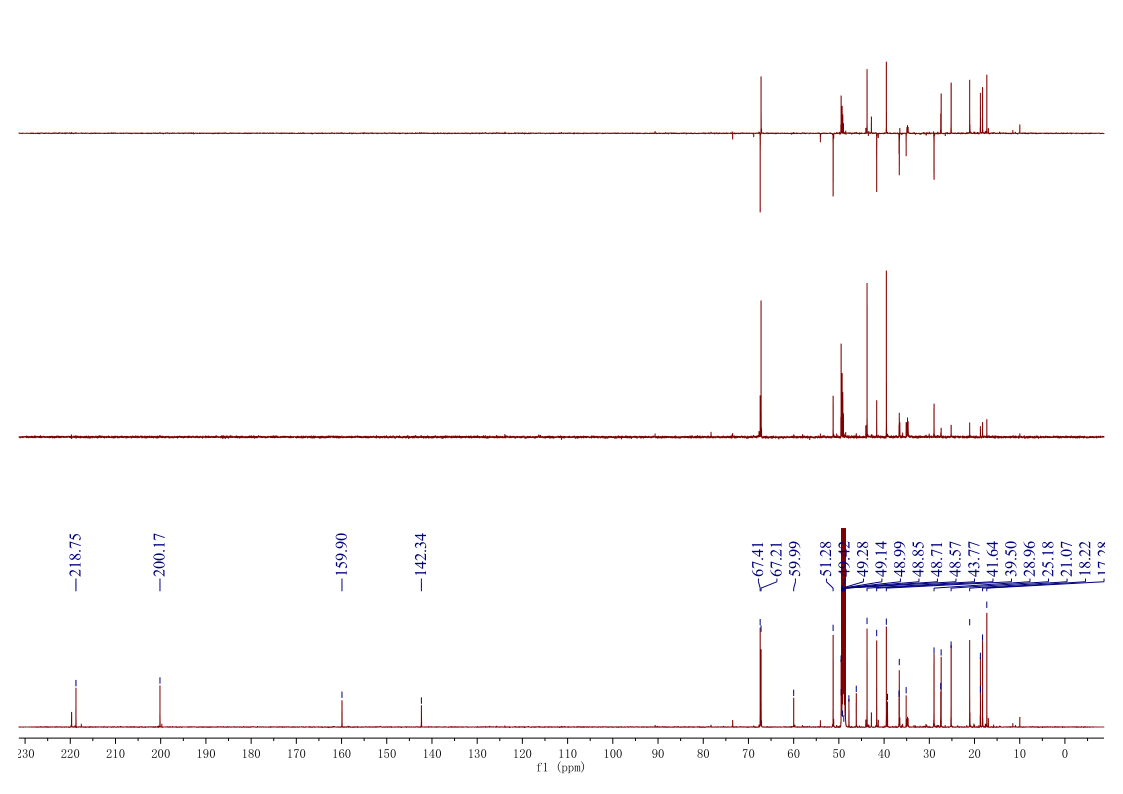


**Figure S38**. ^13^C NMR spectrum (150 MHz, CD_3_OD) of compound **7**.


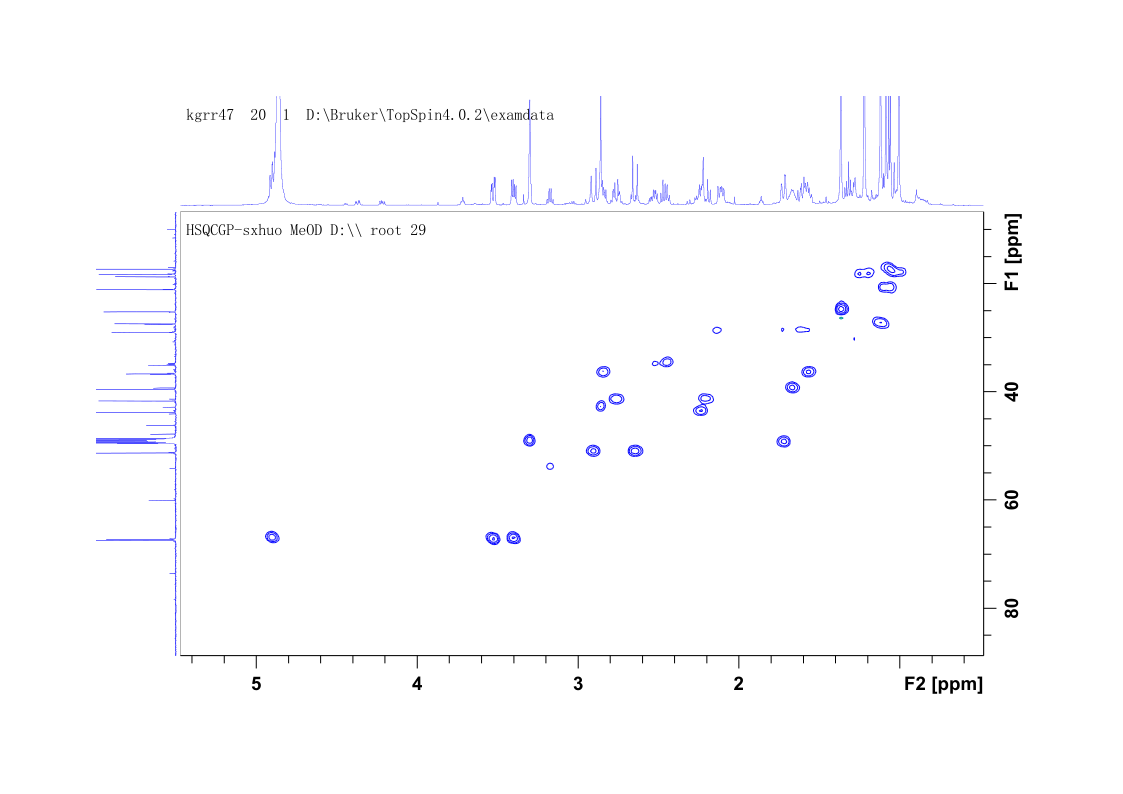


**Figure S39**. HSQC spectrum (600/150 MHz, CD_3_OD) of compound **7**.


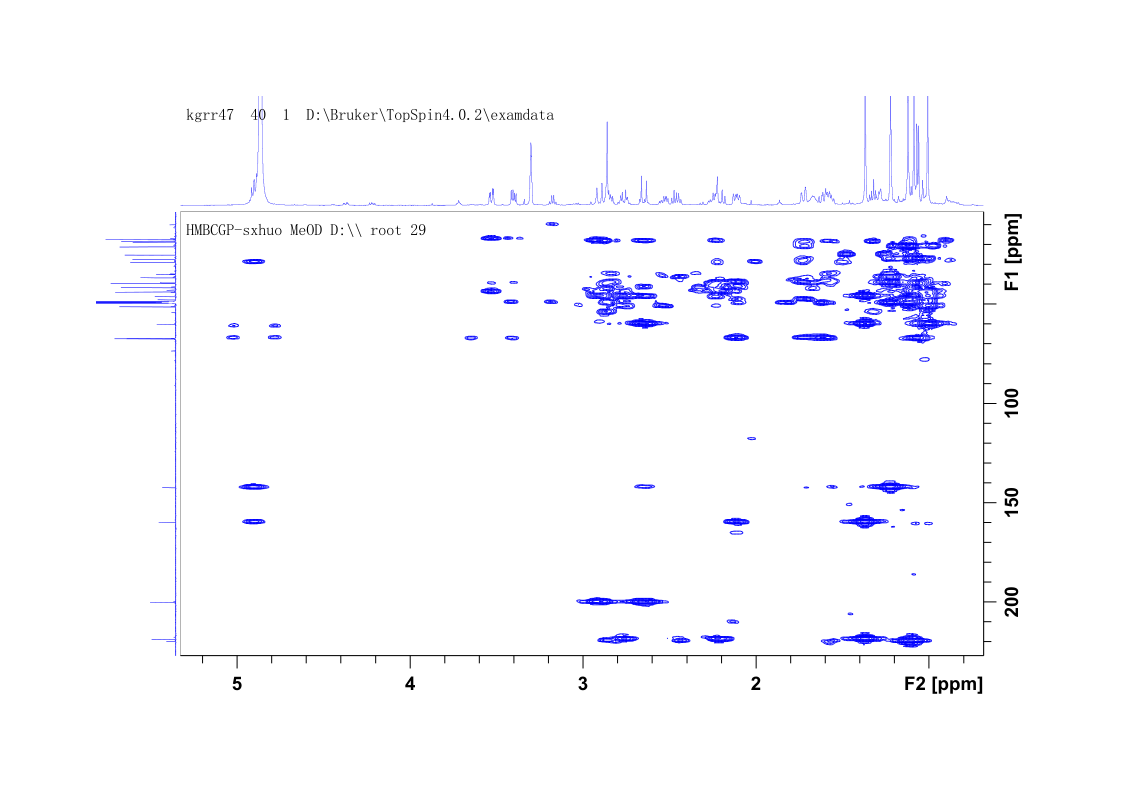


**Figure S40**. HMBC spectrum (600/150 MHz, CD_3_OD) of compound **7**.


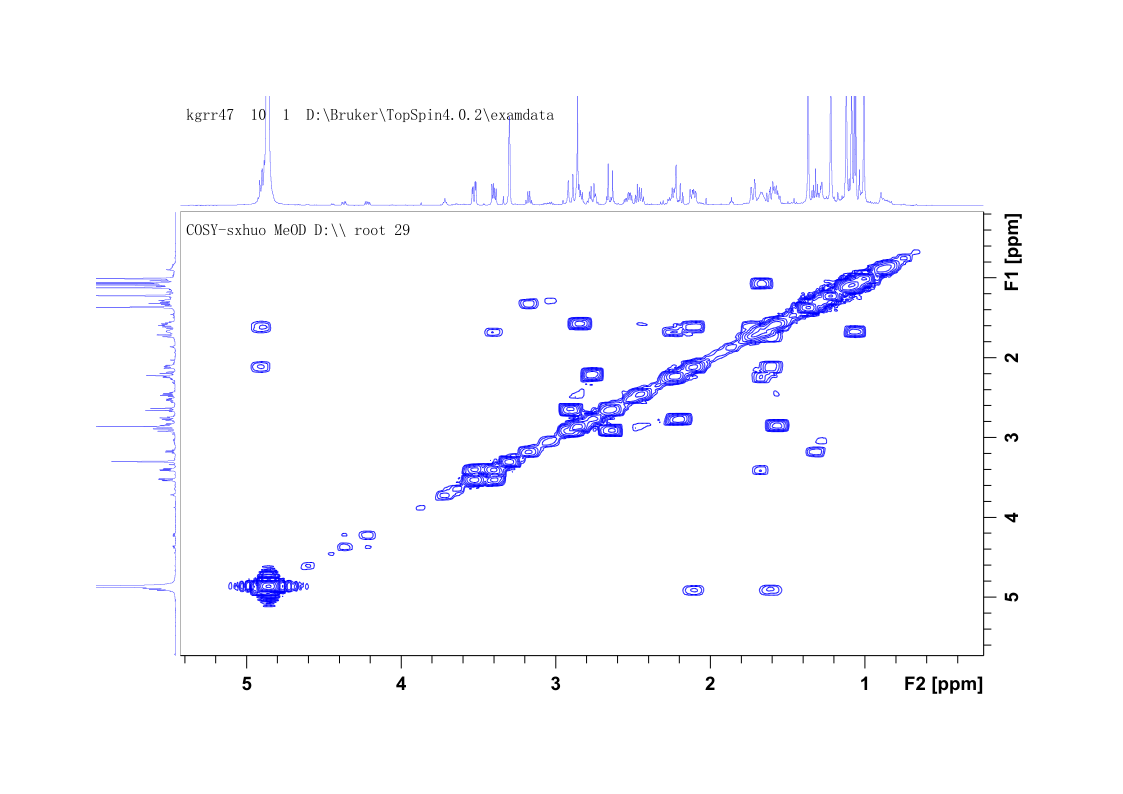


**Figure S41**. ^1^H-^1^H COSY spectrum (600 MHz, CD_3_OD) of compound **7**.


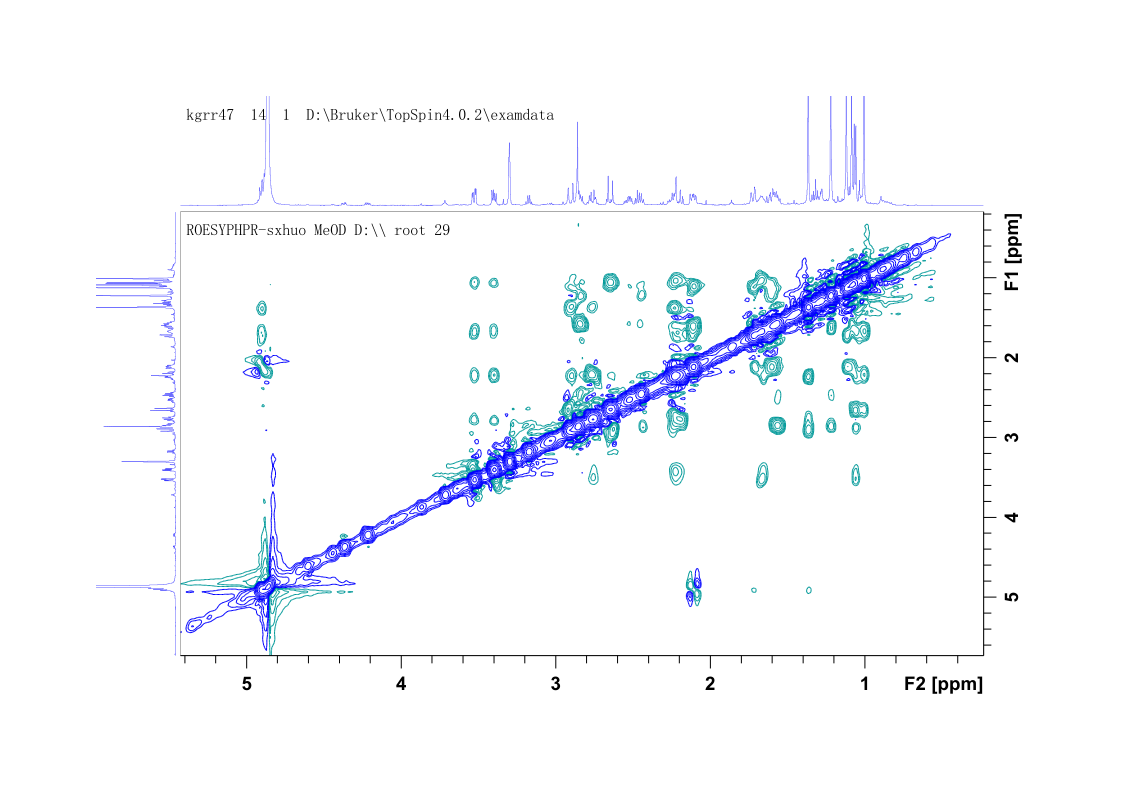


**Figure S42**. ROESY spectrum (600 MHz, CD_3_OD) of compound **7**.


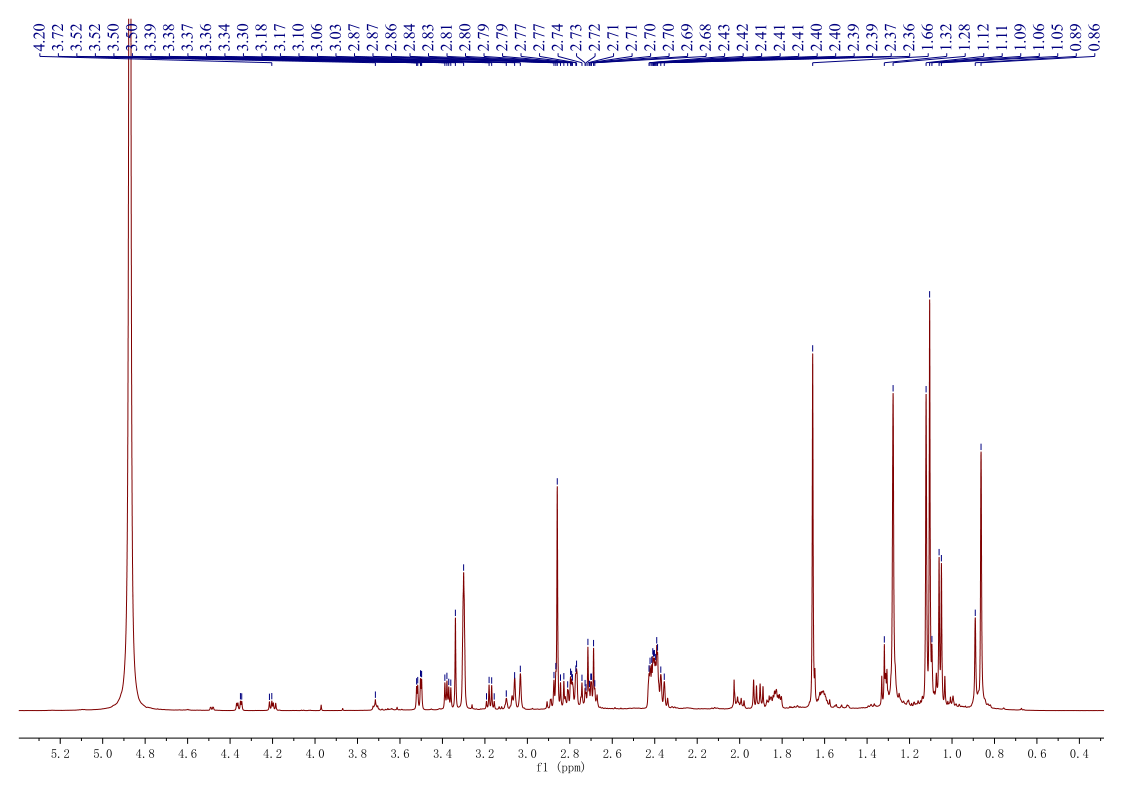


**Figure S43**. ^1^H NMR spectrum (600 MHz, CD_3_OD) of compound **8**.


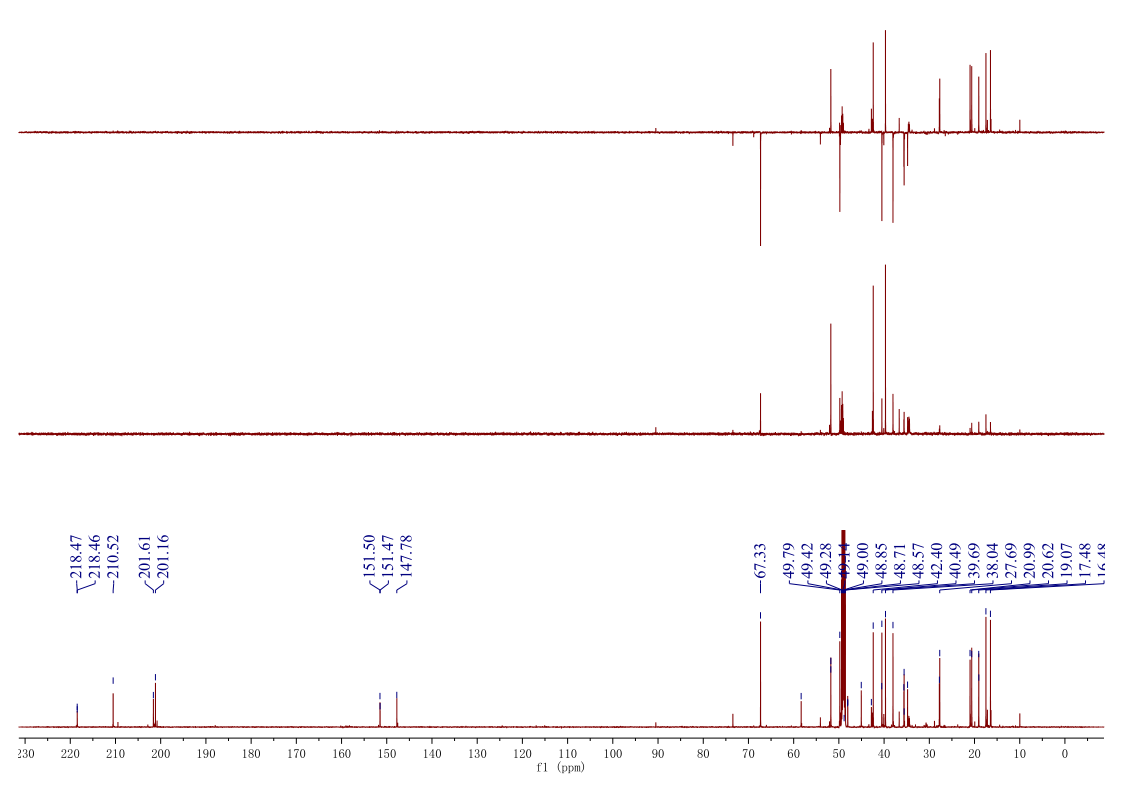


**Figure S44**. ^13^C NMR spectrum (150 MHz, CD_3_OD) of compound **8**.


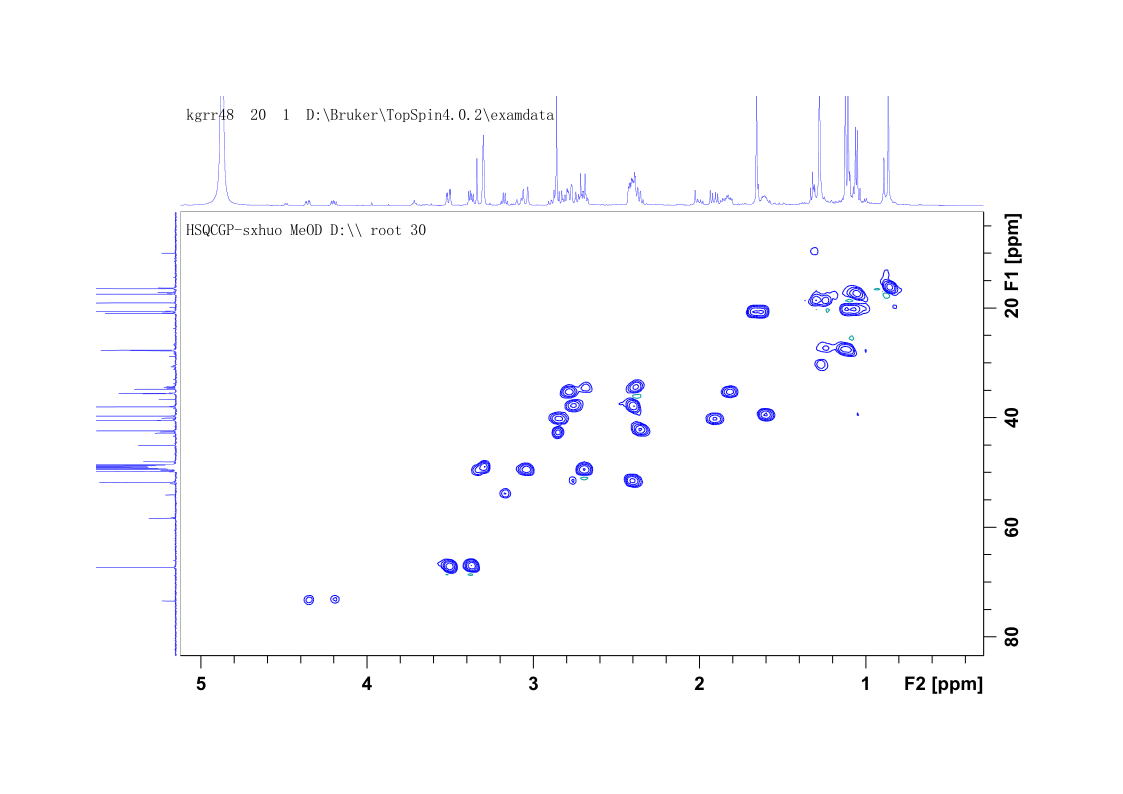


**Figure S45**. HSQC spectrum (600/150 MHz, CD_3_OD) of compound **8**.


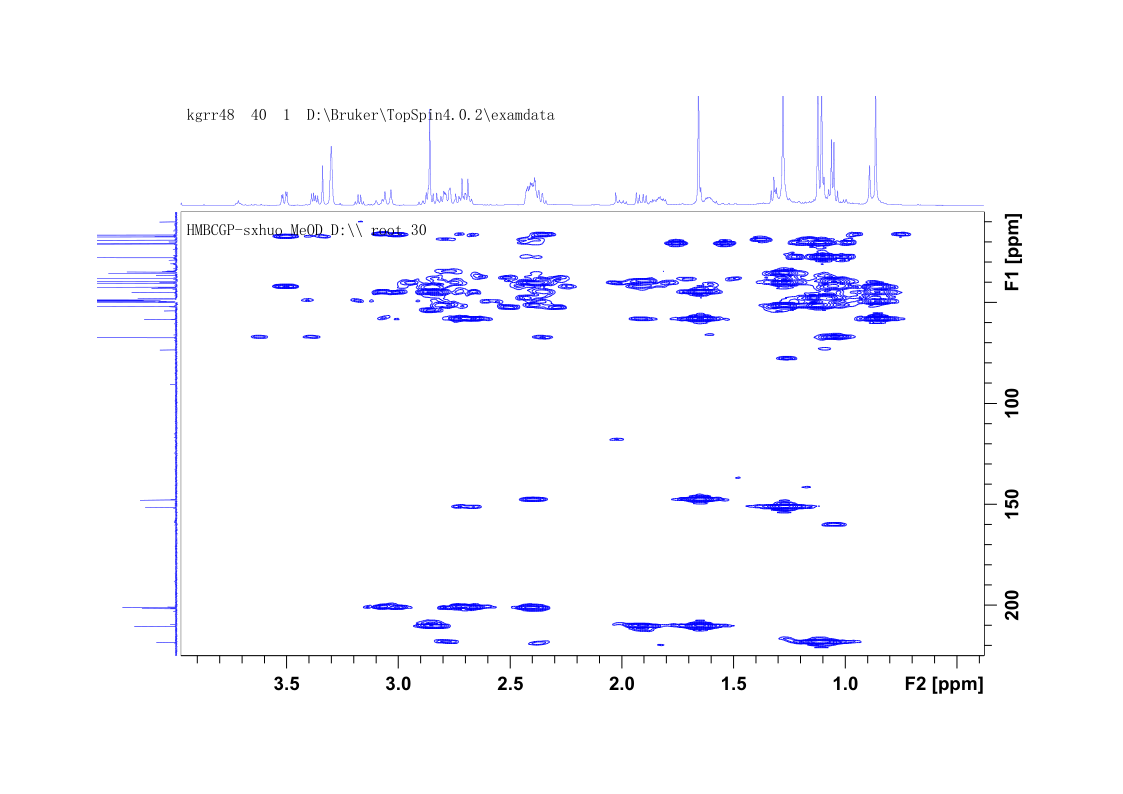


**Figure S46**. HMBC spectrum (600/150 MHz, CD_3_OD) of compound **8**.


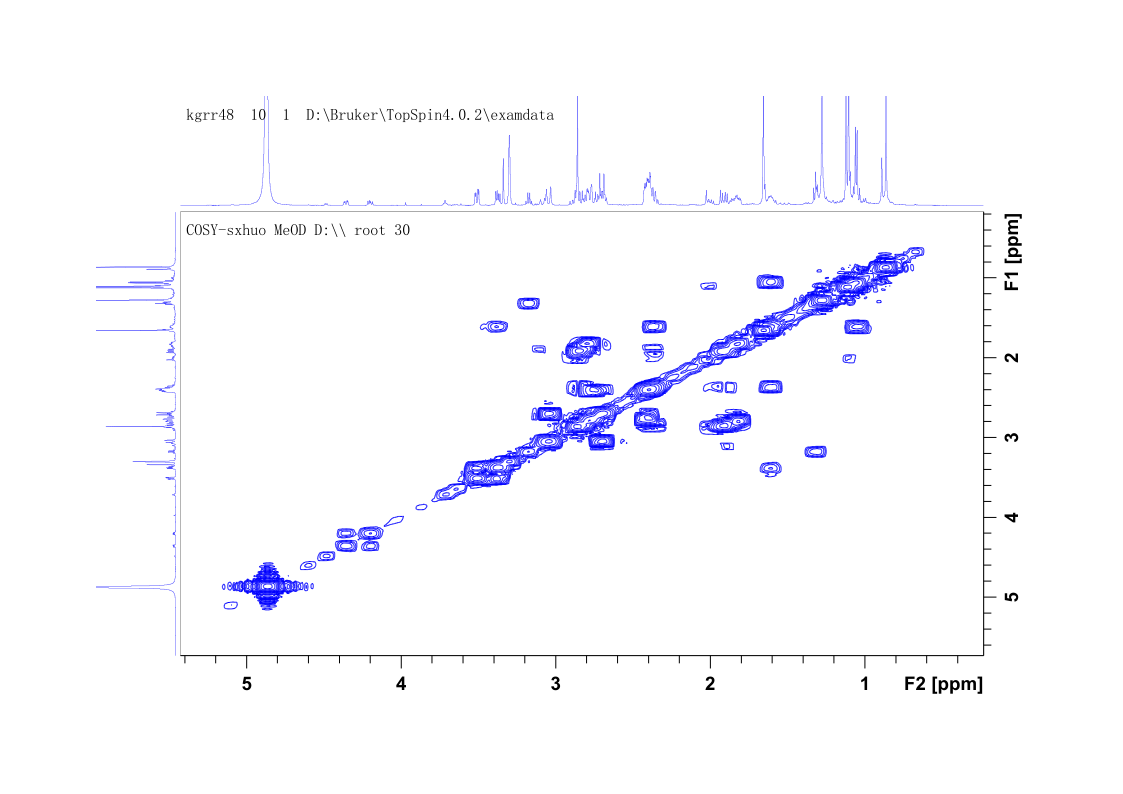


**Figure S47**. ^1^H-^1^H COSY spectrum (600 MHz, CD_3_OD) of compound **8**.


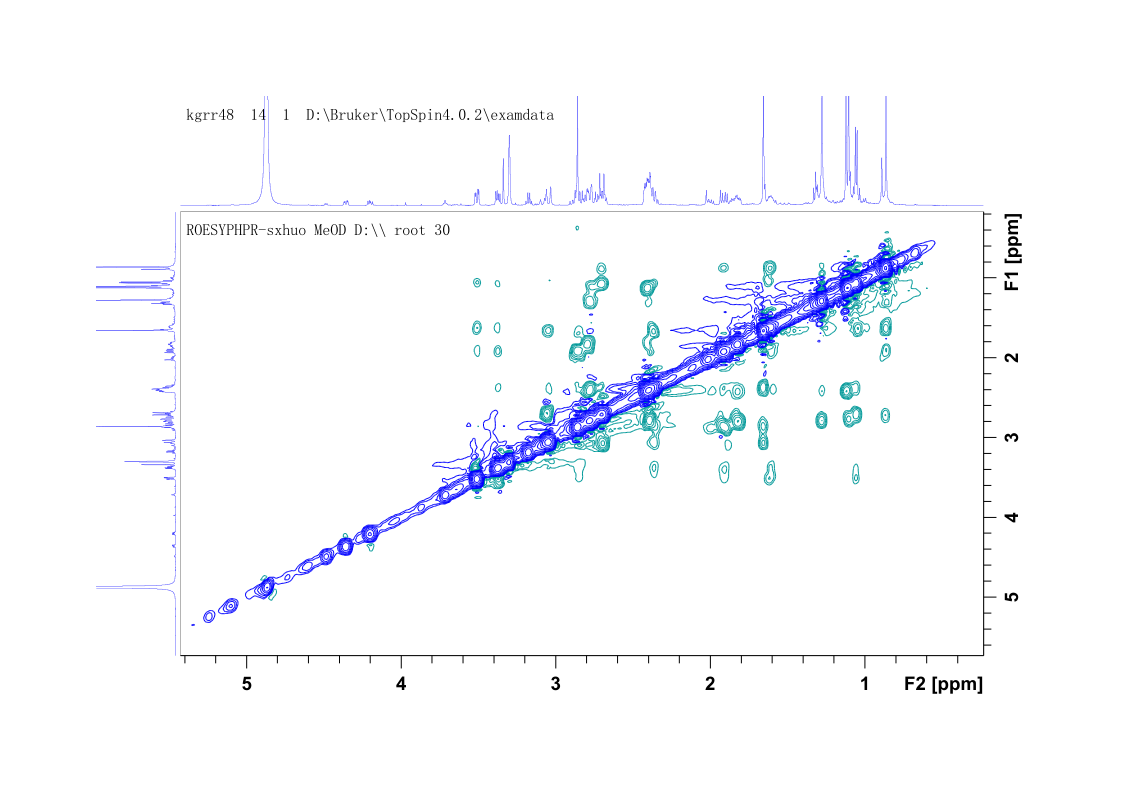


**Figure S48**. ROESY spectrum (600 MHz, CD_3_OD) of compound **8**.


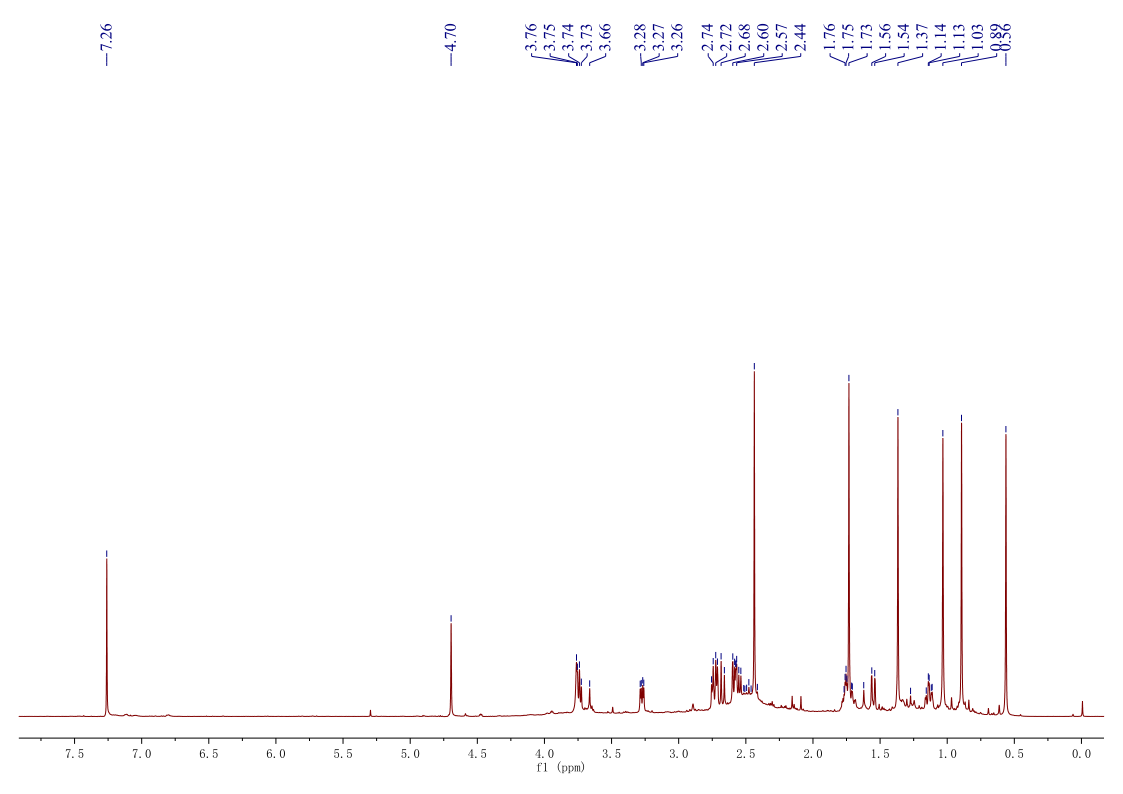


**Figure S49**. ^1^H NMR spectrum (600 MHz, CDCl_3_) of compound **9**.


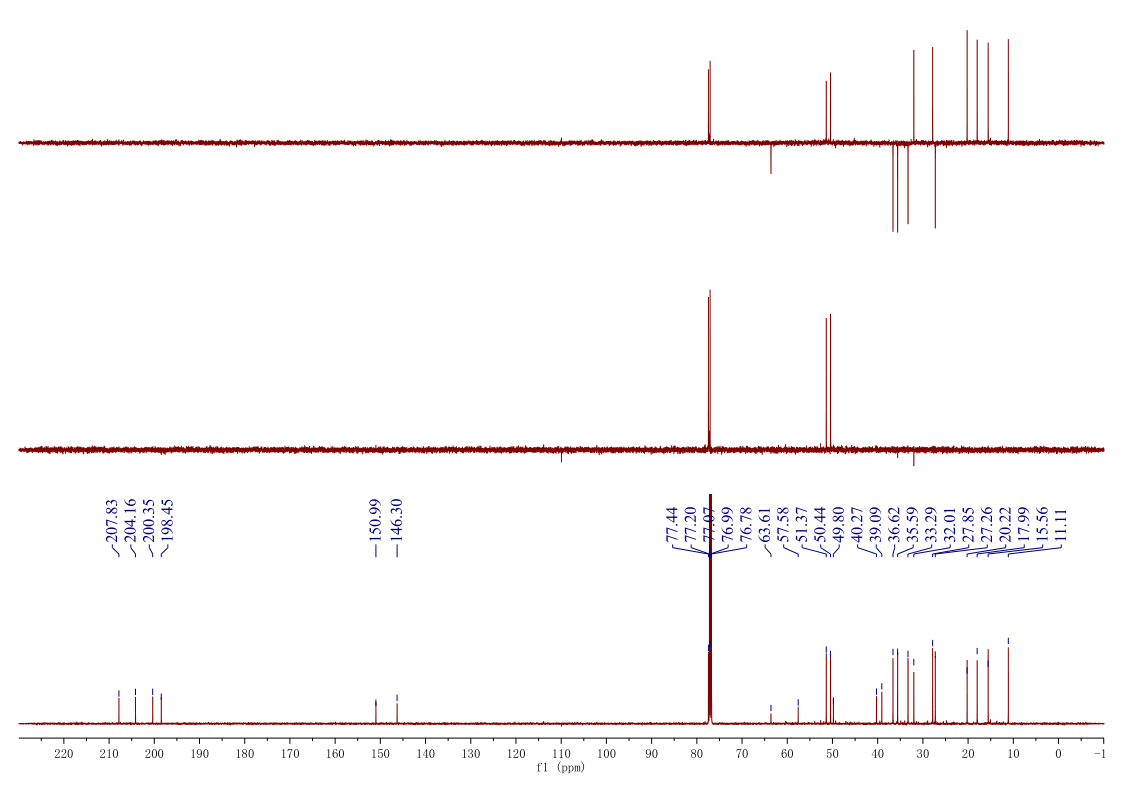


**Figure S50**. ^13^C NMR spectrum (150 MHz, CDCl_3_) of compound **9**.


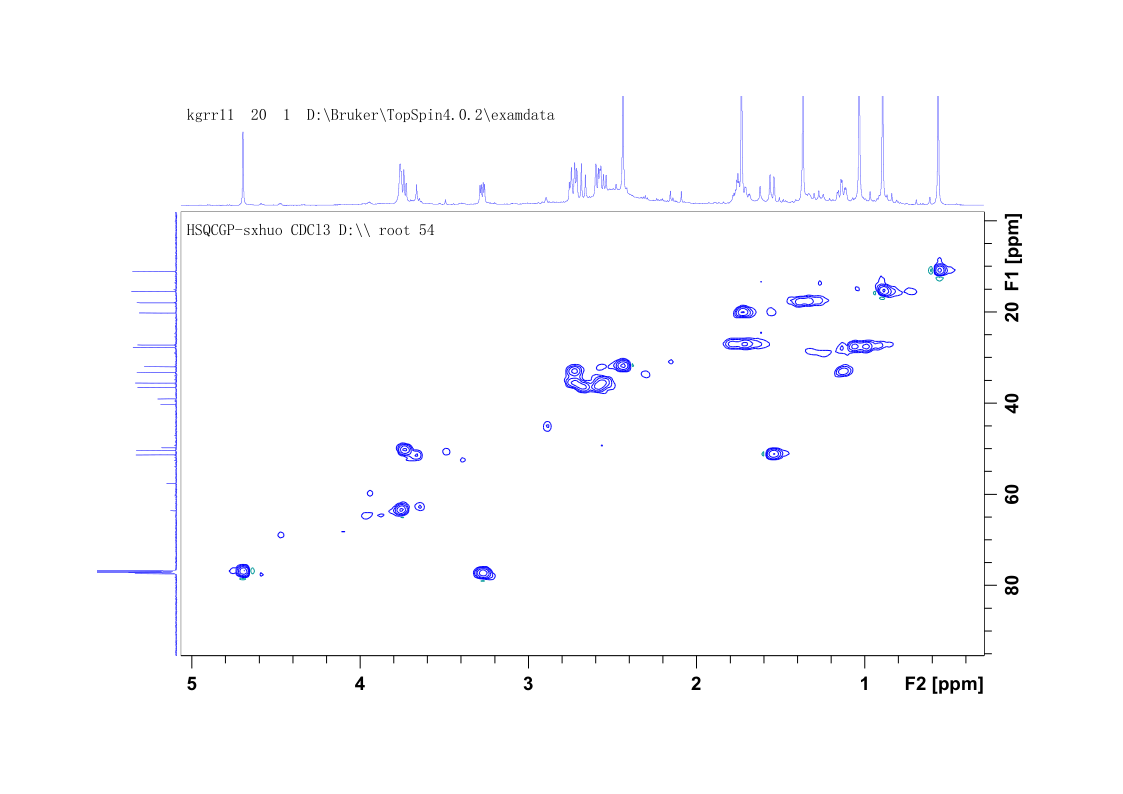


**Figure S51**. HSQC spectrum (600/150 MHz, CDCl_3_) of compound **9**.


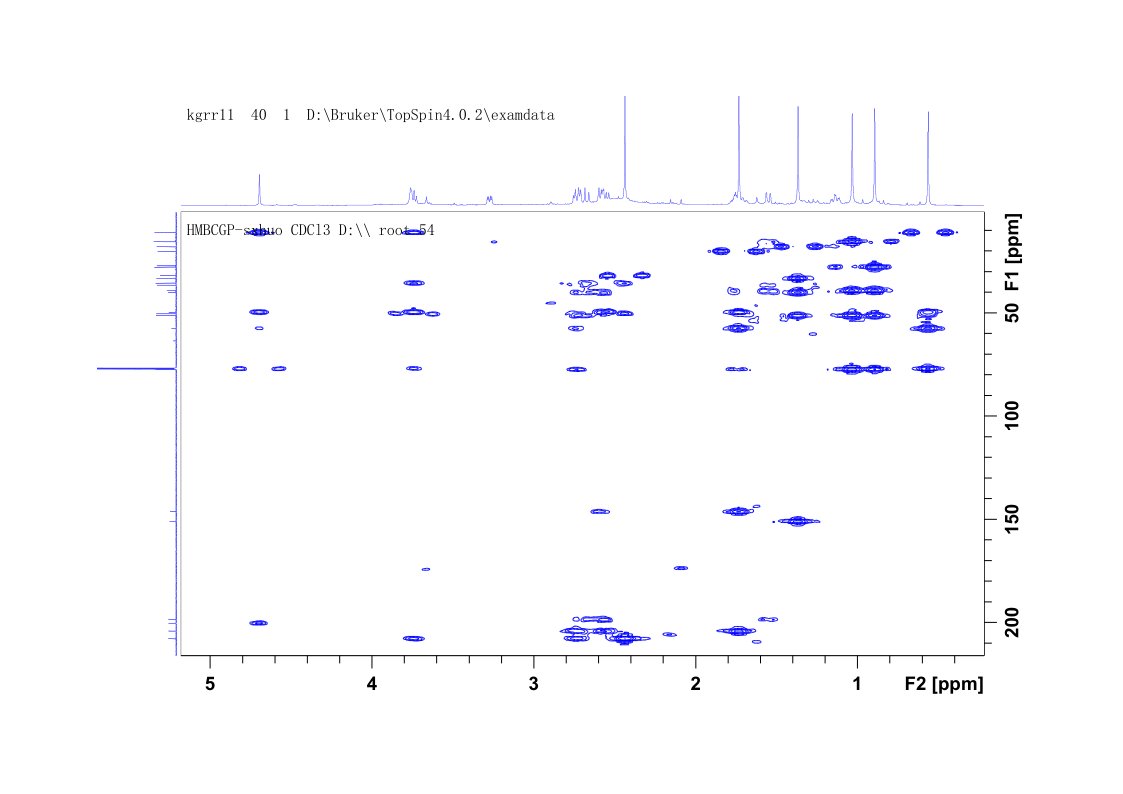


**Figure S52**. HMBC spectrum (600/150 MHz, CDCl_3_) of compound **9**.


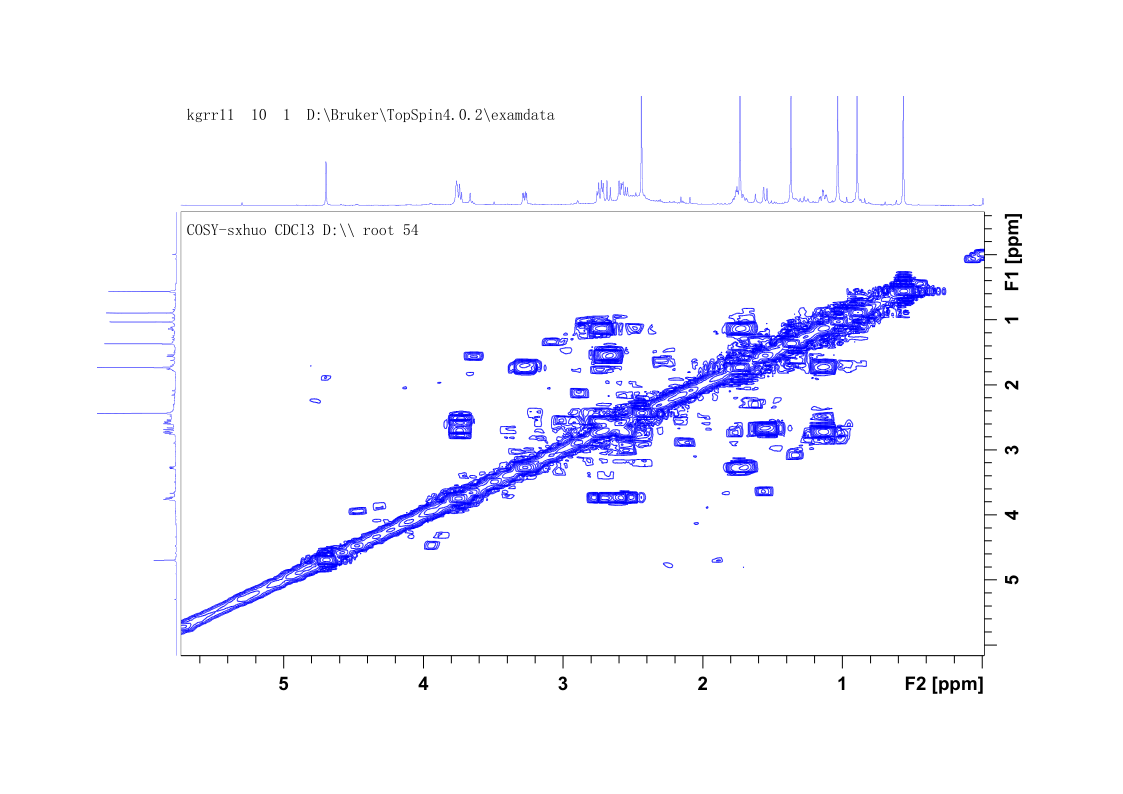


**Figure S53**. ^1^H-^1^H COSY spectrum (600 MHz, CDCl_3_) of compound **9**.


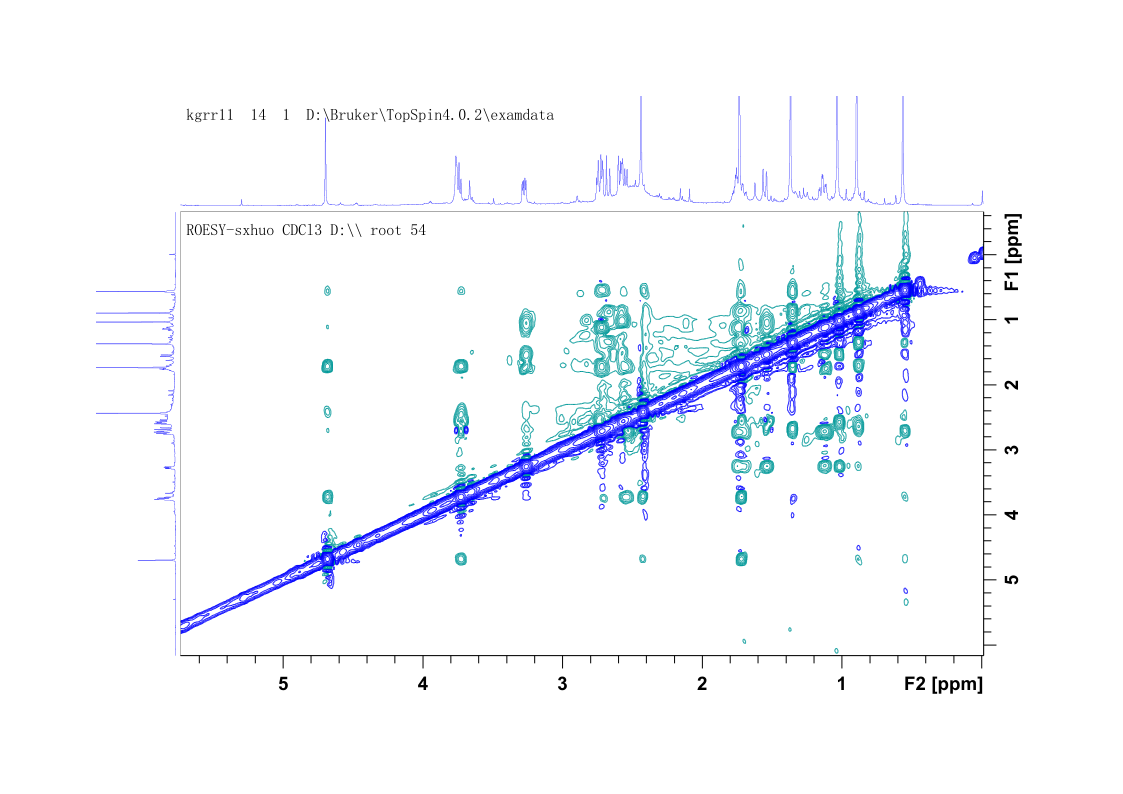


**Figure S54**. ROESY spectrum (600 MHz, CDCl_3_) of compound **9**.


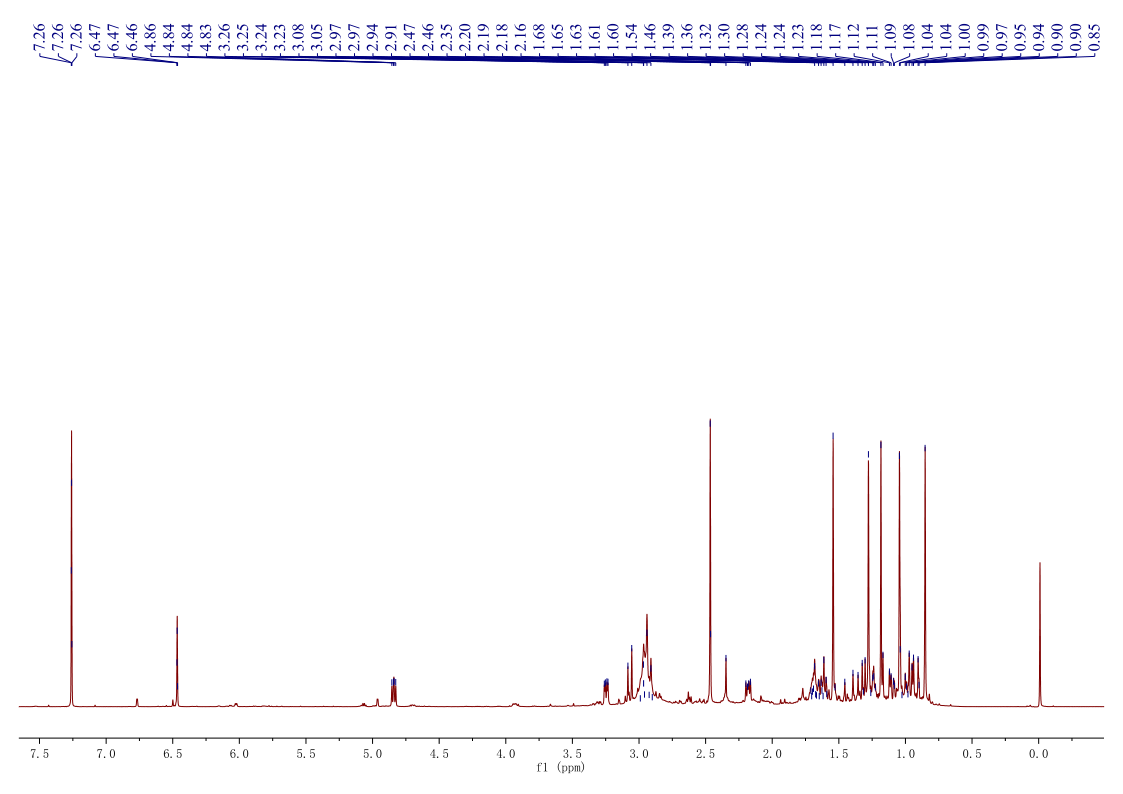


**Figure S55**. ^1^H NMR spectrum (600 MHz, CDCl_3_) of compound **10**.


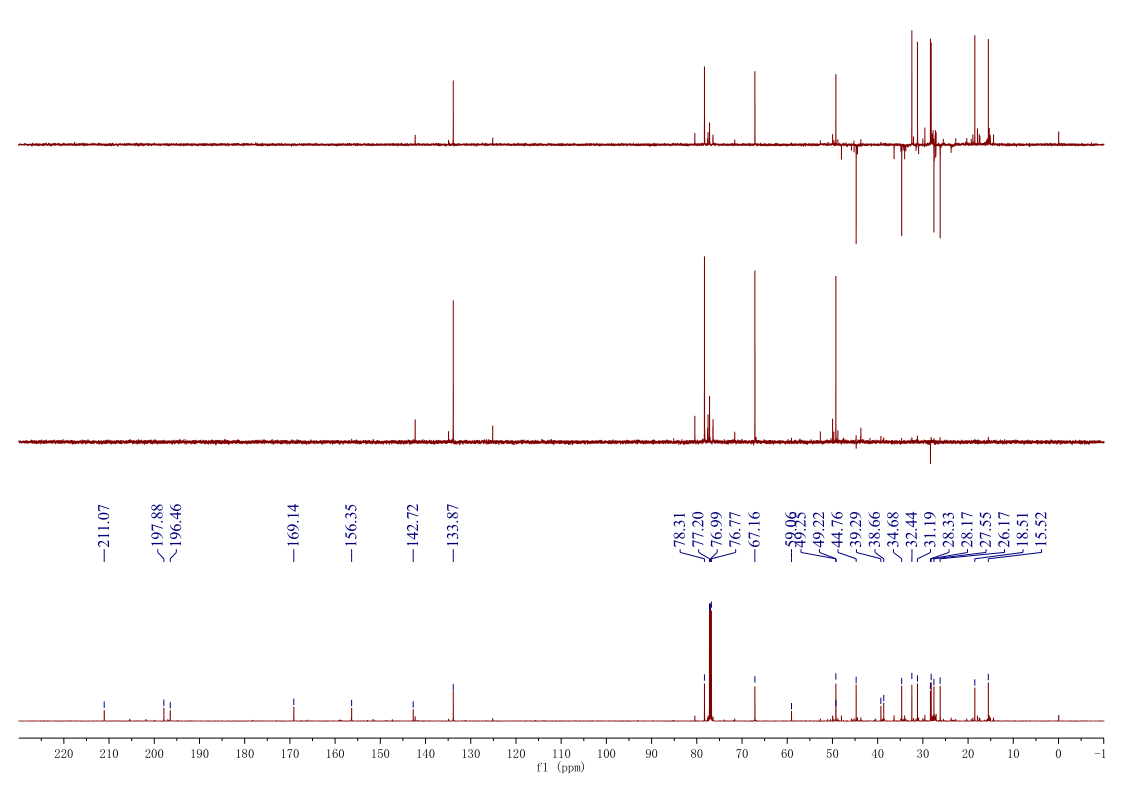


**Figure S56**. ^13^C NMR spectrum (150 MHz, CDCl_3_) of compound **10**.


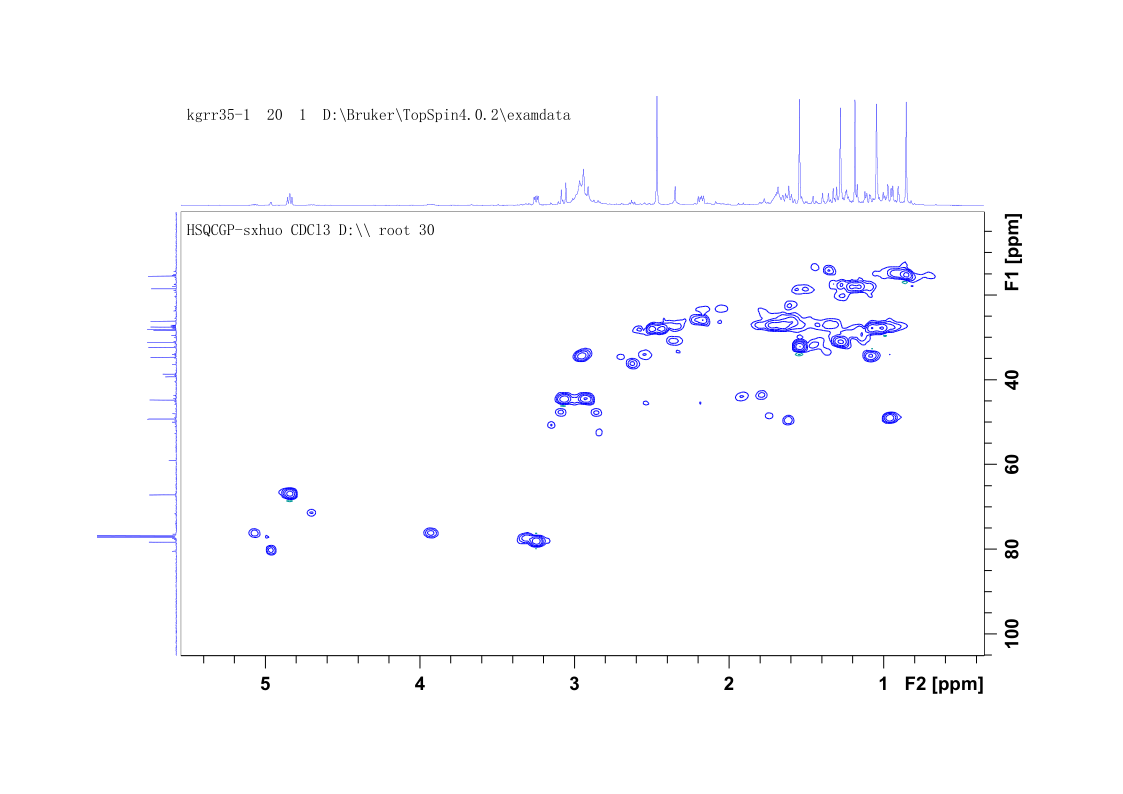


**Figure S57**. HSQC spectrum (600/150 MHz, CDCl_3_) of compound **10**.


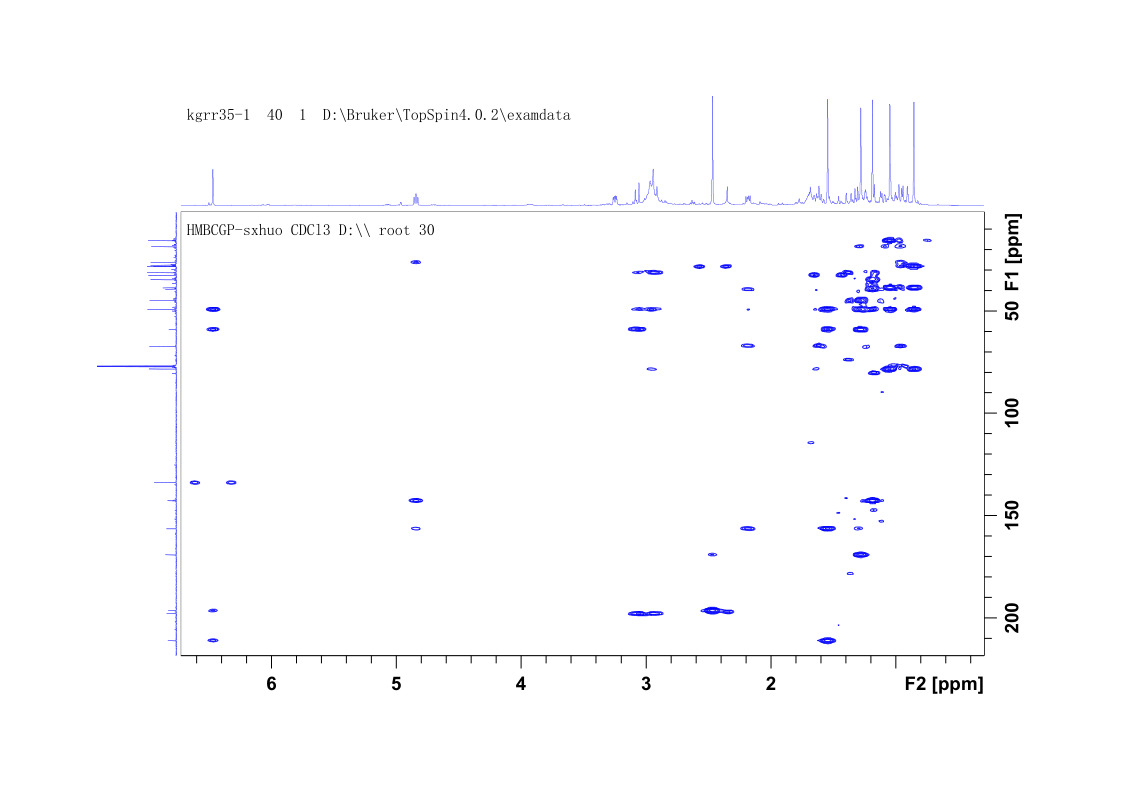


**Figure S58**. HMBC spectrum (600/150 MHz, CDCl_3_) of compound **10**.


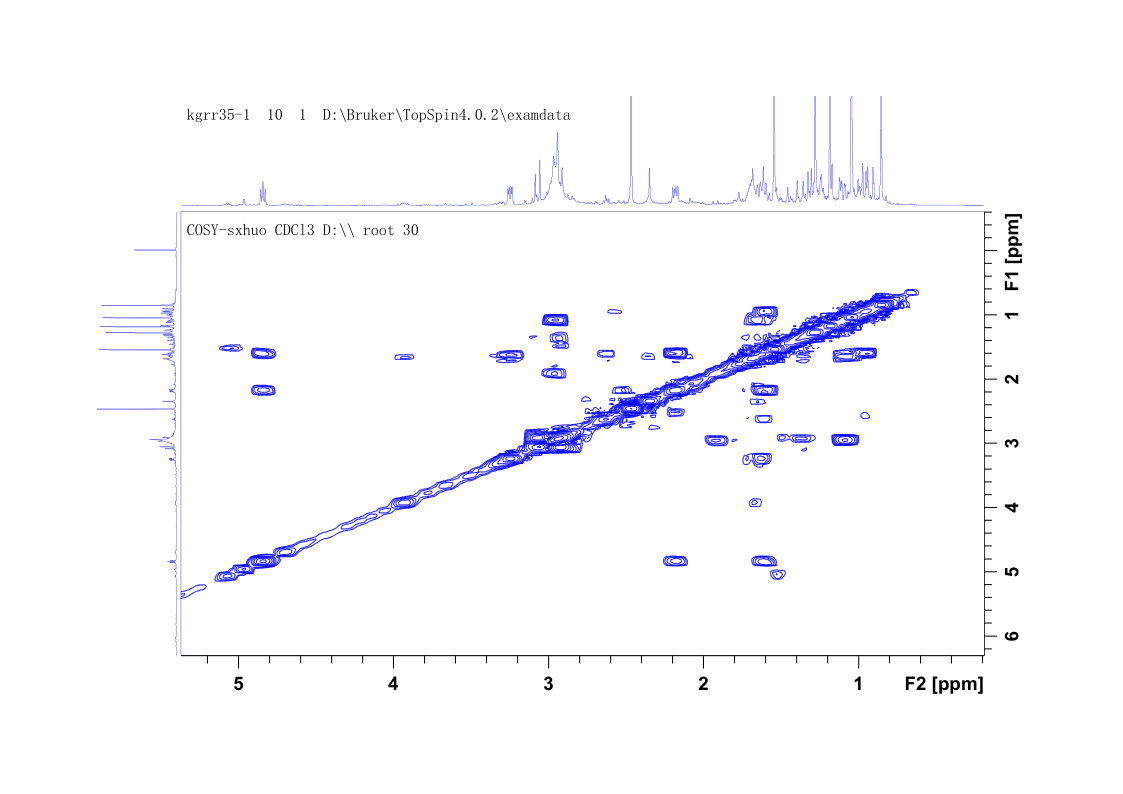


**Figure S59**. ^1^H-^1^H COSY spectrum (600 MHz, CDCl_3_) of compound **10**.


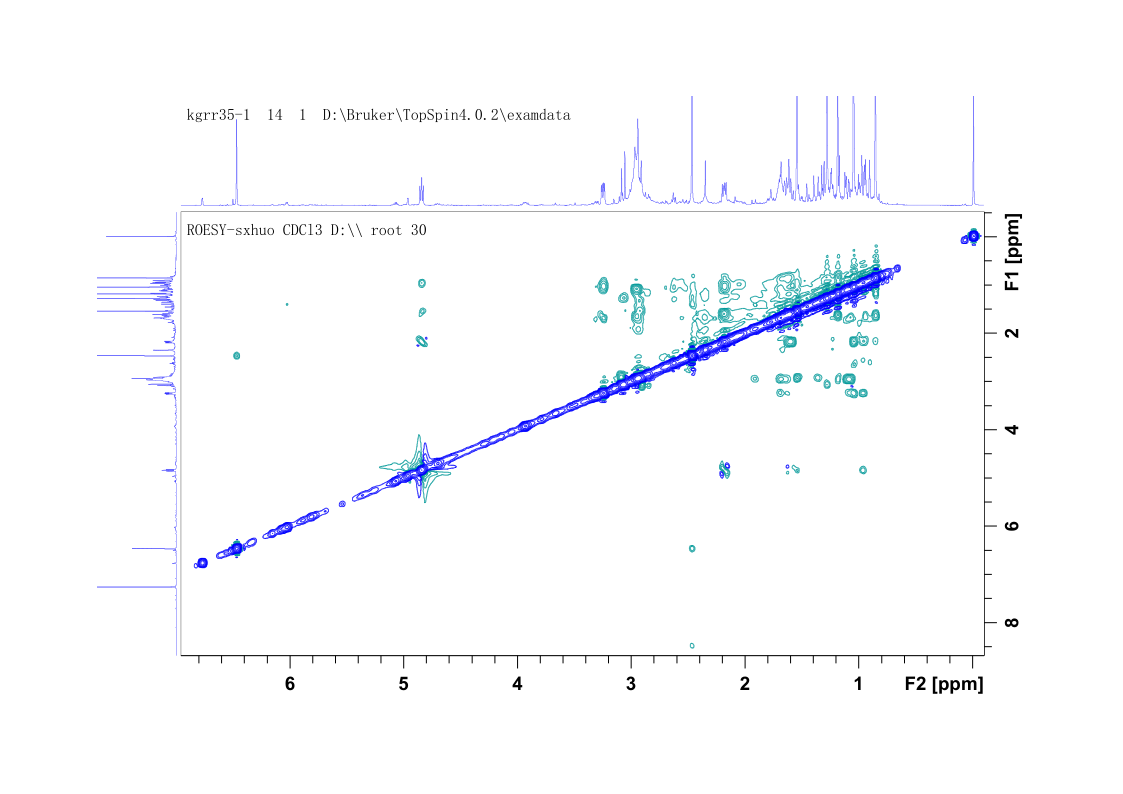


**Figure S60**. ROESY spectrum (600 MHz, CDCl_3_) of compound **10**.


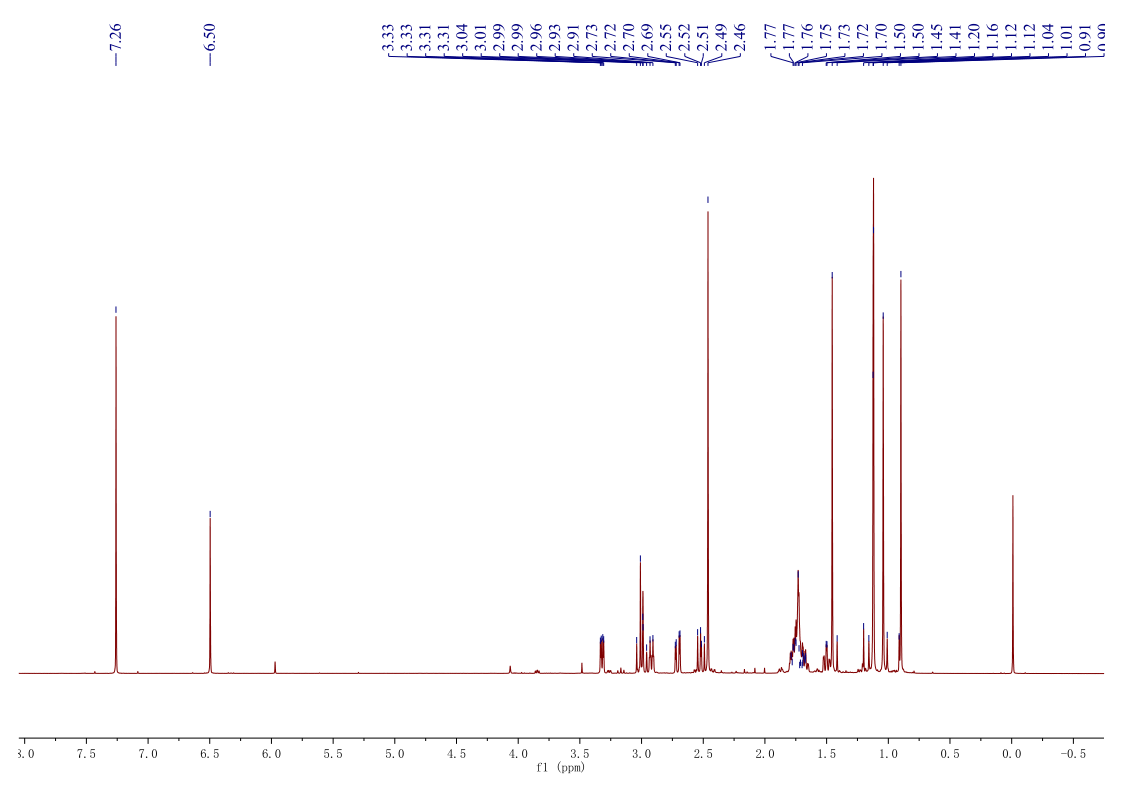


**Figure S61**. ^1^H NMR spectrum (600 MHz, CDCl_3_) of compound **11**.


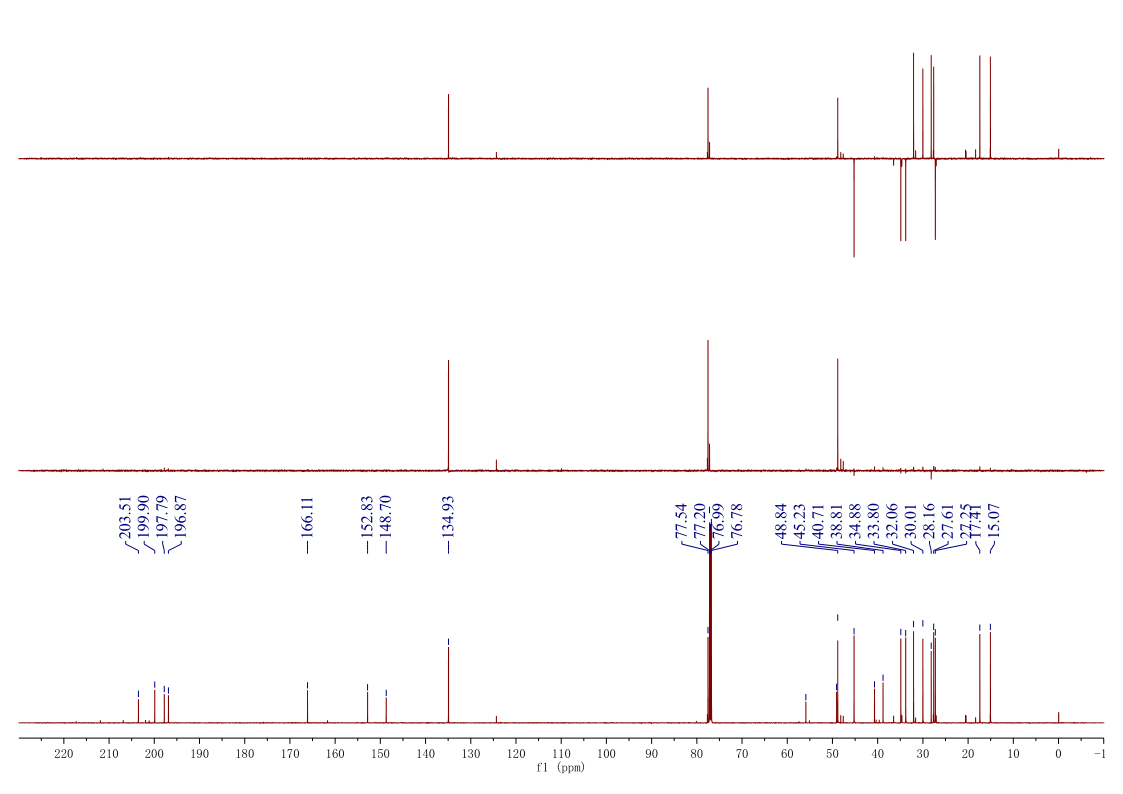


**Figure S62**. ^13^C NMR spectrum (150 MHz, CDCl_3_) of compound **11**.


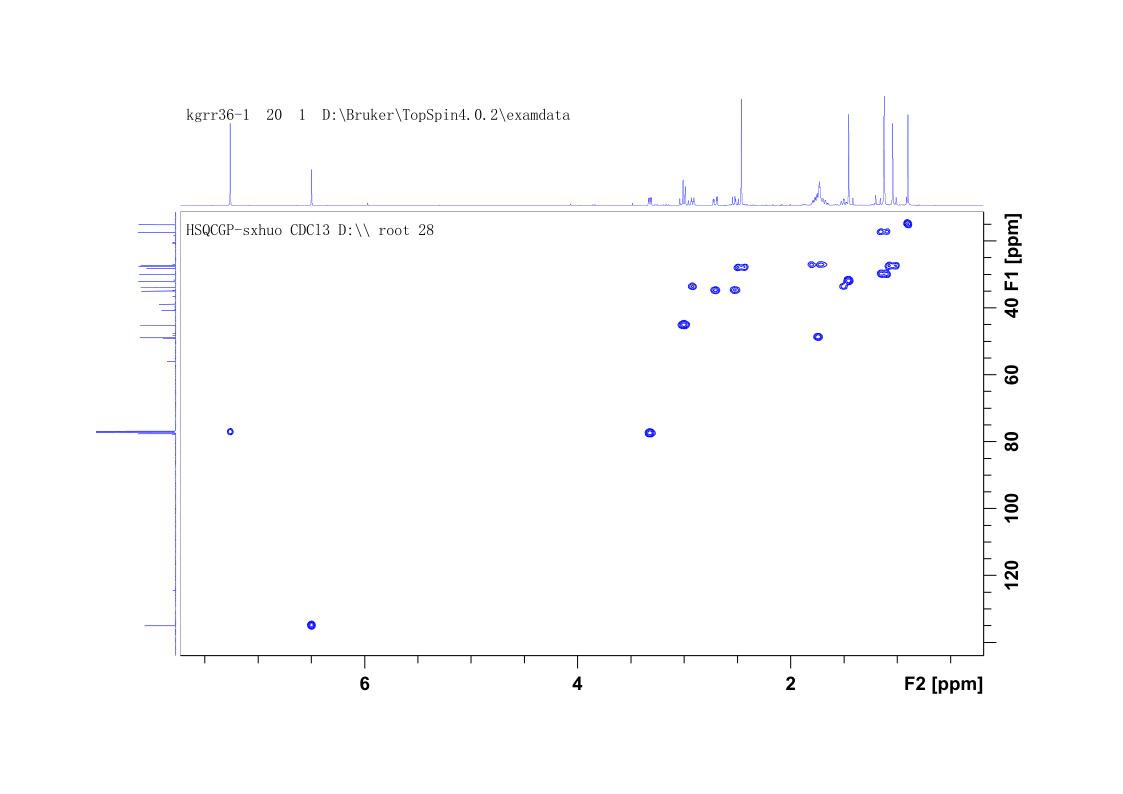


**Figure S63**. HSQC spectrum (600/150 MHz, CDCl_3_) of compound **11**.


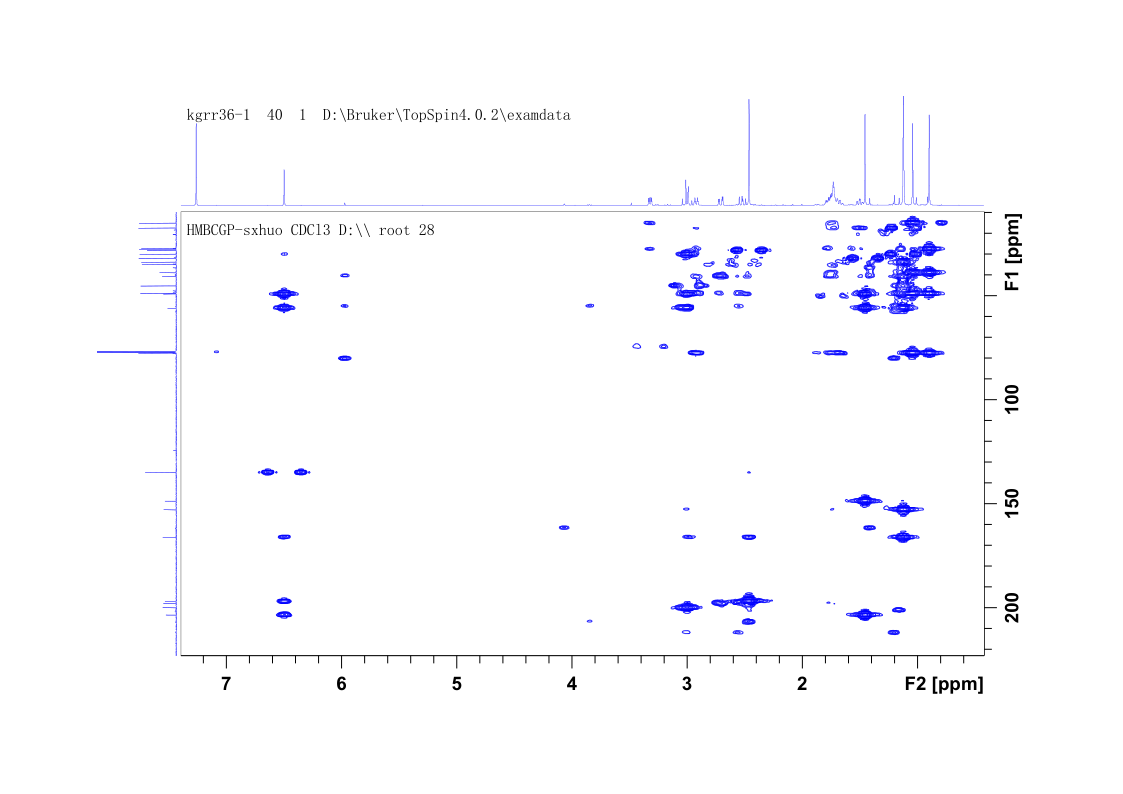


**Figure S64**. HMBC spectrum (600/150 MHz, CDCl_3_) of compound **11**.


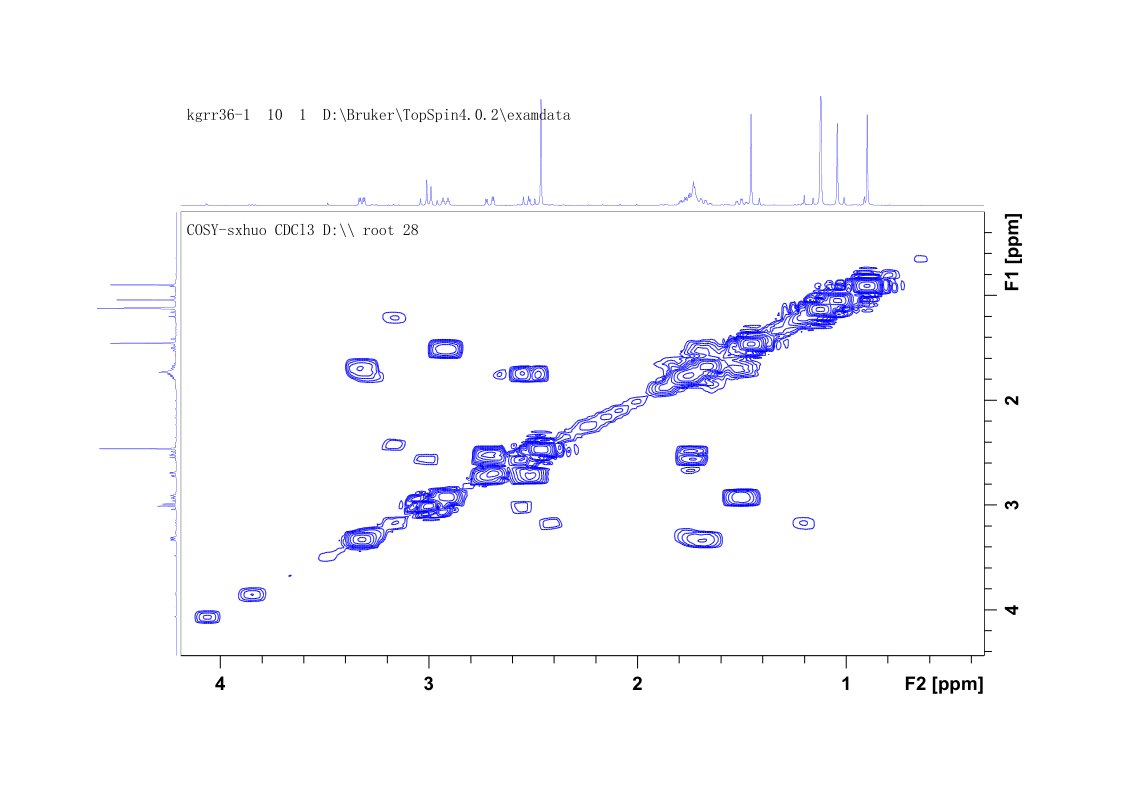


**Figure S65**. ^1^H-^1^H COSY spectrum (600/150 MHz, CDCl_3_) of compound **11**.


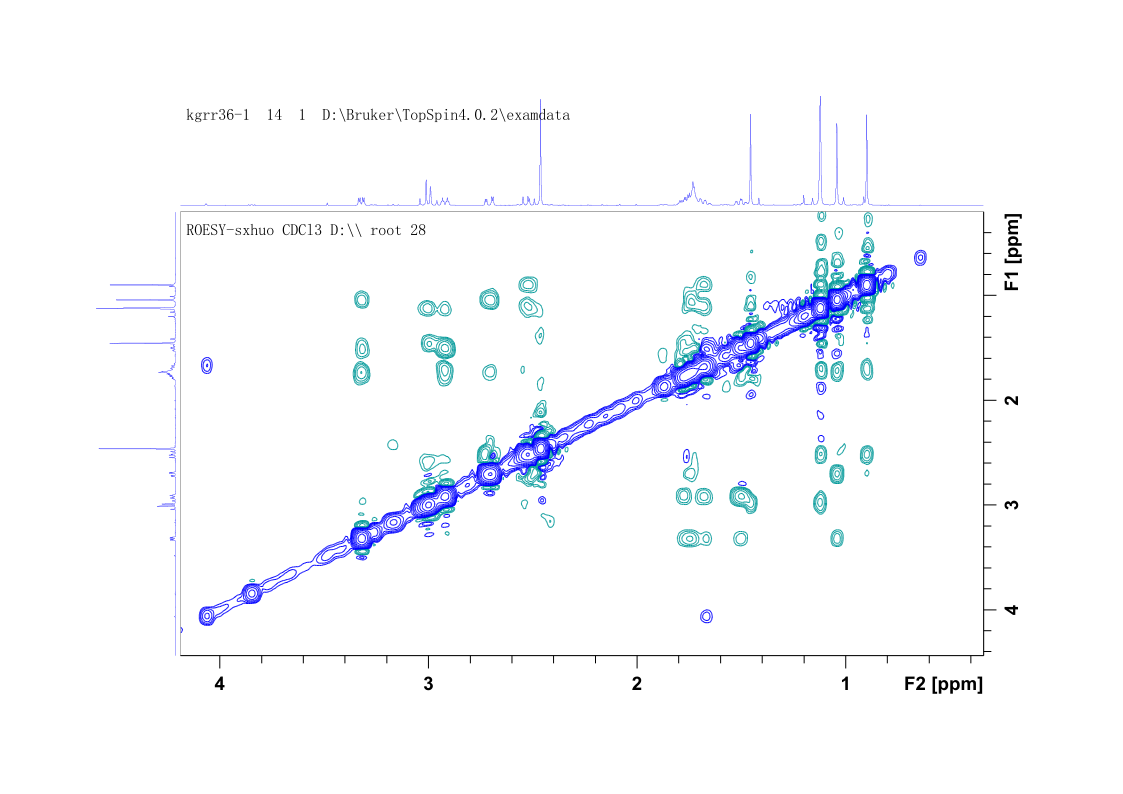


**Figure S66**. ROESY spectrum (600/150 MHz, CDCl_3_) of compound **11**.


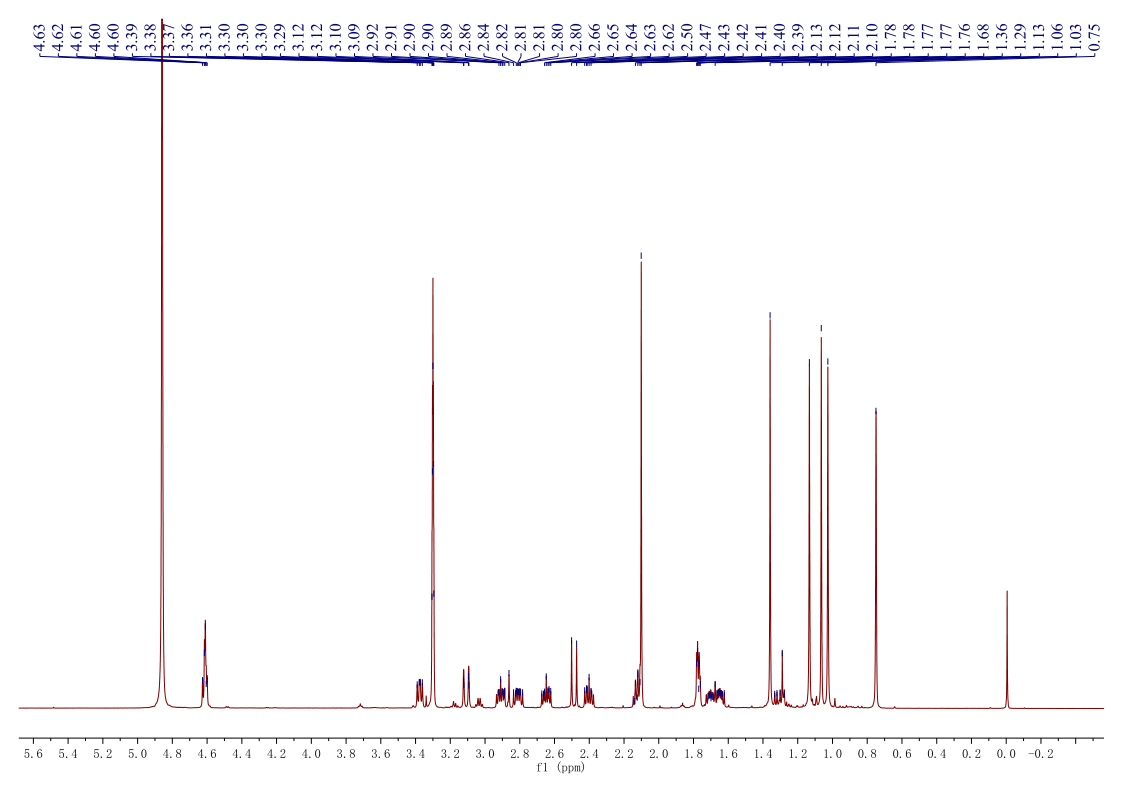


**Figure S67**. ^1^H NMR spectrum (600 MHz, CD_3_OD) of compound **15**.


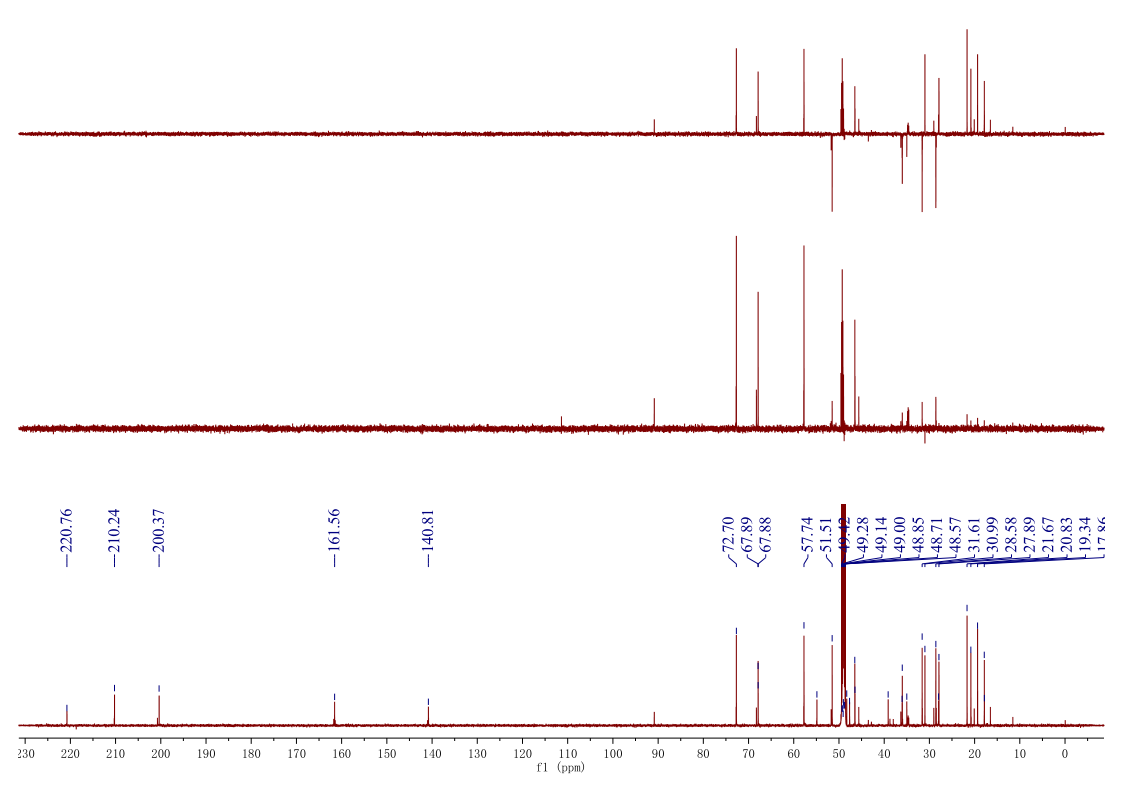


**Figure S68**. ^13^C NMR spectrum (150 MHz, CD_3_OD) of compound **15**.


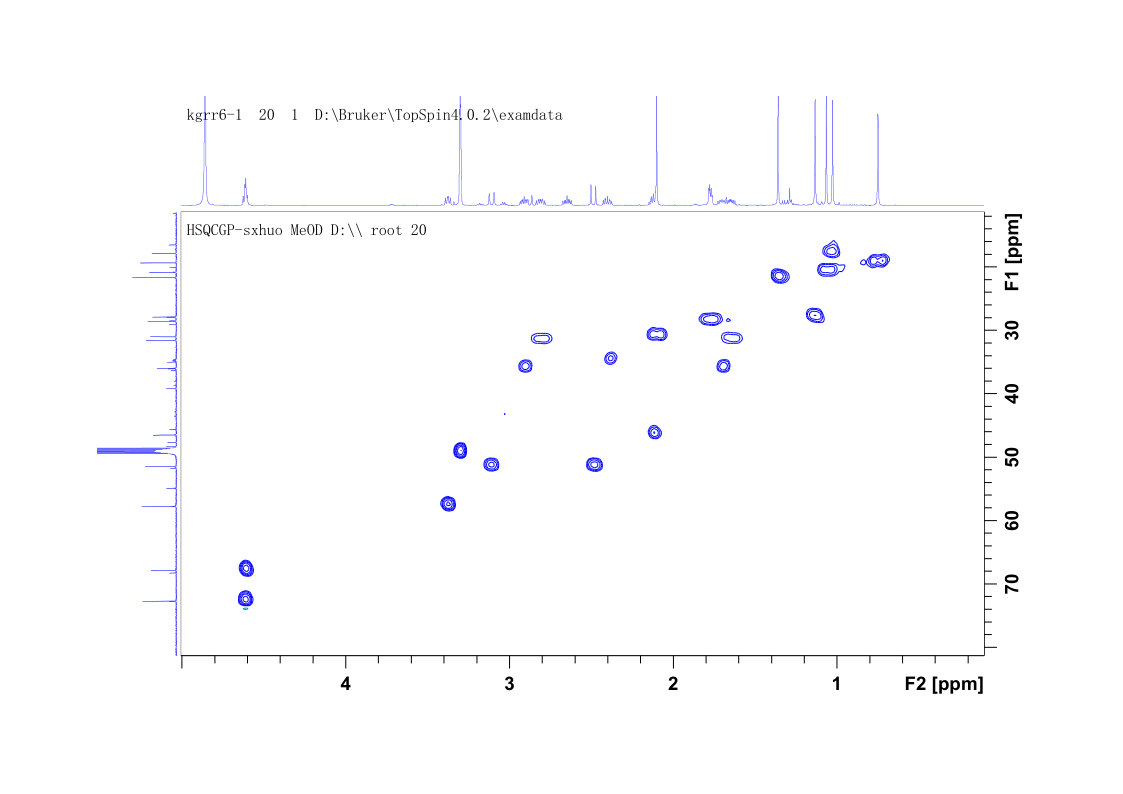


**Figure S69**. HSQC spectrum (600/150 MHz, CD_3_OD) of compound **15**.


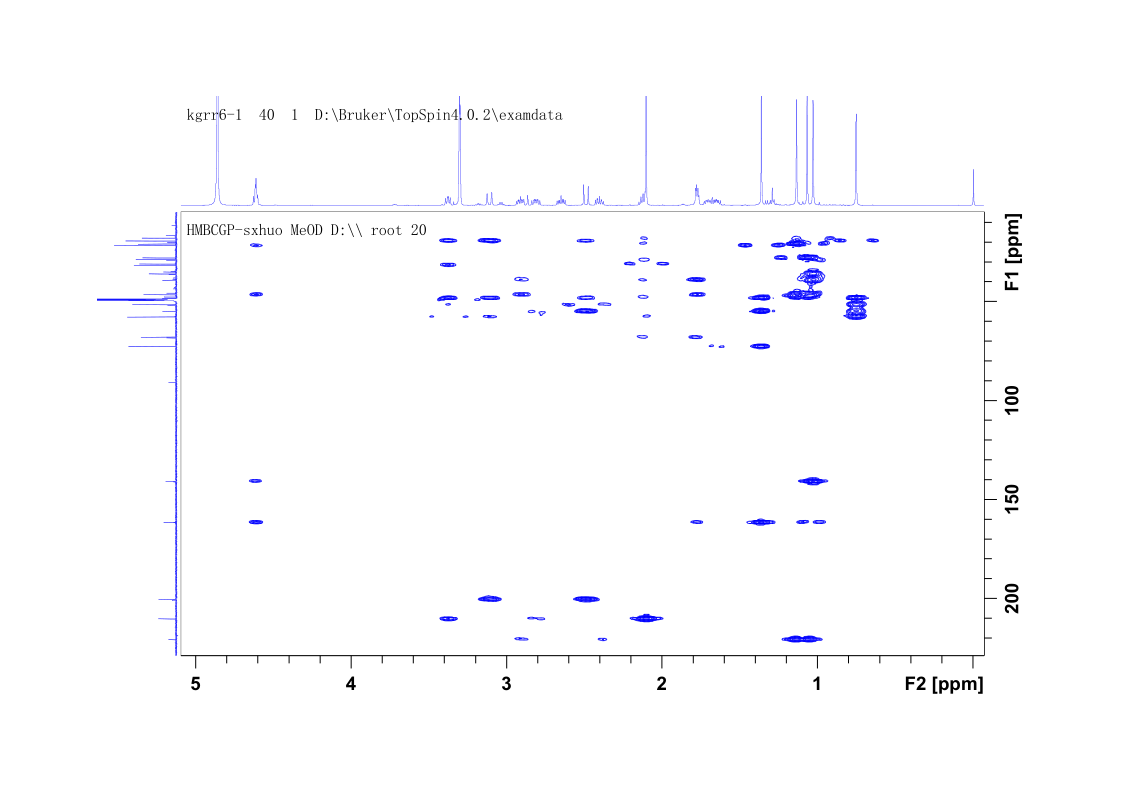


**Figure S70**. HMBC spectrum (600/150 MHz, CD_3_OD) of compound **15**.


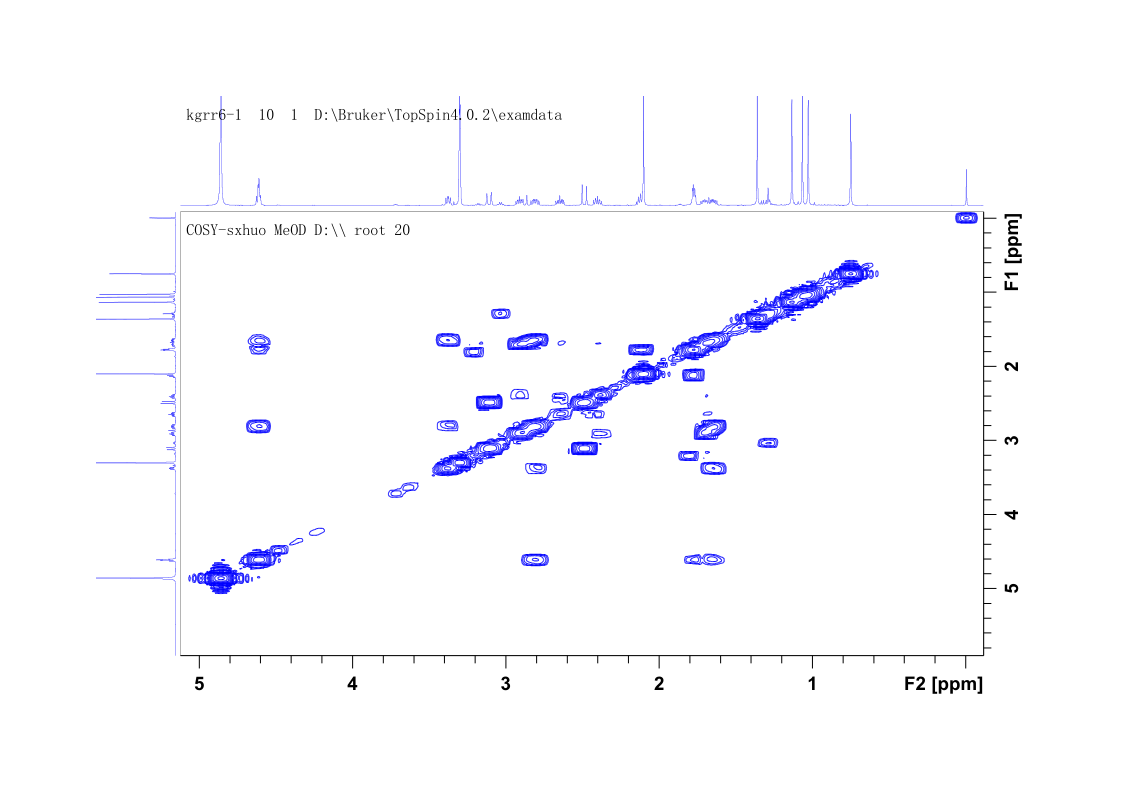


**Figure S71**. ^1^H-^1^H COSY spectrum (600 MHz, CD_3_OD) of compound **15**.


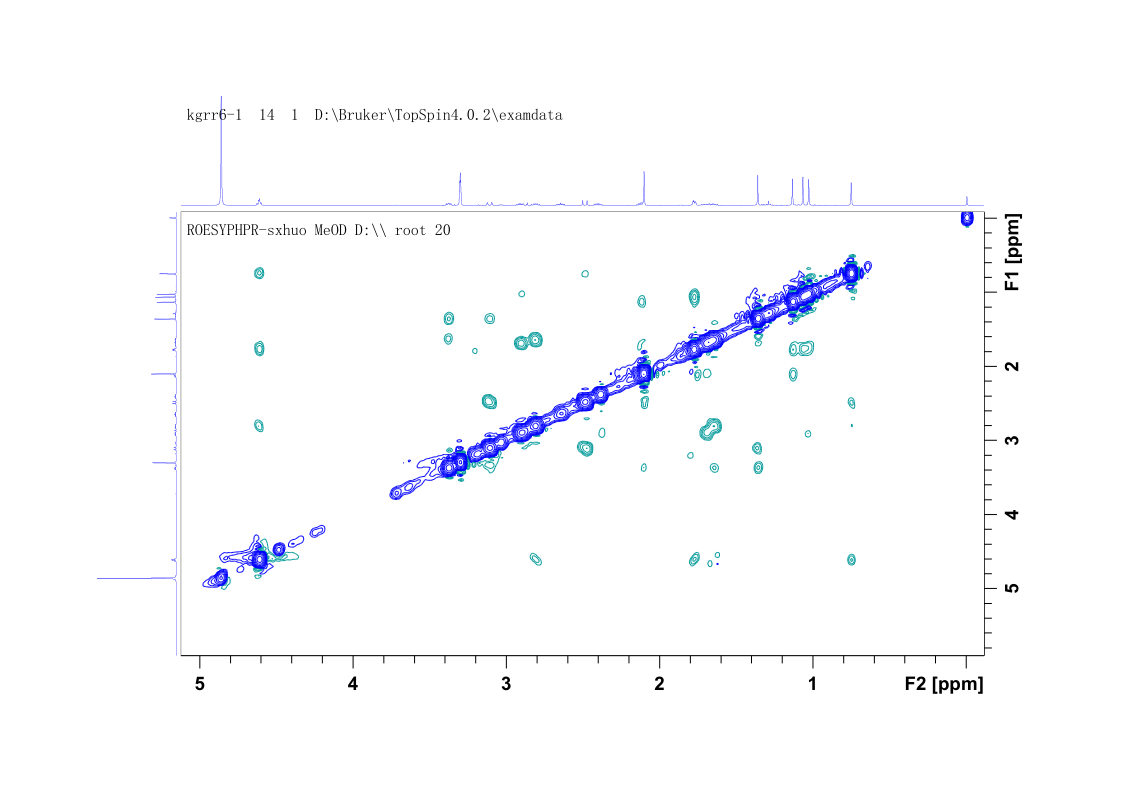


**Figure S72**. ROESY spectrum (600 MHz, CD_3_OD) of compound **15**.


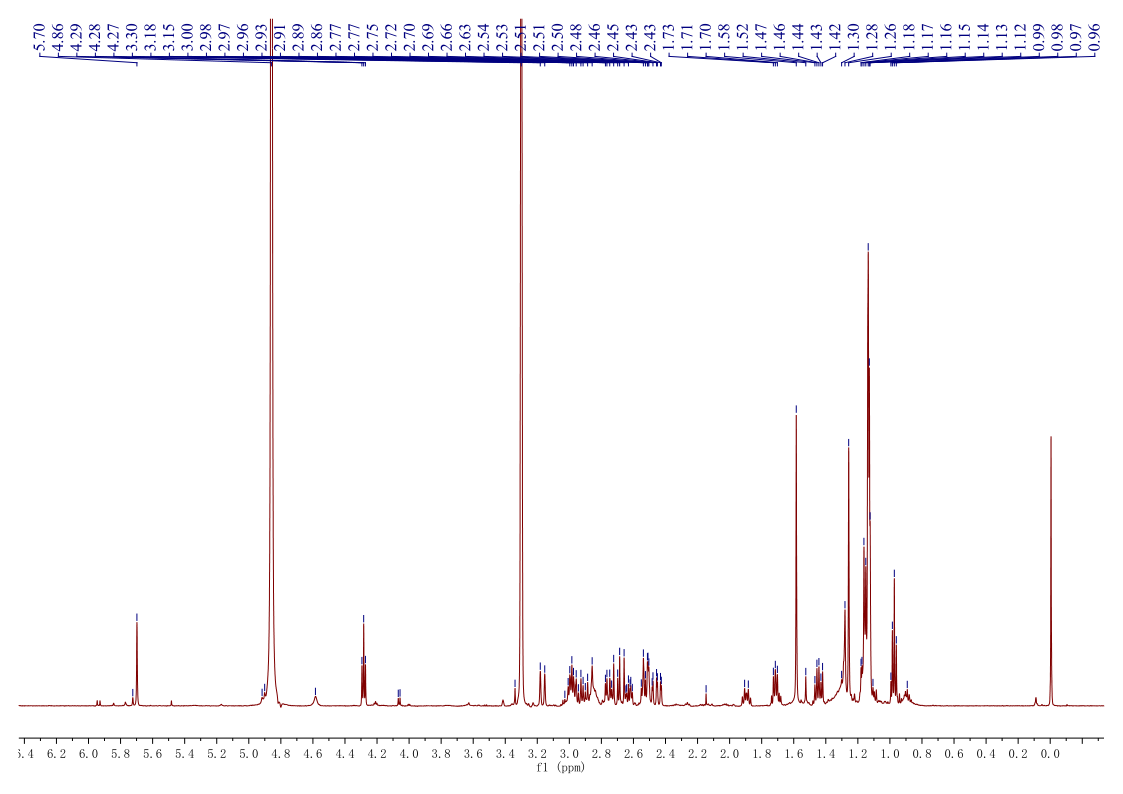


**Figure S73**. ^1^H NMR spectrum (600 MHz, CD_3_OD) of compound **31**.


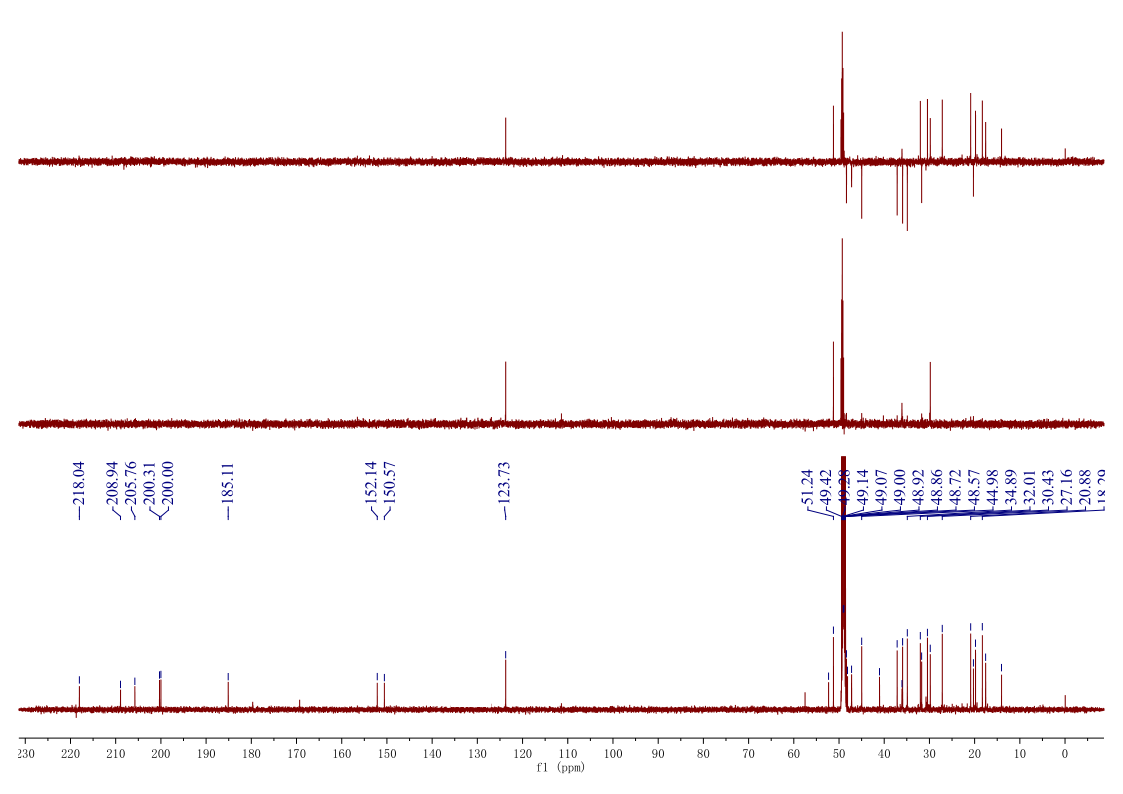


**Figure S74**. ^13^C NMR spectrum (150 MHz, CD_3_OD) of compound **31**.


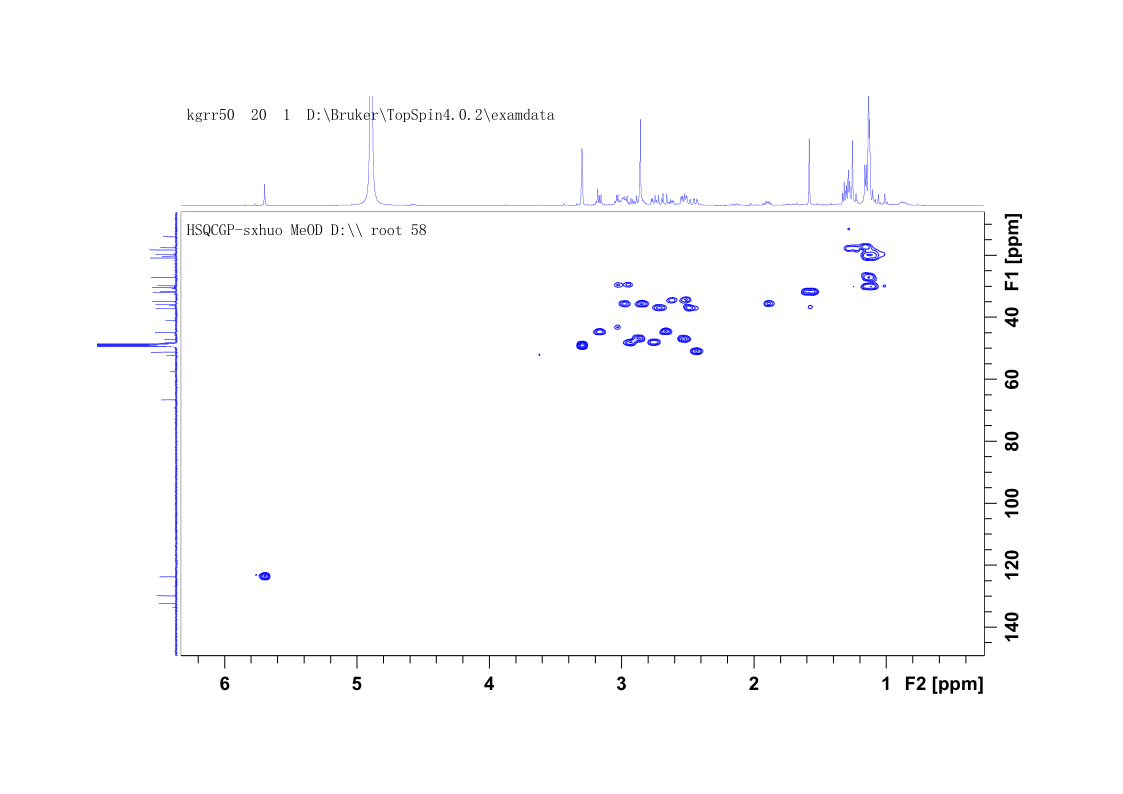


**Figure S75**. HSQC spectrum (600/150 MHz, CD_3_OD) of compound **31**.


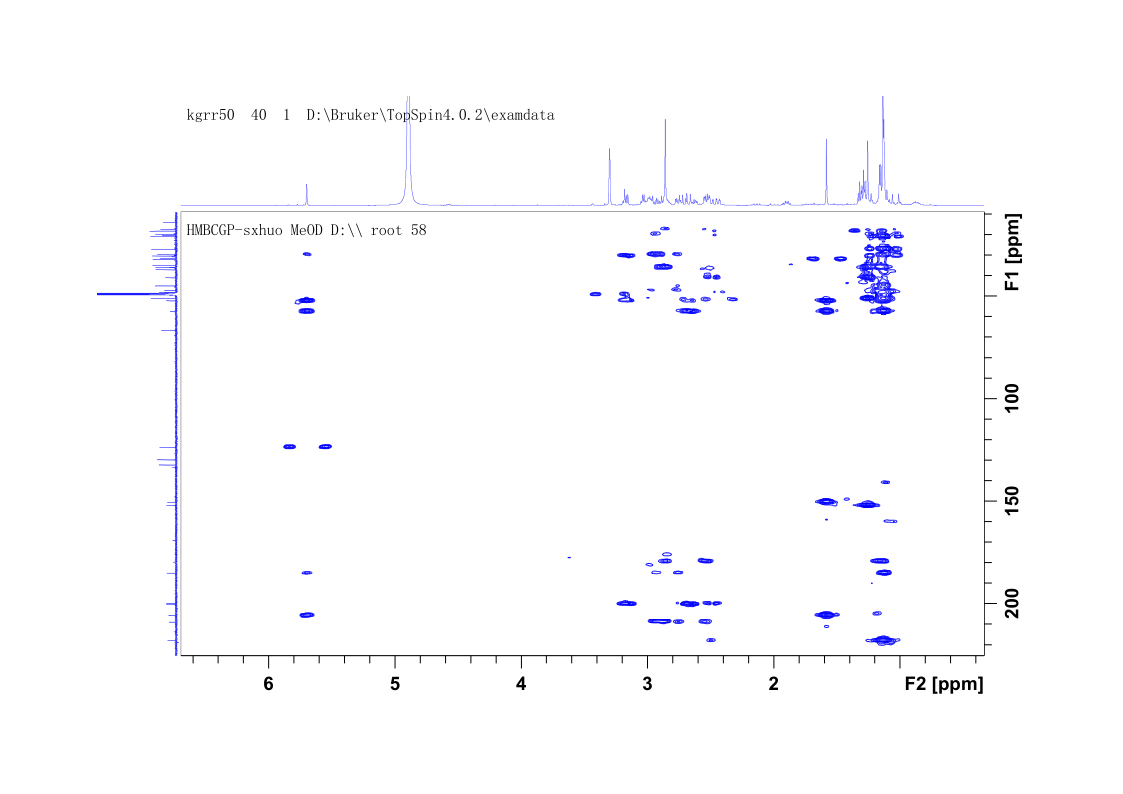


**Figure S76**. HMBC spectrum (600/150 MHz, CD_3_OD) of compound **31**.


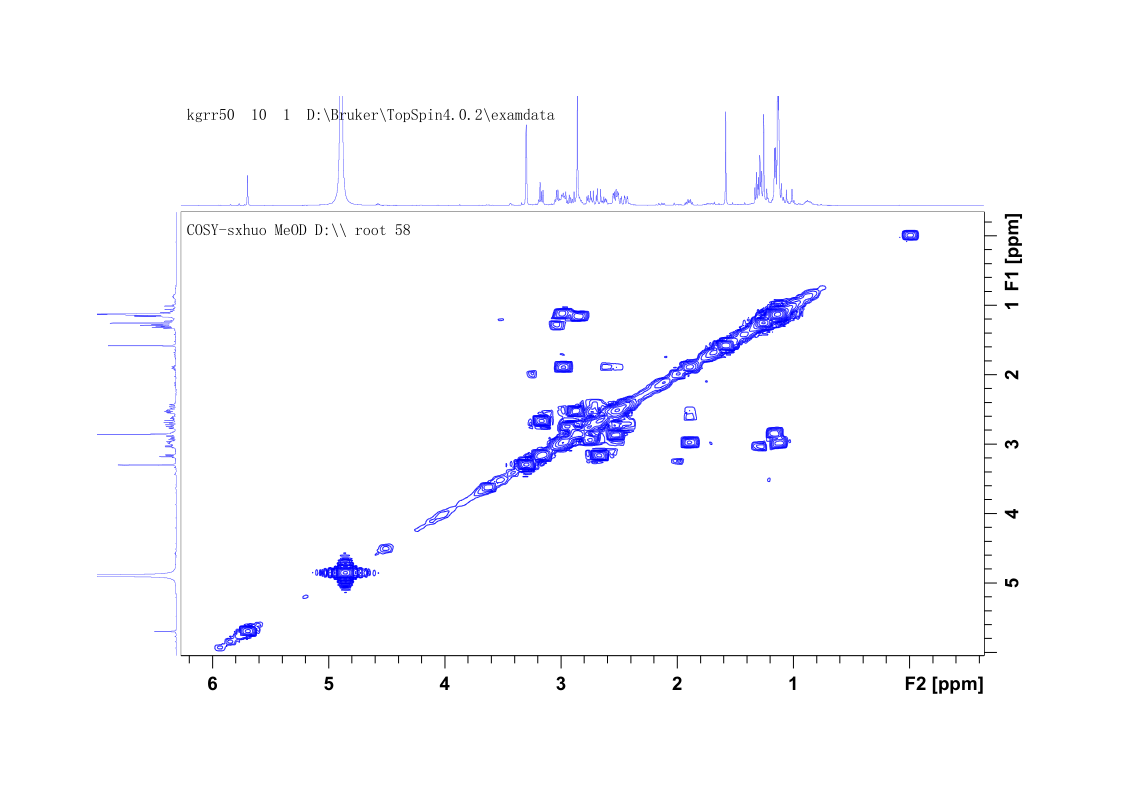


**Figure S77**. ^1^H-^1^H COSY spectrum (600 MHz, CD_3_OD) of compound **31**.


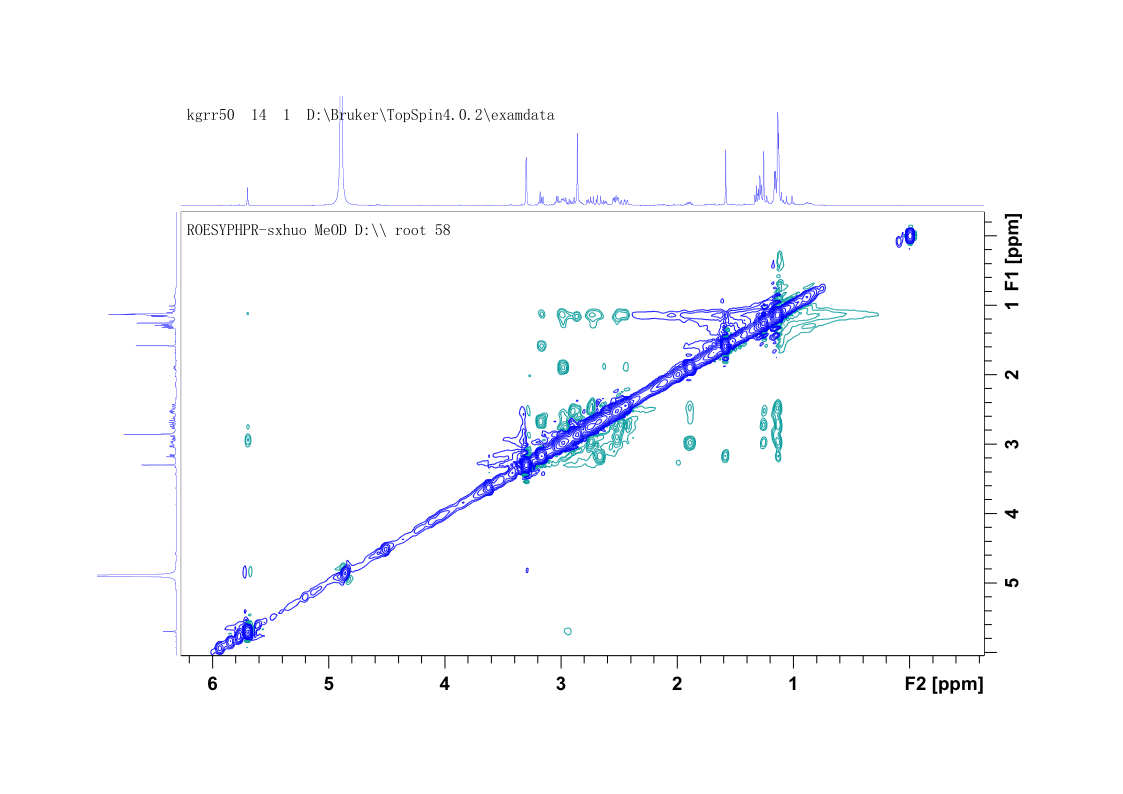


**Figure S78**. ROESY spectrum (600 MHz, CD_3_OD) of compound **31**.


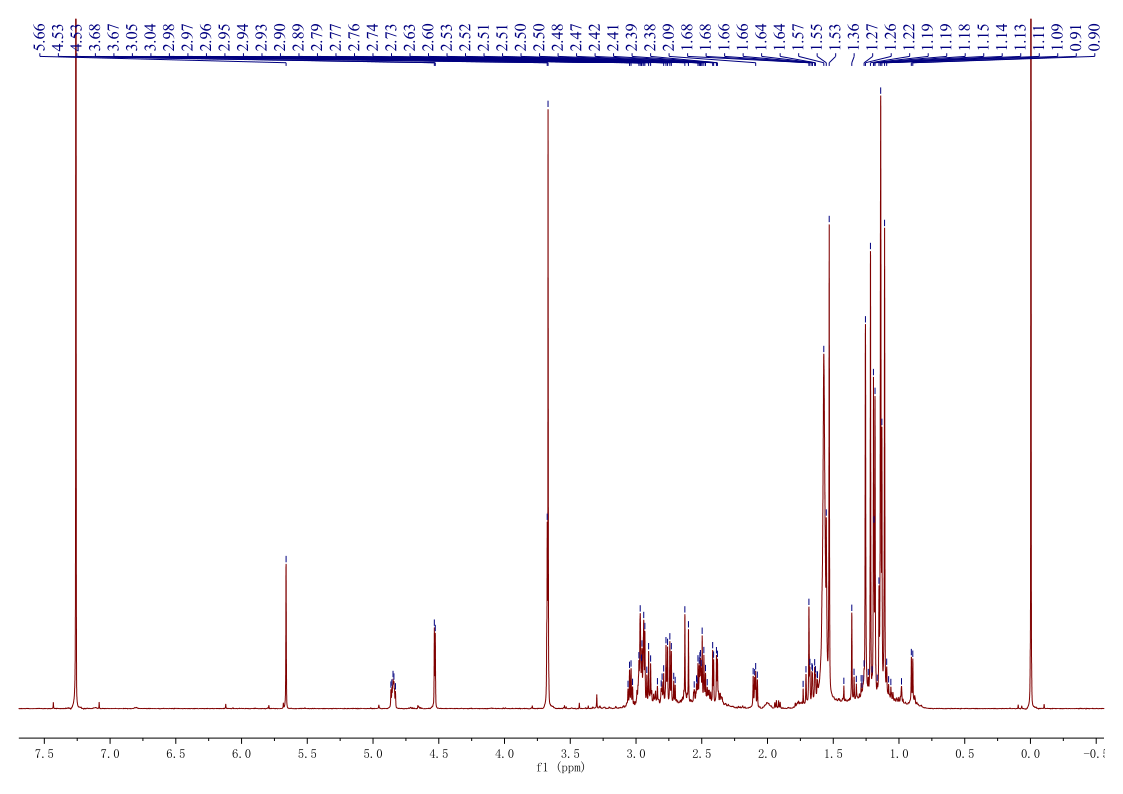


**Figure S79**. ^1^H NMR spectrum (600 MHz, CDCl_3_) of compound **35**.


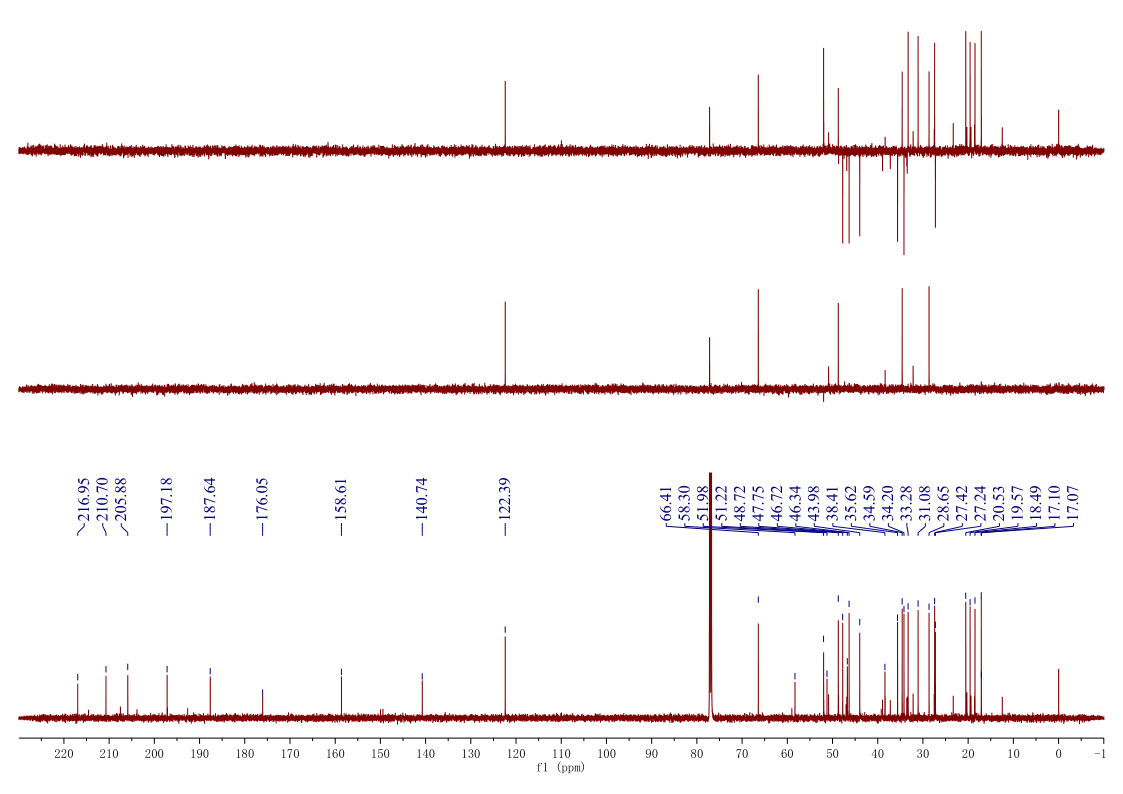


**Figure S80**. ^13^C NMR spectrum (150 MHz, CDCl_3_) of compound **35**.


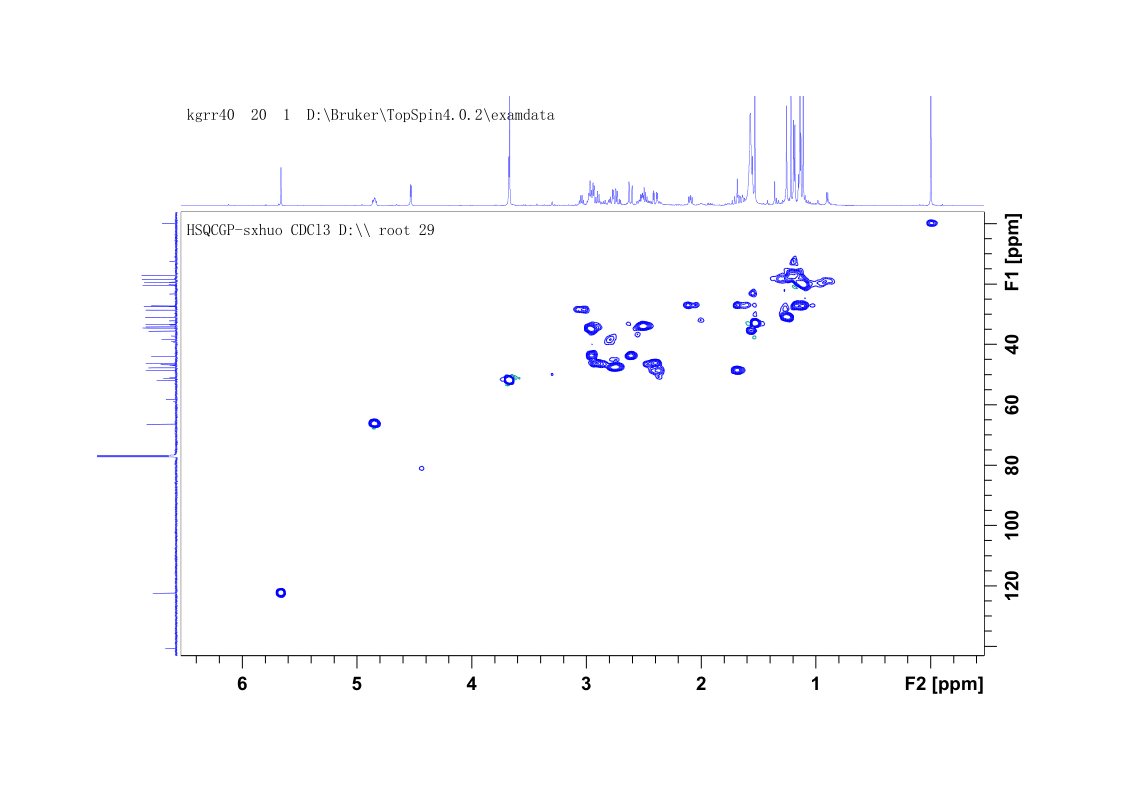


**Figure S81**. HSQC spectrum (600/150 MHz, CDCl_3_) of compound **35**.


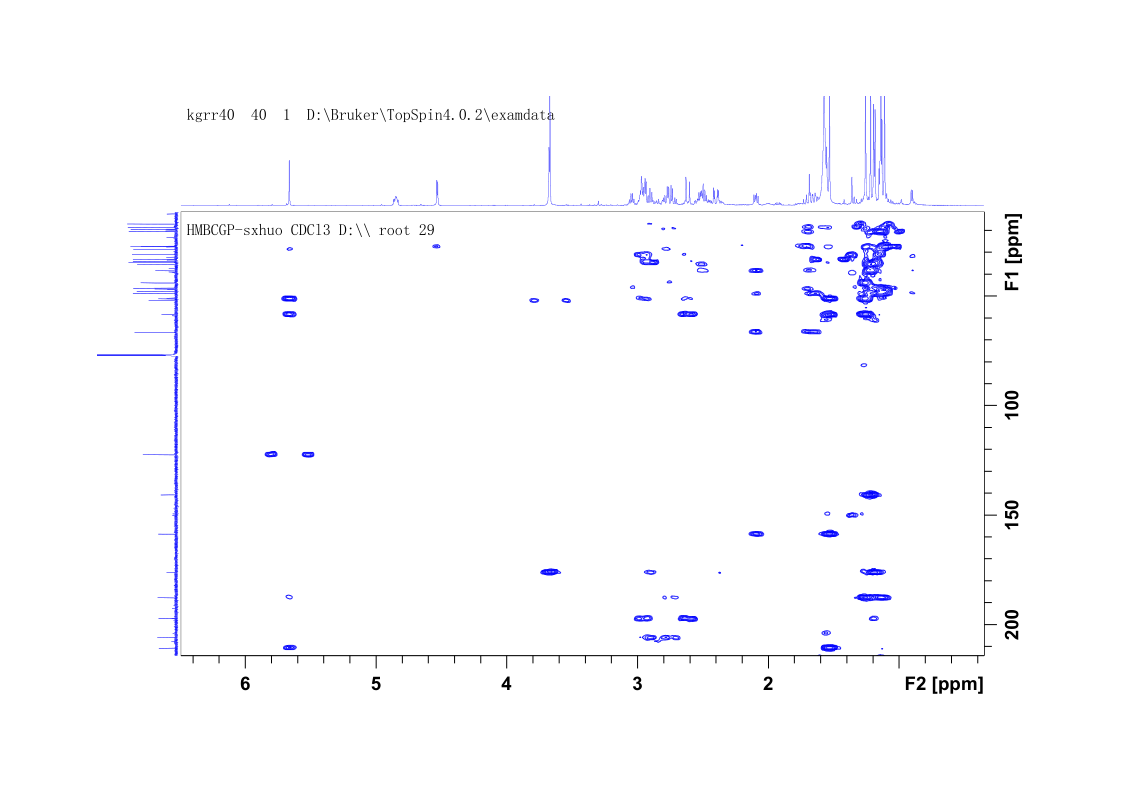


**Figure S82**. HMBC spectrum (600/150 MHz, CDCl_3_) of compound **35**.


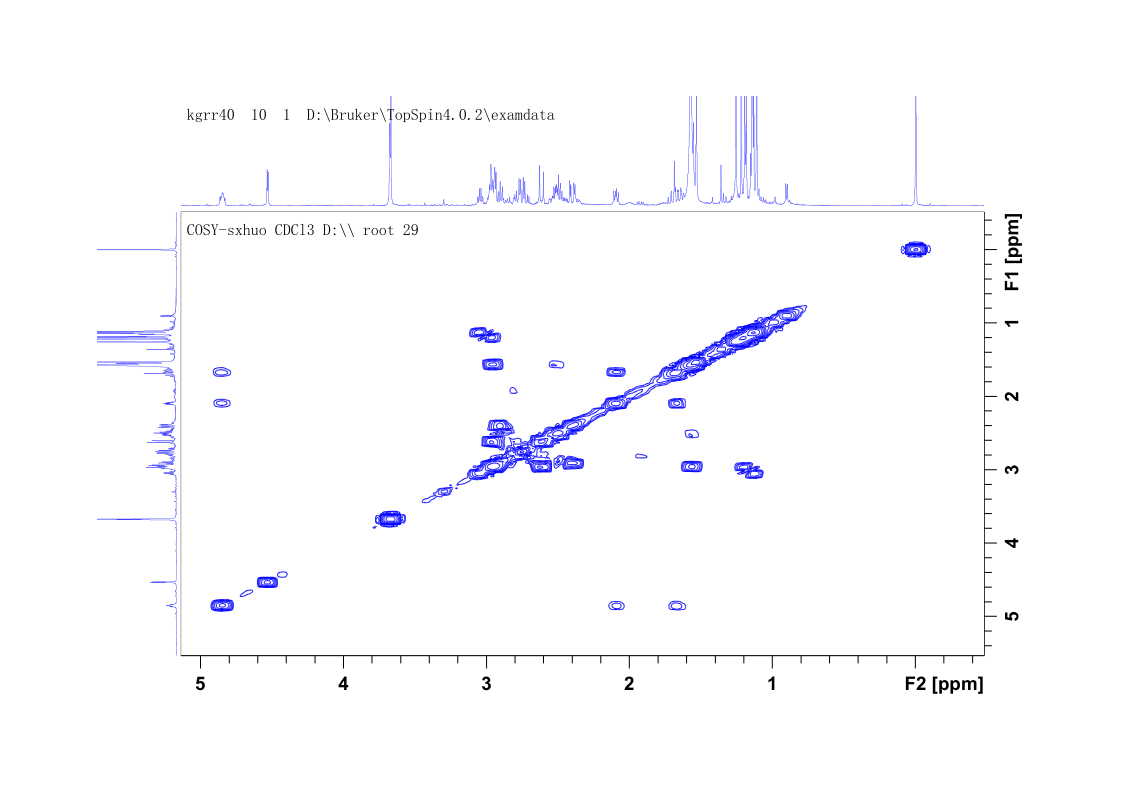


**Figure S83**. ^1^H-^1^H COSY spectrum (600 MHz, CDCl_3_) of compound **35**.


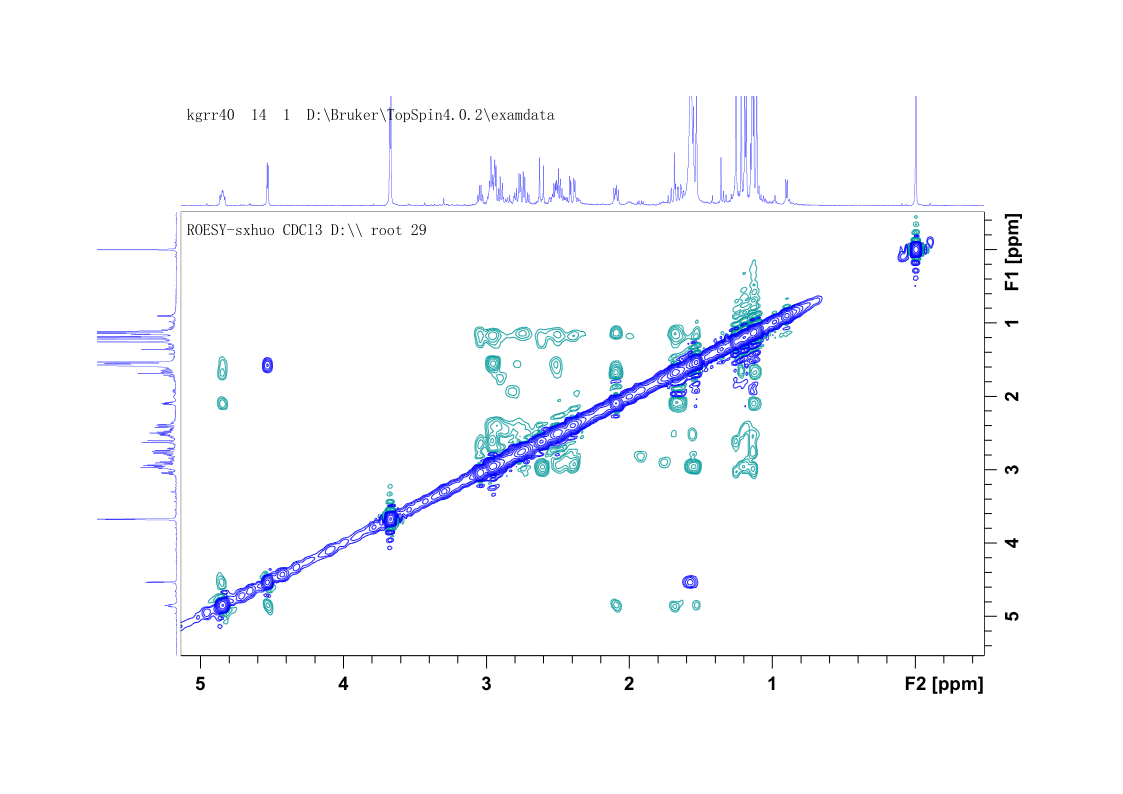


**Figure S84**. ROESY spectrum (600 MHz, CDCl_3_) of compound **35**.


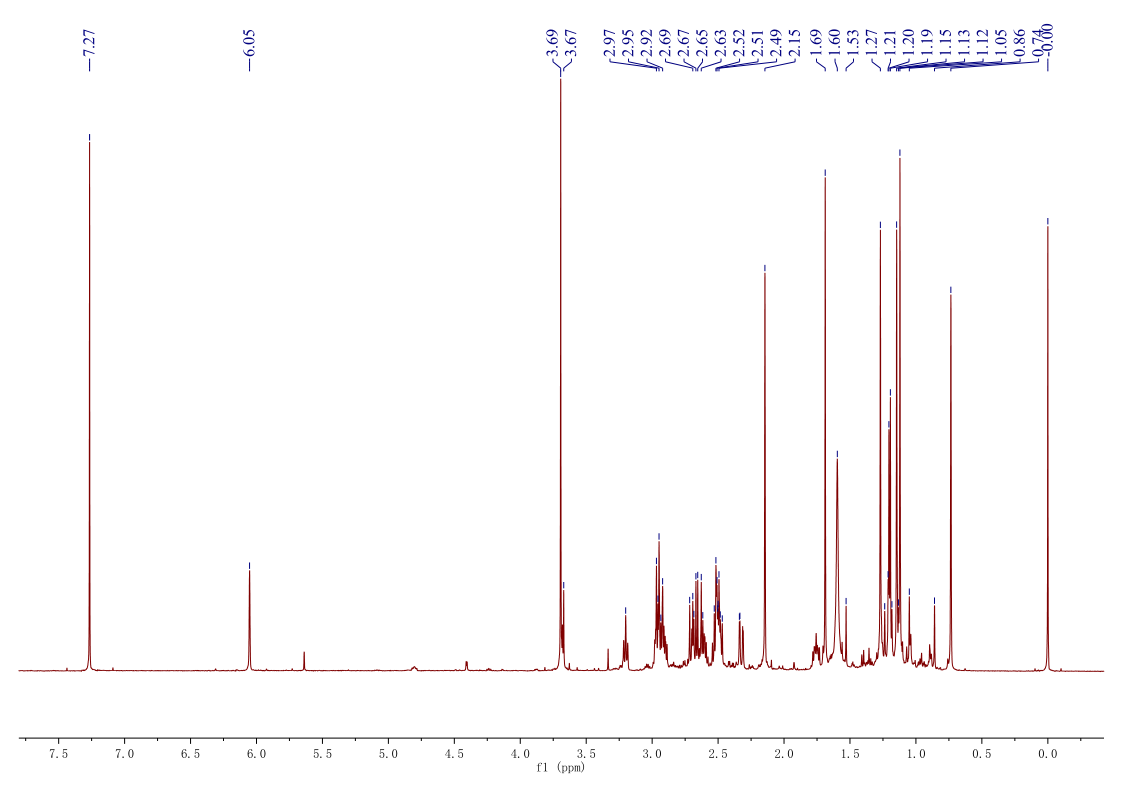


**Figure S85**. ^1^H NMR spectrum (600 MHz, CDCl_3_) of compound **37**.


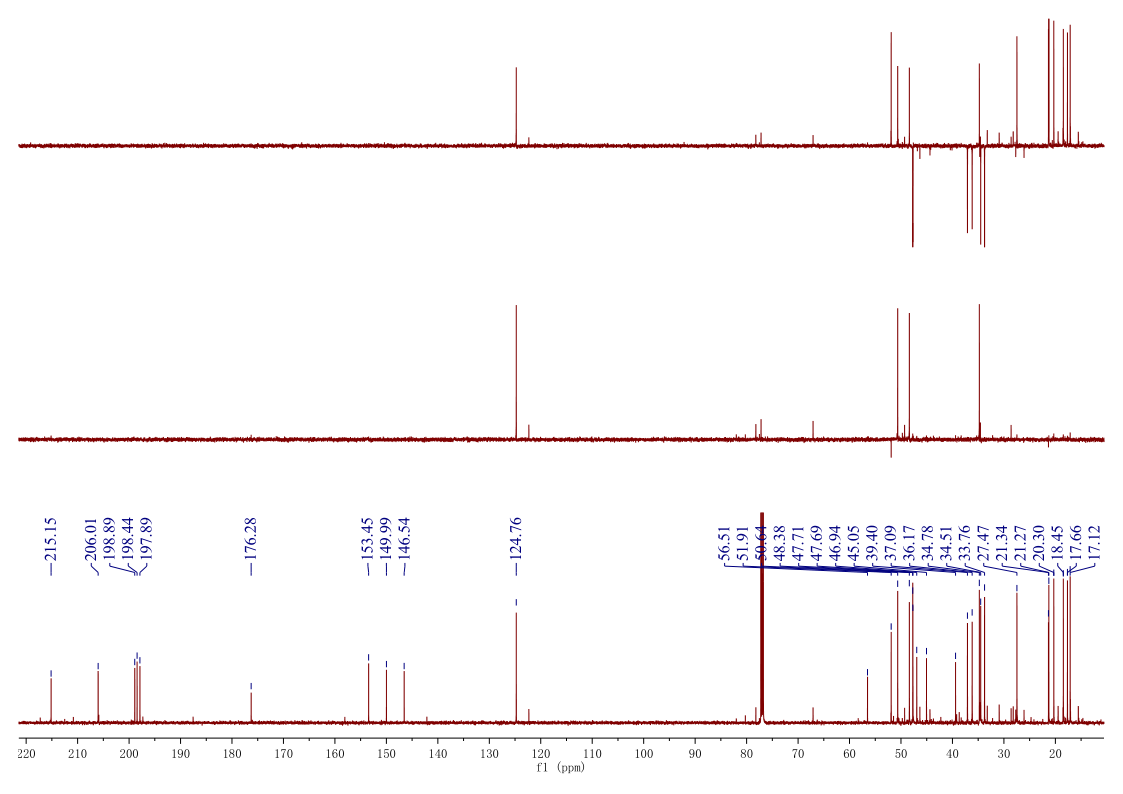


**Figure S86**. ^13^C NMR spectrum (150 MHz, CDCl_3_) of compound **37**.


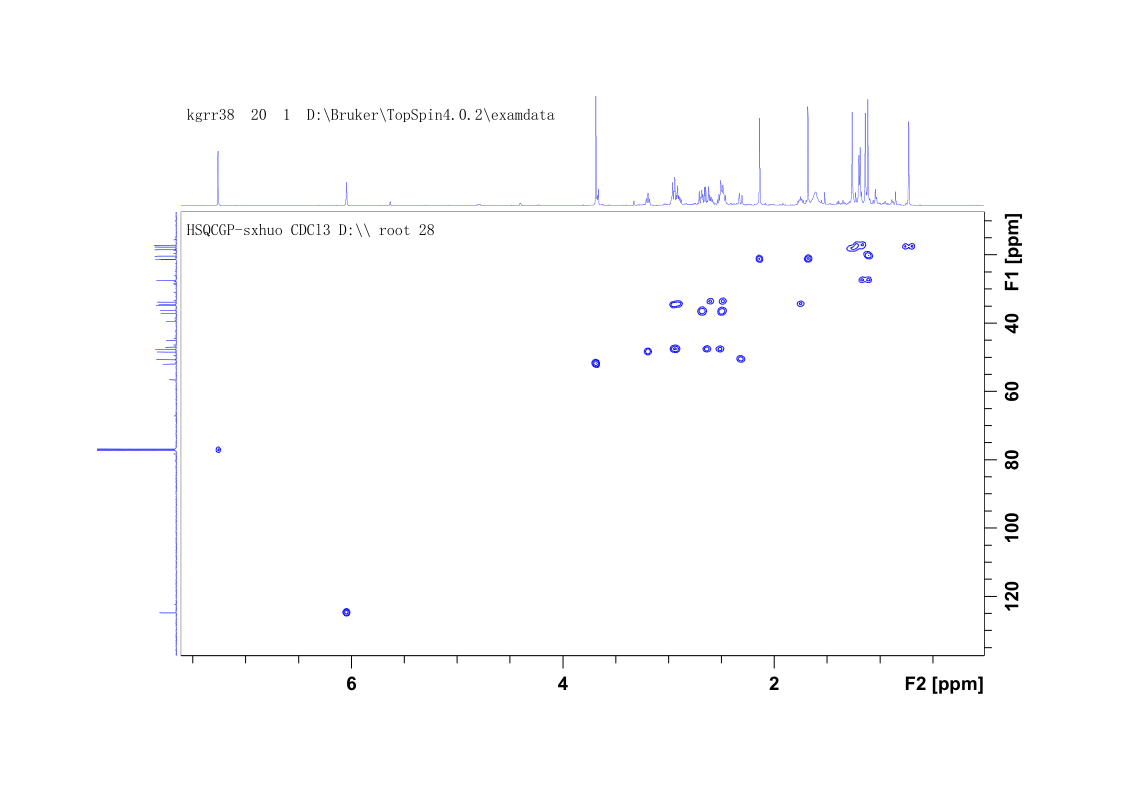


**Figure S87**. HSQC spectrum (600/150 MHz, CDCl_3_) of compound **37**.


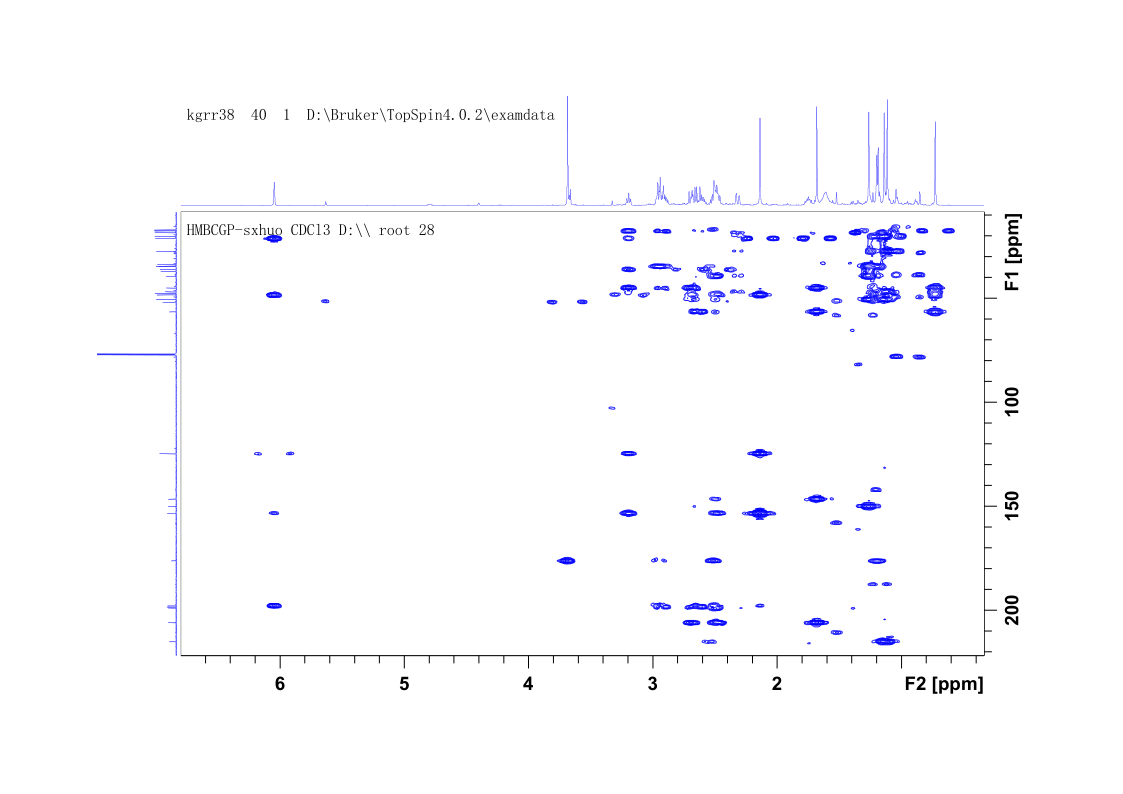


**Figure S88**. HMBC spectrum (600/150 MHz, CDCl_3_) of compound **37**.


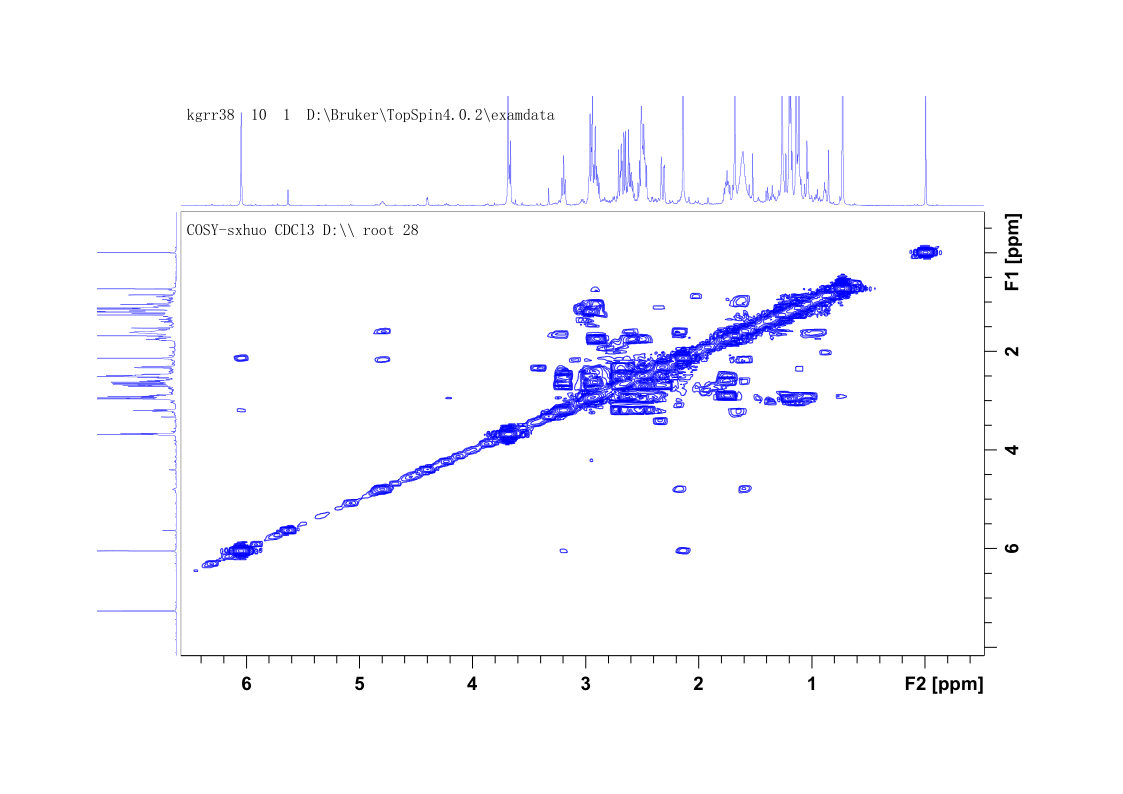


**Figure S89**. ^1^H-^1^H COSY spectrum (600 MHz, CDCl_3_) of compound **37**.


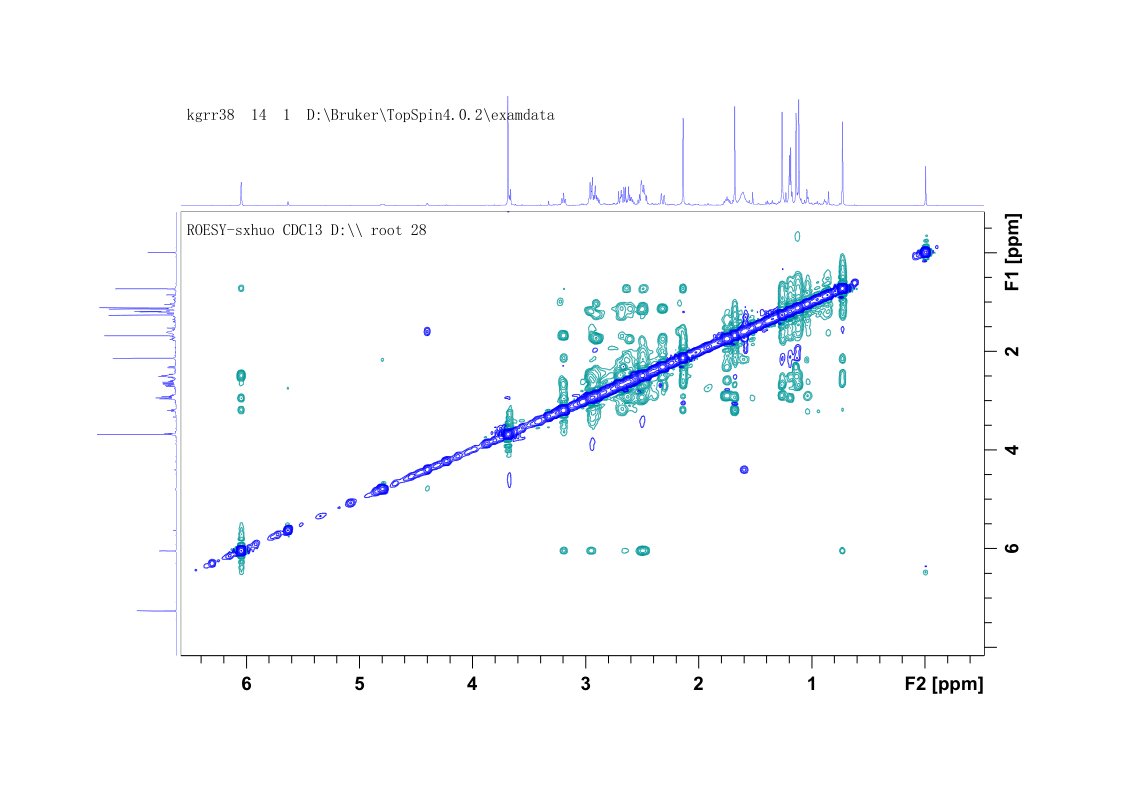


**Figure S90**. ROESY spectrum (600 MHz, CDCl_3_) of compound **37**.


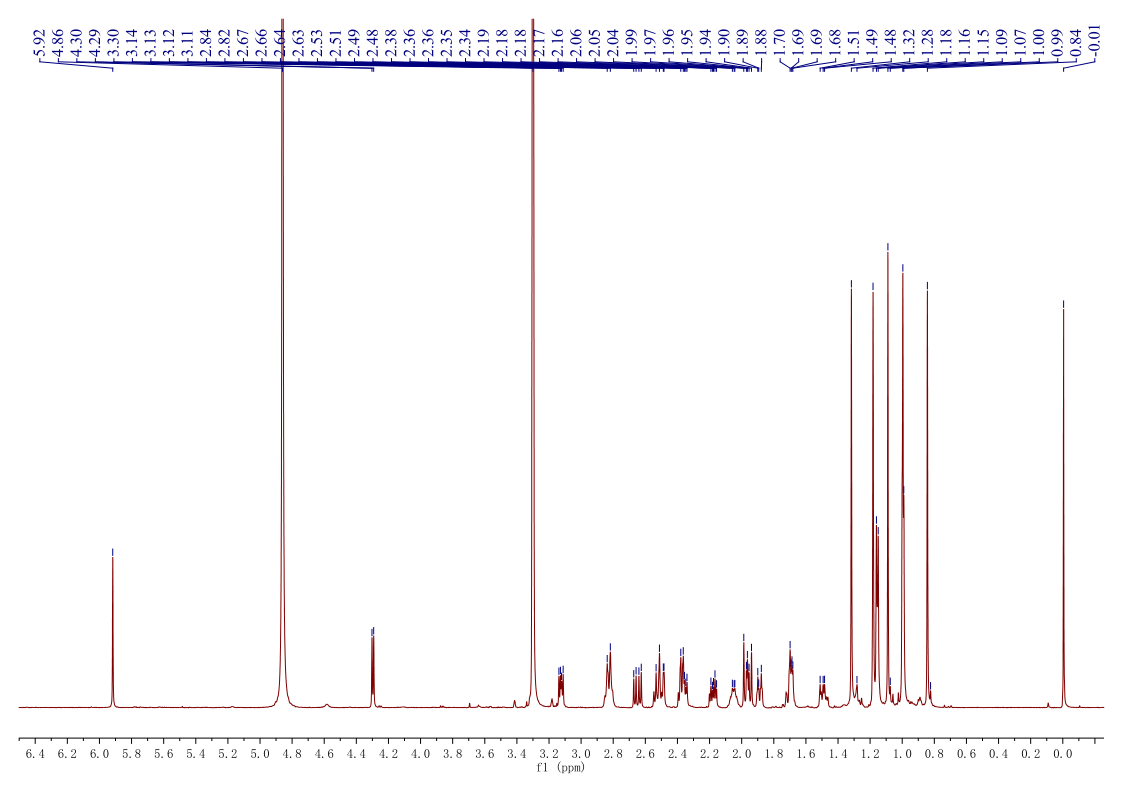


**Figure S91**. ^1^H NMR spectrum (600 MHz, CD_3_OD) of compound **42**.


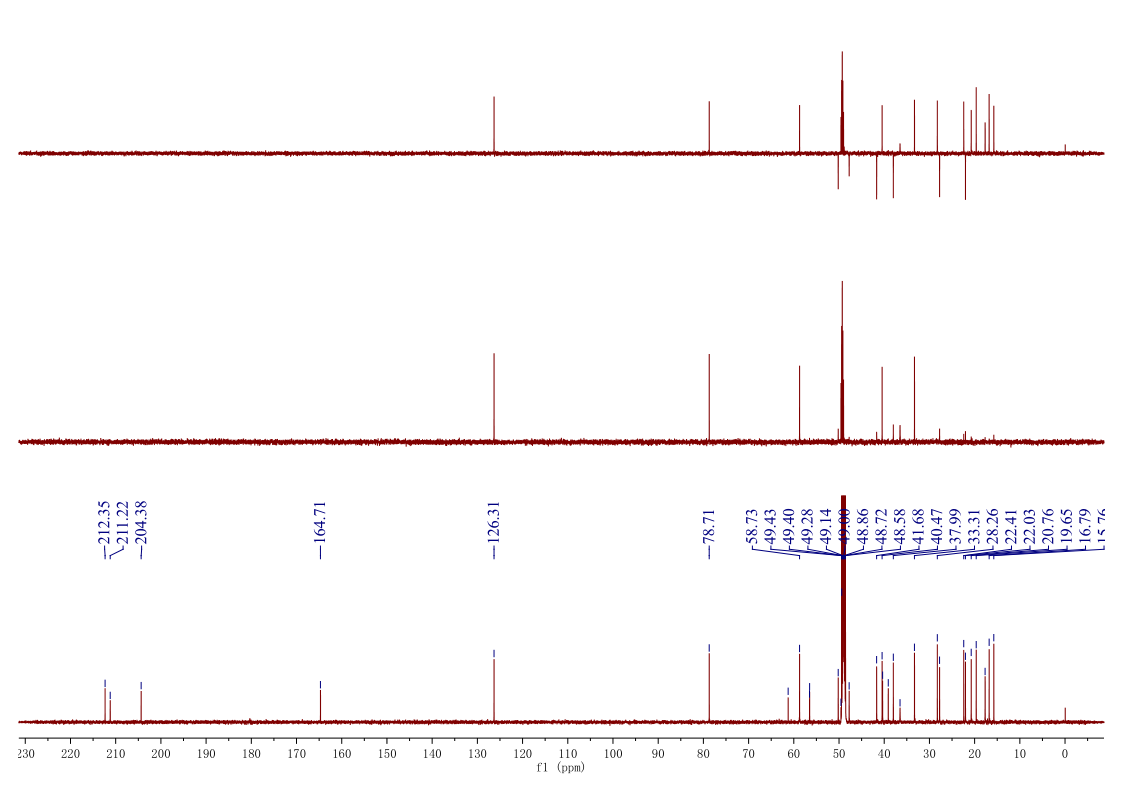


**Figure S92**. ^13^C NMR spectrum (150 MHz, CD_3_OD) of compound **42**.


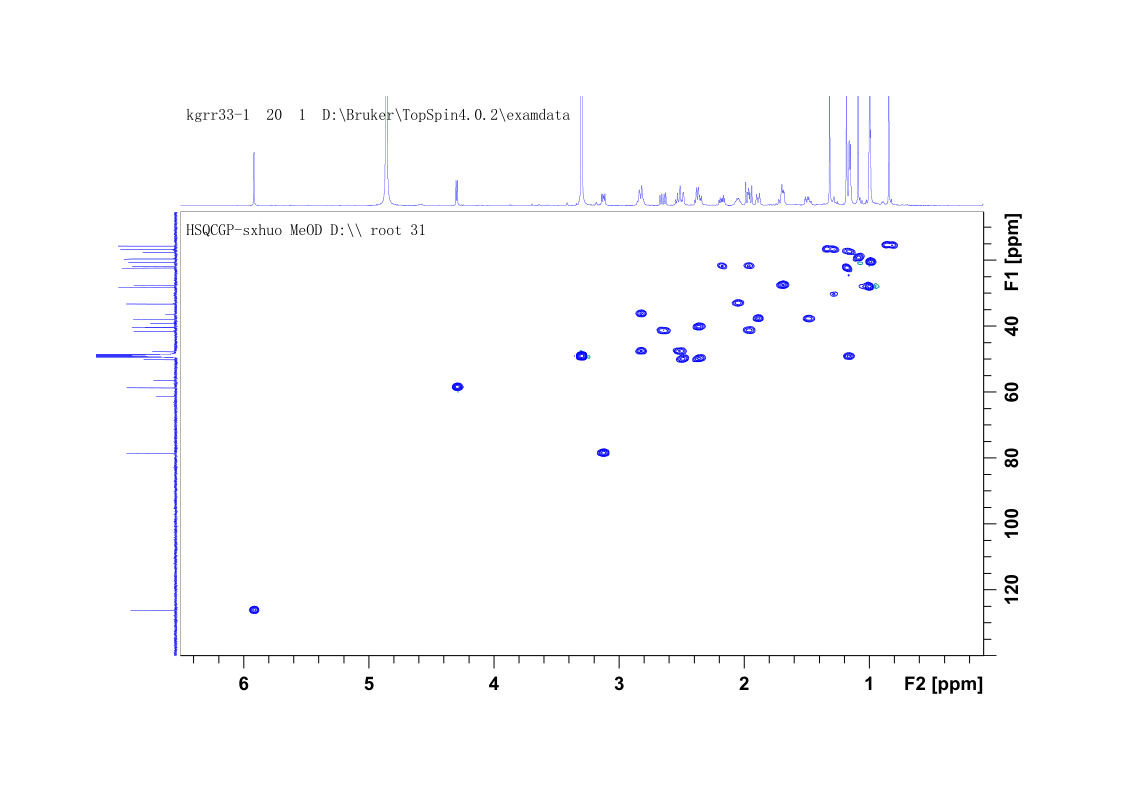


**Figure S93**. HSQC spectrum (600/150 MHz, CD_3_OD) of compound **42**.


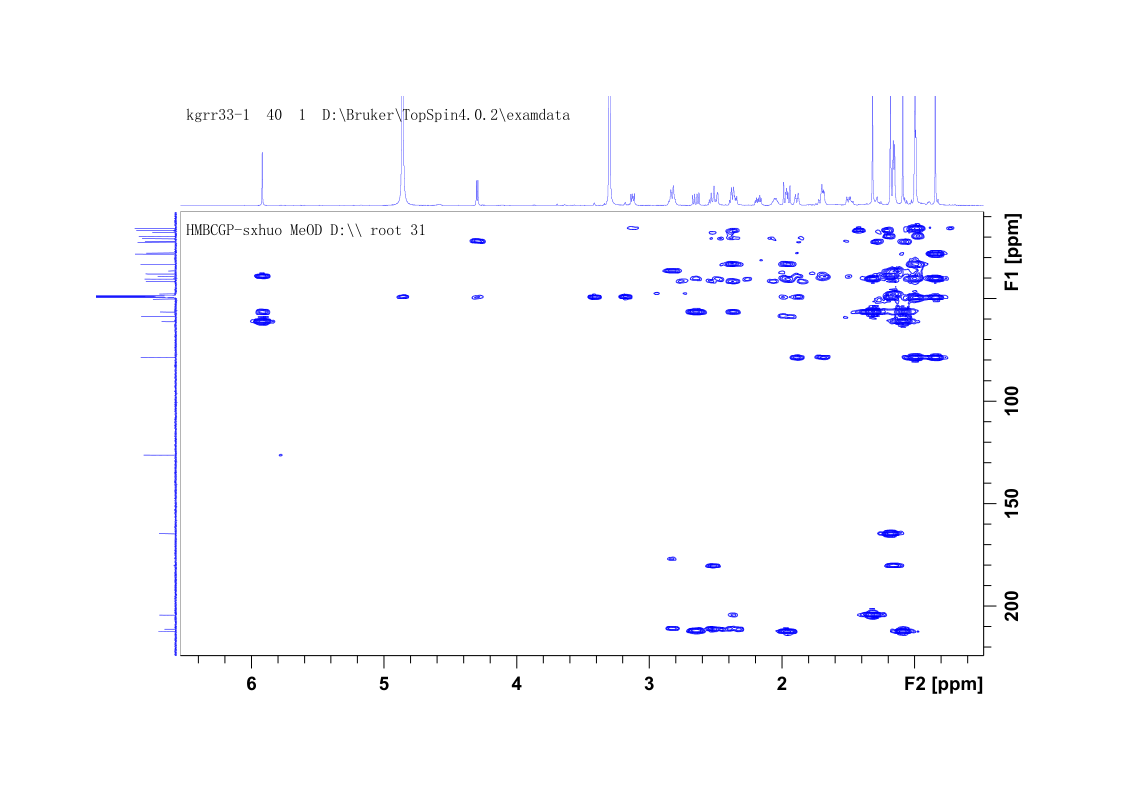


**Figure S94**. HMBC spectrum (600/150 MHz, CD_3_OD) of compound **42**.


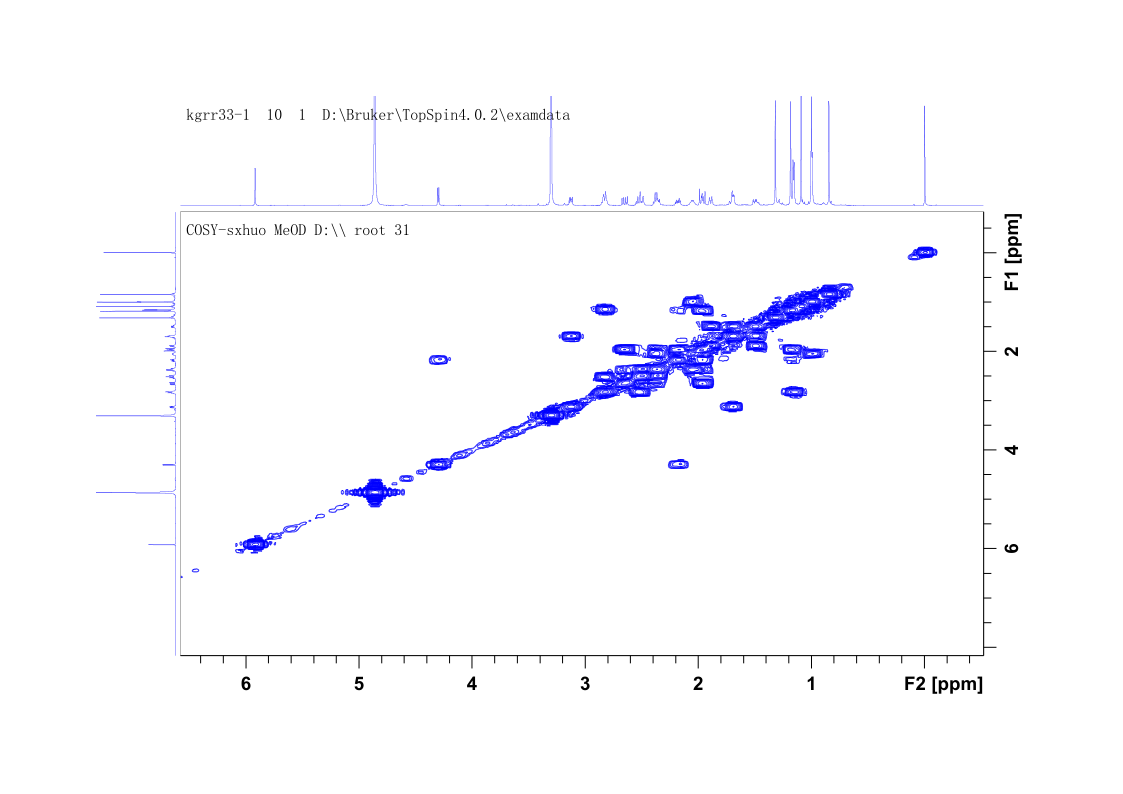


**Figure S95**. ^1^H-^1^H COSY spectrum (600 MHz, CD_3_OD) of compound **42**.


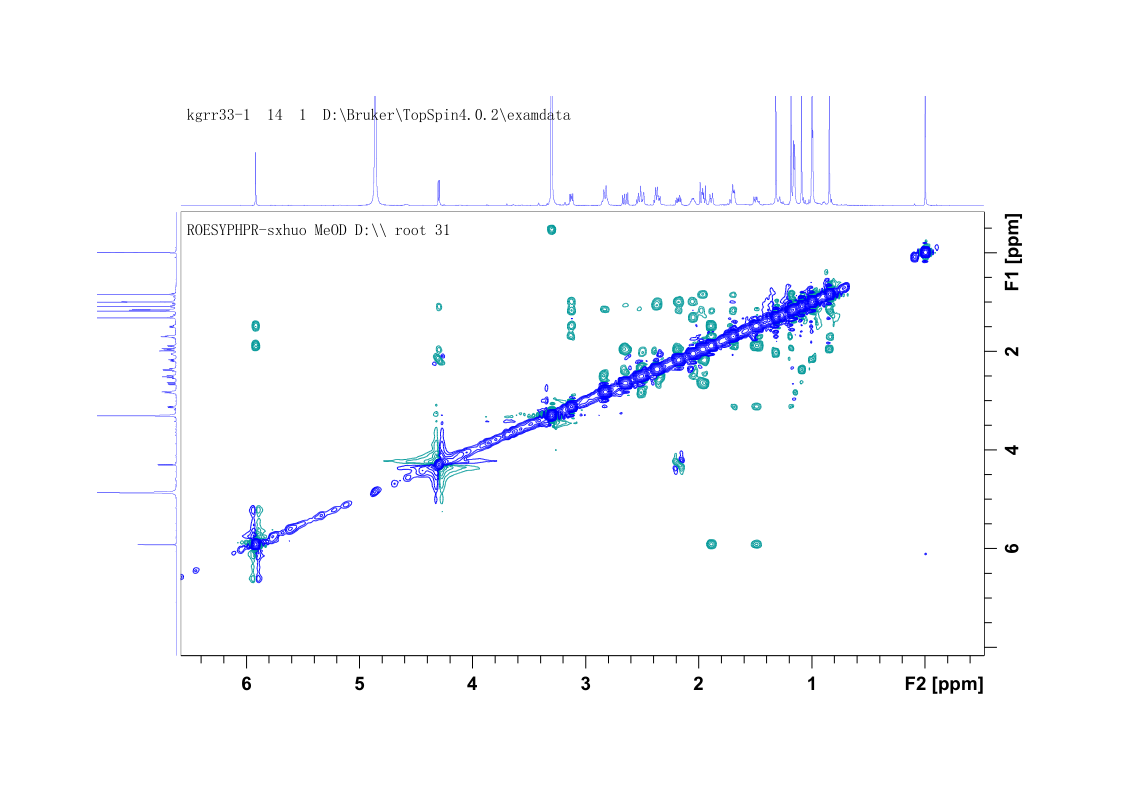


**Figure S96**. ROESY spectrum (600 MHz, CD_3_OD) of compound **42**.


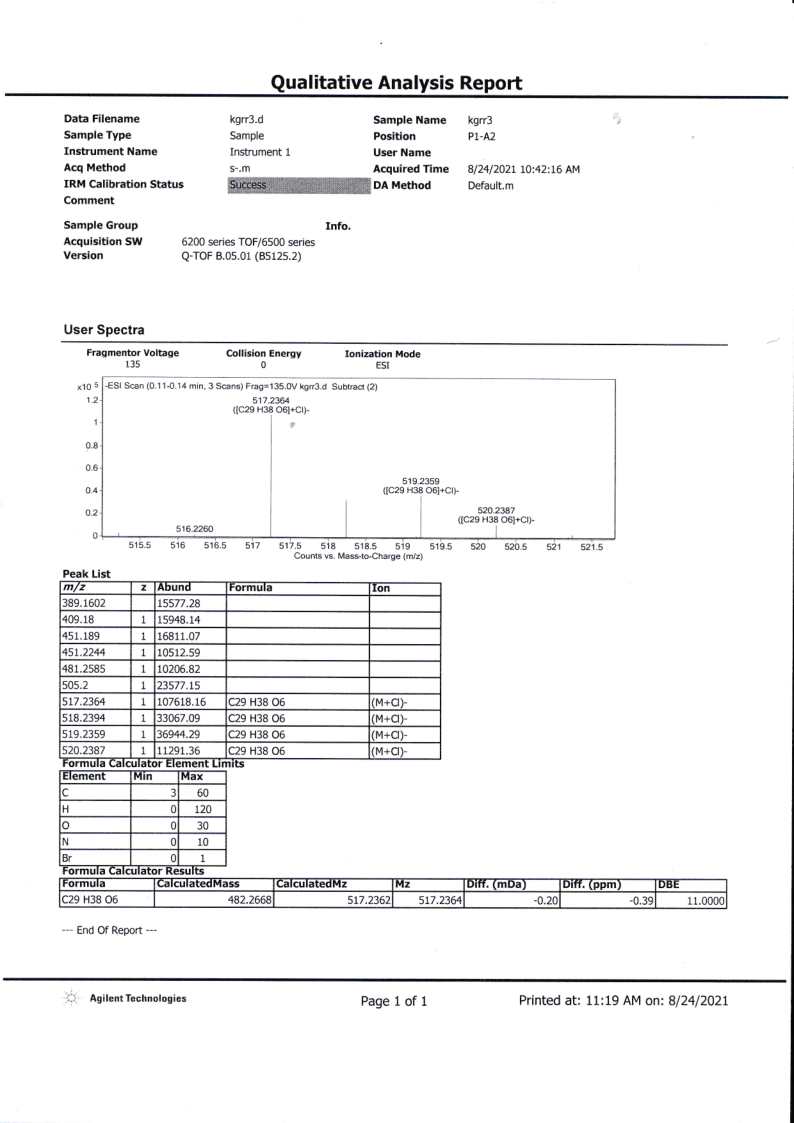


**Figure S97**. HRESIMS spectrum of compound **1**.


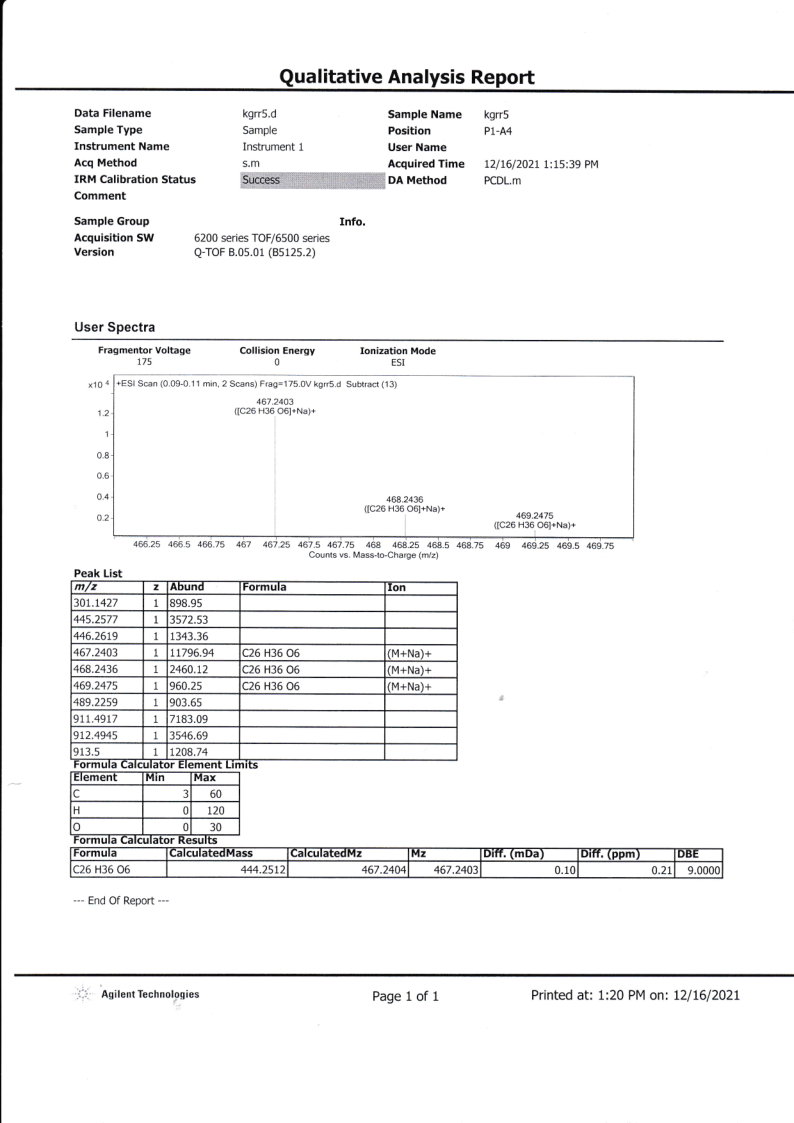


**Figure S98**. HRESIMS spectrum of compound **2**.


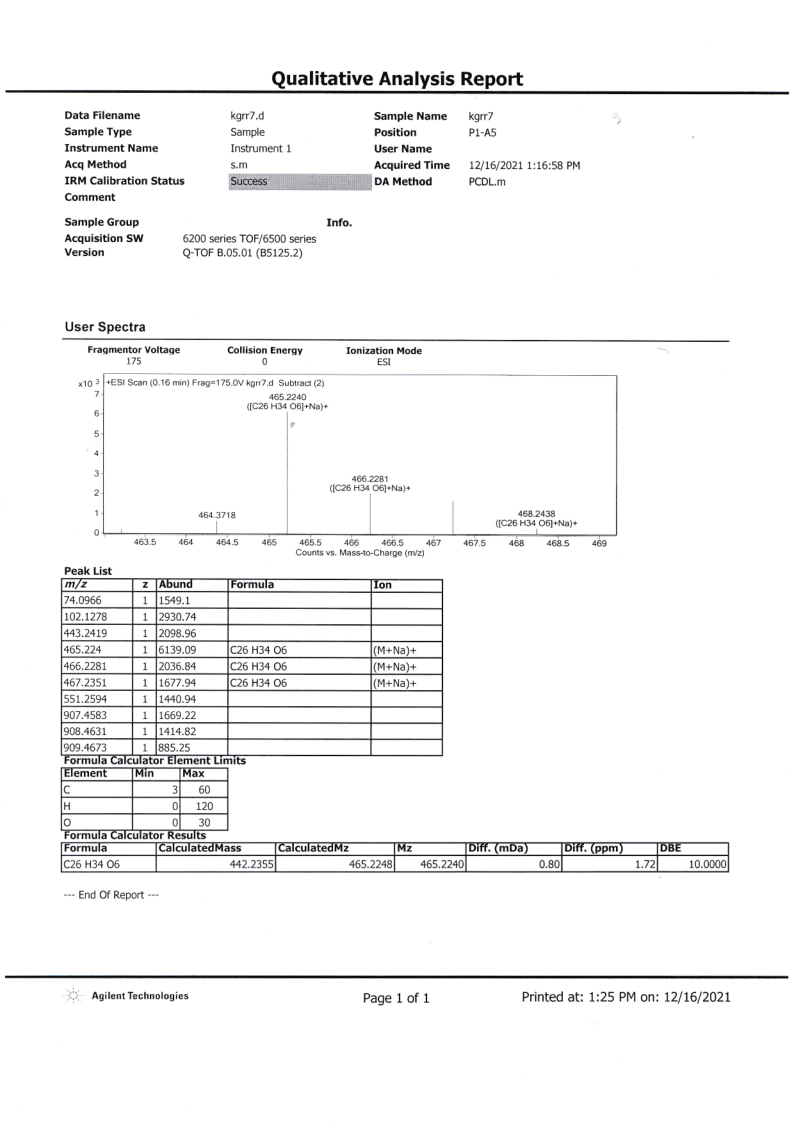


**Figure S99**. HRESIMS spectrum of compound **3**.


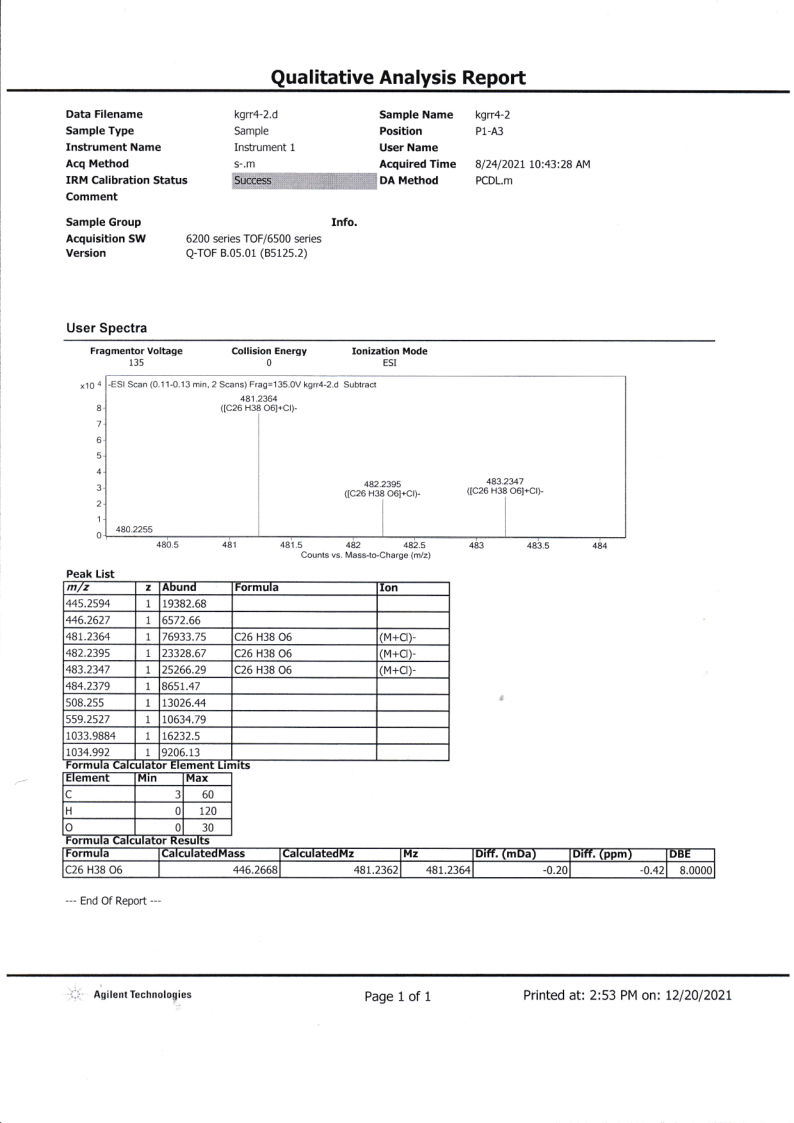


**Figure S100**. HRESIMS spectrum of compound **4**.

**Figure S101**. HRESIMS spectrum of compound **5**.

**Figure S102**. HRESIMS spectrum of compound **6**.

**Figure S103**. HRESIMS spectrum of compound **7**.

**Figure S104**. HRESIMS spectrum of compound **8**.

**Figure S105**. HRESIMS spectrum of compound **9**.

**Figure S106**. HRESIMS spectrum of compound **10**.

**Figure S107**. HRESIMS spectrum of compound **11**.

**Figure S108**. HRESIMS spectrum of compound **15**.

**Figure S109**. HRESIMS spectrum of compound **31**.

**Figure S110**. HRESIMS spectrum of compound **35**.

**Figure S111**. HRESIMS spectrum of compound **37**.

**Figure S112**. HRESIMS spectrum of compound **42**.

**Figure S113**. View of a molecule of compound **1** with the atom-labelling scheme. Displacement ellipsoids are drawn at the 30% probability level.

**Figure S114**. View of the pack drawing of compound **1**. Hydrogen-bonds are shown as dashed lines.

**Table S1**. Crystal data and structure refinement for compound **1**.

| Identification code | global |
| --- | --- |
| Empirical formula | C29 H40 O7 |
| Formula weight | 500.61 |
| Temperature | 100(2) K |
| Wavelength | 1.54178 Å |
| Crystal system | Monoclinic |
| Space group | P 1 21 1 |
| Unit cell dimensions | a = 12.2256(15) Å α = 90°  b = 7.6023(11) Å β = 111.663(6)° |
| Volume | 1273.0(3) Å3 |
| Z | 2 |
| Density (calculated) | 1.306 mg/m3 |
| Absorption coefficient | 0.748 mm-1 |
| F(000) | 540 |
| Crystal size | 0.430 x 0.170 x 0.010 mm3 |
| Theta range for data collection | 3.23 to 72.22° |
| Index ranges | -14<=h<=12, -9<=k<=9, -18<=l<=18 |
| Reflections collected | 18906 |
| Independent reflections | 4939 [R(int) = 0.0900] |
| Completeness to theta = 72.22° | 99.2 % |
| Absorption correction | Semi-empirical from equivalents |
| Max. and min. transmission | 0.99 and 0.75 |
| Refinement method | Full-matrix least-squares on F2 |
| Data / restraints / parameters | 4939 / 1 / 333 |
| Goodness-of-fit on F2 | 1.092 |
| Final R indices [I>2sigma(I)] | R1 = 0.0576, wR2 = 0.1501 |
| R indices (all data) | R1 = 0.0701, wR2 = 0.1639 |
| Absolute structure parameter | 0.02(13) |
| Largest diff. peak and hole | 0.505 and -0.283 e.Å-3 |

**Figure S115**. View of a molecule of compound **15**. Displacement ellipsoids are drawn at the 30% probability level.

**Figure S116**. View of the pack drawing of compound **15**. Hydrogen-bonds are shown as dashed lines.

**Table S2**. Crystal data and structure refinement for compound **15**.

| Identification code | global |
| --- | --- |
| Empirical formula | C24 H34 O5 |
| Formula weight | 402.51 |
| Temperature | 150(2) K |
| Wavelength | 1.54178 Å |
| Crystal system | Monoclinic |
| Space group | P 1 21 1 |
| Unit cell dimensions | a = 10.7738(3) Å b = 7.1097(2) Å c = 13.9891(4) Å |
| Volume | 1047.82(5) Å3 |
| Z | 2 |
| Density (calculated) | 1.276 Mg/m3 |
| Absorption coefficient | 0.706 mm-1 |
| F(000) | 436 |
| Crystal size | 0.420 x 0.040 x 0.020 mm3 |
| Theta range for data collection | 3.23 to 68.18°. |
| Index ranges | -12<=h<=12, -8<=k<=8, -16<=l<=16 |
| Reflections collected | 15116 |
| Independent reflections | 3777 [R(int) = 0.0909] |
| Completeness to theta = 68.18° | 99.7 % |
| Absorption correction | Semi-empirical from equivalents |
| Max. and min. transmission | 0.99 and 0.70 |
| Refinement method | Full-matrix least-squares on F2 |
| Data / restraints / parameters | 3777 / 1 / 270 |
| Goodness-of-fit on F2 | 1.101 |
| Final R indices [I>2sigma(I)] | R1 = 0.0515, wR2 = 0.1235 |
| R indices (all data) | R1 = 0.0593, wR2 = 0.1274 |
| Absolute structure parameter | 0.02(12) |
| Largest diff. peak and hole | 0.443 and -0.384 e.Å-3 |

**Scheme S1.** Possible biosynthetic pathway for types I‒V.

**Figure S117**. Cell viability of *Ganoderma* triterpenoids on HaCaT cells. One-way analysis of variance (ANOVA) with **P* < 0.05, ***P* < 0.01, ****P* < 0.005, *****P* < 0.001 vs CON. CON: Control. The data are presented as the mean ± SD. Each experiment repeats three times.

**Figure S118**. Uncropped images of western blot.

**Figure S119**. Uncropped images of western blot.

**Table S3**. Genes and PCR primers used in this study.

| **Gene** | **FORWARD** | **REVERSE** |
| --- | --- | --- |
| B-actin | ATGTGGCCGAGGACTTTGATTGC | TGTGTGGACTTGGGAGAGGACTG |
| MMP1 | TTGGGCTGAAAGTGACTGGGAAAC | CCACATCAGGCACTCCACATCTG |
| MMP3 | GGCAAGACAGCAAGGCATAGAGAC | ACGCACAGCAACAGTAGGATTGG |

**References**:

1. Peng XR, Liu JQ, Han ZH, Yuan XX, Luo HR, Qiu MH, Protective effects of triterpenoids from *Ganoderma resinaceum* on H(2)O(2)-induced toxicity in HepG2 cells, Food Chem. 2013;141(2):920-6.

2. Shi Q, Huang Y, Su H, Gao Y, Peng X, Zhou L, Li X, Qiu M, C28 steroids from the fruiting bodies of *Ganoderma resinaceum* with potential anti-inflammatory activity, Phytochemistry. 2019;168:112109-112117.

3. Chen X-Q, Chen L-X, Zhao J, Li S-P, Tang Y-P, Nortriterpenoids from the fruiting bodies of the mushroom *Ganoderma resinaceum*, Molecules. 2017;22(7):1073-1084.

4. Hennicke F, Cheikh-Ali Z, Liebisch T, Maciá-Vicente JG, Bode HB, Piepenbring M, Distinguishing commercially grown *Ganoderma lucidum* from *Ganoderma lingzhi* from Europe and East Asia on the basis of morphology, molecular phylogeny, and triterpenic acid profiles, Phytochemistry. 2016;127:29-37.

5. Nishitoba T, Sato H, Sakamura S, Bitterness and structure relationship of the triterpenoids from *Ganoderma lucidum* (Reishi), Agricul. Biol. Chem. 2016;52(7):1791-1795.

6. Qing YM, Li LH, Ling MK, Sheng ZH, Hao FD, Yan L, You XZ, Chemical constituents from the fungus *Ganoderma tropicum* (Jungh.) Bres. and their cytotoxic activities, Afr. J. Microbiol. Res. 2013;7(16):1543-1547.

7. Chen L, Chen X, Wang S, Bian Y, Zhao J, Li S, Analysis of triterpenoids in *Ganoderma resinaceum* using liquid chromatography coupled with electrospray ionization quadrupole - time - of - flight mass spectrometry, Int. J. Mass spectrom. 2019;436:42-51.

8. Chen XQ, Lin LG, Zhao J, Chen LX, Li SP, Zhao J, Luo DL, Tang YP, Isolation, structural elucidation, and α-glucosidase inhibitory activities of triterpenoid lactones and their relevant biogenetic constituents from *Ganoderma resinaceum*, Molecules. 2018;23(6):1391-1404.

9. Chen XQ, Zhao J, Chen LX, Wang SF, Wang Y, Li SP, Lanostane triterpenes from the mushroom *Ganoderma resinaceum* and their inhibitory activities against α-glucosidase, Phytochemistry (Elsevier). 2018;149:103-115.

10. Jiao Y, Xie T, Zou LH, Wei Q, Qiu L, Chen LX, Lanostane triterpenoids from *Ganoderma curtisii* and their NO production inhibitory activities of LPS-induced microglia, Bioorg. Med. Chem. Lett. 2016;26(15):3556-3561.

11. Binh PT, Nguyen PT, Nguyen TL, Trang DT, Binh PTX, Nguyen NPD, Nguyen TH, Nguyen HD, Tri TM, Nguyen TD, Lanostane-type triterpenoids from *Ganoderma lucidum* and *G. multipileum* fruiting bodies, Nat Prod Commun. 2018;13(11):1441-1444.

12. Su H-G, Peng X-R, Shi Q-Q, Huang Y-J, Zhou L, Qiu M-H, Lanostane triterpenoids with anti-inflammatory activities from *Ganoderma lucidum*, Phytochemistry (Elsevier). 2020;173:112256.

13. Liaw CC, Chen YC, Huang GJ, Tsai YC, Chien SC, Wu JH, Wang SY, Chao LK, Sung PJ, Huang HC, Kuo YH, Anti-inflammatory Lanostanoids and lactone derivatives from *Antrodia camphorata*, J. Nat. Prod. 2013;76(4):489-494.

14. Li XC, Liu F, Su HG, Peng C, Zhou QM, Liu J, Huang YJ, Guo L, Xiong L, Twelve undescribed derivatives of ganoderic acid isolated from *Ganoderma luteomarginatum* and their cytotoxicity against three human cancer cell lines, Phytochemistry. 2021;183:112617-112627.

15. Zhao XR, Zhang BJ, Deng S, Zhang HL, Huang SS, Huo XK, Wang C, Liu F, Ma XC, Isolation and identification of oxygenated lanostane-type triterpenoids from the fungus *Ganoderma lucidum*, Phytochem. Lett. 2016;16:87-91.

16. Sato N, Zhang Q, Ma CM, Hattori M, Anti-human immunodeficiency virus-1 protease activity of new lanostane-type triterpenoids from *Ganoderma sinense*, Chem. Pharm. Bull. . 2009;57:1076-1080.

17. Chairul, Tokuyama T, Hayashi Y, Nishizawa M, Chairulii. FM, Hayashi Y, Applanoxidic acids A, B, C, and D, biologically active tetracyclic triterpenes from *Ganoderma applanatum*, Phytochemistry. 1991;30:4105-4109.

18. Lei S, Zhang J, Blum NT, Li M, Zhang D-Y, Yin W, Zhao F, Lin J, Huang P, In vivo three-dimensional multispectral photoacoustic imaging of dual enzyme-driven cyclic cascade reaction for tumor catalytic therapy, Nat. Commun. 2022;13(1):1298-1302.

19. Peng X, Luo R, Ran X, Guo Y, Yao YG, Qiu M, Ganoapplins A and B with an unprecedented 6/6/6/5/6-fused pentacyclic skeleton from *Ganoderma* inhibit Tau pathology through activating autophagy, Bioorg. Chem. 2023;132:106375-106383.

20. Kwon K-R, Alam MB, Park JH, Kim TH, Lee SH, Attenuation of UVB-induced photo-aging by polyphenolic-rich spatholobus suberectus stem extract via modulation of MAPK/AP-1/MMPs signaling in human keratinocytes, Nutrients. 2019;11(6):1341-1355.

21. Zhang C, Wang H, Yang XH, Fu Z, Ji XR, Shi YF, Zhong J, Hu WG, Ye YQ, Wang ZT, Ni DL, Oral zero-valent-molybdenum nanodots for inflammatory bowel disease therapy, Sci. Adv. 2022;8(37):9882-9893.

22. Chen T, Hou H, Fan Y, Wang S, Chen Q, Si L, Li B, Protective effect of gelatin peptides from pacific cod skin against photoaging by inhibiting the expression of MMPs via MAPK signaling pathway, J. Photochem. Photobiol. B: Biol. 2016;165:34-41.
